# Mapping the Cell-Surface N-Glycoproteome of Human Hepatocytes Reveals Markers for Selecting a Homogeneous Population of iPSC-Derived Hepatocytes

**DOI:** 10.1016/j.stemcr.2016.07.016

**Published:** 2016-08-25

**Authors:** Sunil K. Mallanna, Max A. Cayo, Kirk Twaroski, Rebekah L. Gundry, Stephen A. Duncan

**Affiliations:** 1 Department of Regenerative Medicine and Cell Biology, Medical University of South Carolina, Charleston, SC 29425, USA; 2 Department of Cell Biology, Neurobiology and Anatomy, Program in Regenerative Medicine, Medical College of Wisconsin, Milwaukee, WI 53226, USA; 3 Department of Biochemistry, Medical College of Wisconsin, Milwaukee, WI 53226, USA; 4 Department of Pediatrics, University of Minnesota, Minneapolis, MN 55454, USA

## Abstract

When comparing hepatic phenotypes between iPSC-derived hepatocyte-like cells from different liver disease patients, cell heterogeneity can confound interpretation. We proposed that homogeneous cell populations could be generated by fluorescence-activated cell sorting (FACS). Using cell-surface capture proteomics, we identified a total of 300 glycoproteins on hepatocytes. Analyses of the expression profiles during the differentiation of iPSCs revealed that SLC10A1, CLRN3, and AADAC were highly enriched during the final stages of hepatocyte differentiation. FACS purification of hepatocyte-like cells expressing SLC10A1, CLRN3, or AADAC demonstrated enrichment of cells with hepatocyte characteristics. Moreover, transcriptome analyses revealed that cells expressing the liver gene regulatory network were enriched while cells expressing a pluripotent stem cell network were depleted. In conclusion, we report an extensive catalog of cell-surface N-linked glycoproteins expressed in primary hepatocytes and identify cell-surface proteins that facilitate the purification of homogeneous populations of iPSC-derived hepatocyte-like cells.

## Highlights

• Identified N-linked glycoproteins occupying surface of primary human hepatocytes
• SLC10A1, CLRN3, and AADAC are expressed on ∼25% of iPSC-derived hepatocytes
• FACS with these markers enriches hepatic character in iPSC-derived hepatocytes
• SLC10A1 sorted cells provide homogeneous populations of hepatocyte-like cells

## Teaser

Most iPSC differentiation protocols generate hepatocytes with heterogeneous expression of mature hepatic markers, which confounds analyses of complex traits and subtle phenotypes. Duncan and colleagues utilize proteomics, transcriptional profiling, and FACS to identify hepatocyte-restricted cell-surface proteins that mark a subpopulation of iPSC-derived hepatocytes, allow purification of iPSC-derived hepatocytes with enriched hepatic character, and minimize variability between differentiations.

## Introduction

Directed differentiation of pluripotent stem cells (PSCs) to cells of a specific fate holds promise to study a wide variety of human diseases (Robinton and Daley, 2012). Several groups have reported the generation of hepatocyte-like cells from human PSCs by the sequential addition of growth factors (Agarwal et al., 2008, Basma et al., 2009, Cai et al., 2007, Hay et al., 2008, Song et al., 2009, Si-Tayeb et al., 2010a, Sullivan et al., 2010). The cells produced by these approaches share many characteristics with primary hepatocytes, although transcriptional profiling has suggested that the cells in general tend to be less mature than their native counterparts (Si-Tayeb et al., 2010a). Nevertheless, induced PSCs (iPSCs) derived from patients with inborn errors in hepatic metabolism have been used to successfully model several liver diseases in culture (Rashid et al., 2010, Cayo et al., 2012, Choi et al., 2013, Tafaleng et al., 2015). Most of the liver diseases that have been successfully modeled originate from patients with Mendelian inherited mutations that show robust phenotypes. Examples include familial hypercholesterolemia and α-1-antitrypsin deficiency, which are caused by mutations in the *LDLR* (*Low-Density Lipoprotein Receptor*) and *SERPINA1* (*Serpin Peptidase Inhibitor, Clade A [Alpha-1 Antiproteinase, Antitrypsin], Member 1*) genes, respectively (Rashid et al., 2010, Cayo et al., 2012, Tafaleng et al., 2015, Choi et al., 2013). While such successes are encouraging, it has become clear that complex traits and subtle phenotypes are more challenging to reproduce, primarily due to variations in the efficiency of differentiation among cells. Most, if not all, differentiation protocols generate iPSC-derived hepatocyte-like cells with heterogeneous expression of mature hepatic markers.

The generation of antibodies that facilitate purification of homogeneous subpopulations of cells has significantly advanced the study of lymphoid lineages and hematopoietic stem cells (Lanier et al., 1983, Spangrude et al., 1988). More recently, similar approaches have been used to identify and characterize subpopulations of liver cells in mice with progenitor cell characteristics (Dorrell et al., 2008, Dorrell et al., 2011). Antibodies that recognize cell-surface antigens have historically been generated by immunization of recipient animals with whole cells or membrane fractions. Antibodies that specifically recognize a given cell type are then identified by secondary screens. This procedure has recently been used successfully to identify monoclonal antibodies that can detect iPSC-derived endoderm cells that have an enhanced propensity to adopt a hepatic fate (Holtzinger et al., 2015). Although such an approach has been invaluable, the nature of the antigen can be complex and identification of epitopes challenging.

As an alternative to antibody-based methods, we used the chemoproteomic cell surface capture technology (CSC-Technology; Wollscheid et al., 2009, Gundry et al., 2012), which incorporates selective labeling, enrichment, and mass-spectrometry-based identification of extracellular domains of cell-surface N-glycoproteins to discover accessible surface markers on human hepatocytes. By establishing a “surfaceome” of human hepatocytes, this resource reveals targets for fluorescence-activated cell sorting (FACS)-based isolation, contributes to our knowledge of N-glycoproteins that define the hepatocyte phenotype, and is a first step toward revealing N-glycoproteins with potentially important roles in hepatocyte function and disease. Using a combination of proteomic and transcriptional profiling along with antibody-based sorting, we revealed that SLC10A1, CLRN3, and AADAC are cell-surface N-glycoproteins that are present on a common population of iPSC-derived hepatocyte-like cells that express elevated levels of several hepatic markers. Transcriptional profiling of purified SLC10A1-positive cells revealed enrichment of hepatic character, improved homogeneity, and depletion of cells expressing pluripotent markers.

## Results

### Cell-Surface N-Glycoproteins on Primary Human Hepatocytes

**Figure 1:**
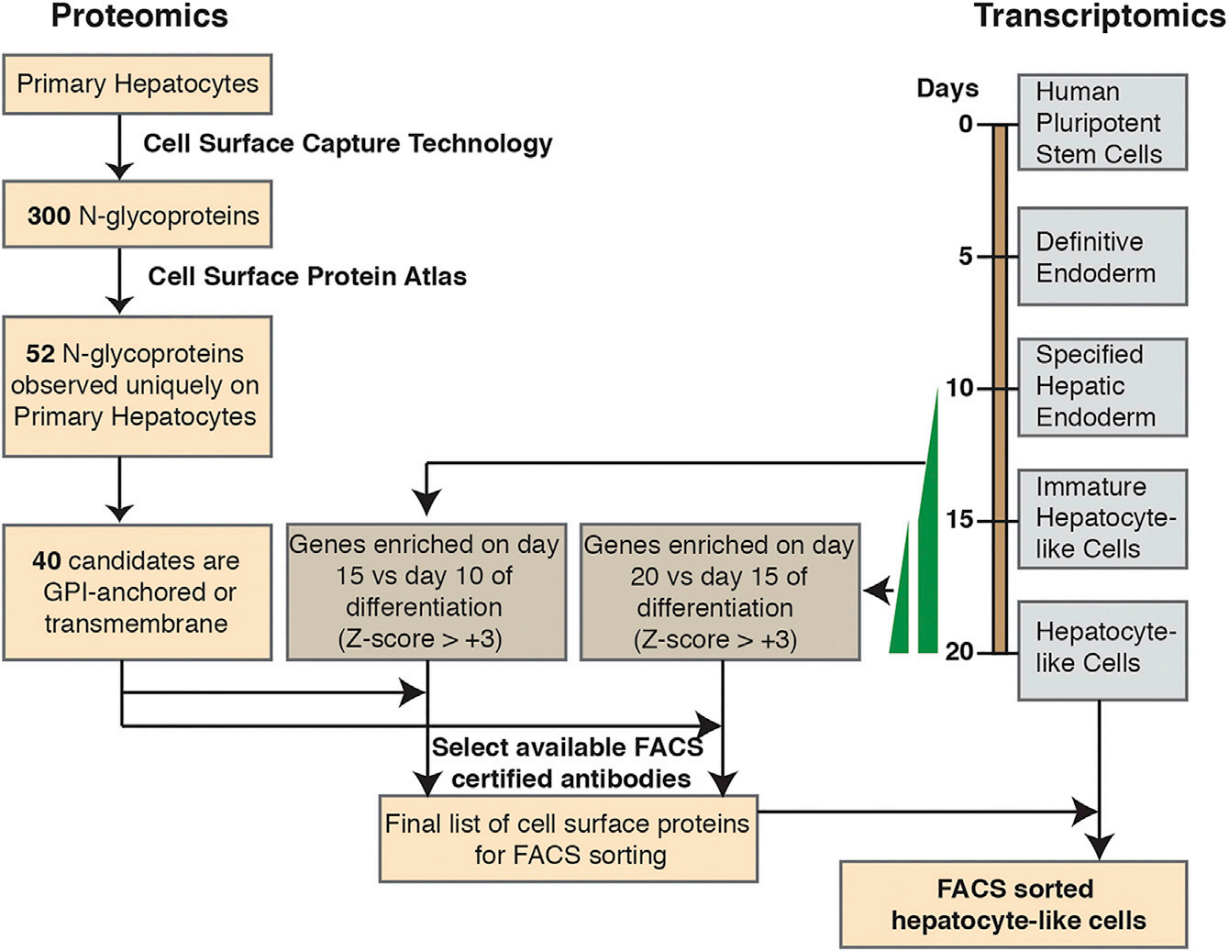
Identification of Cell-Surface Proteins for Purifying a Subpopulation of iPSC-Derived Hepatocyte-like Cells A schematic overview of the approach used to identify cell-surface N-glycoproteins and candidate markers for hepatocyte sorting. Proteomic and oligonucleotide array analyses were combined to identify cell-surface N-glycoproteins that are enriched in hepatocyte-like cells that are derived from iPSCs.

**Figure 2:**
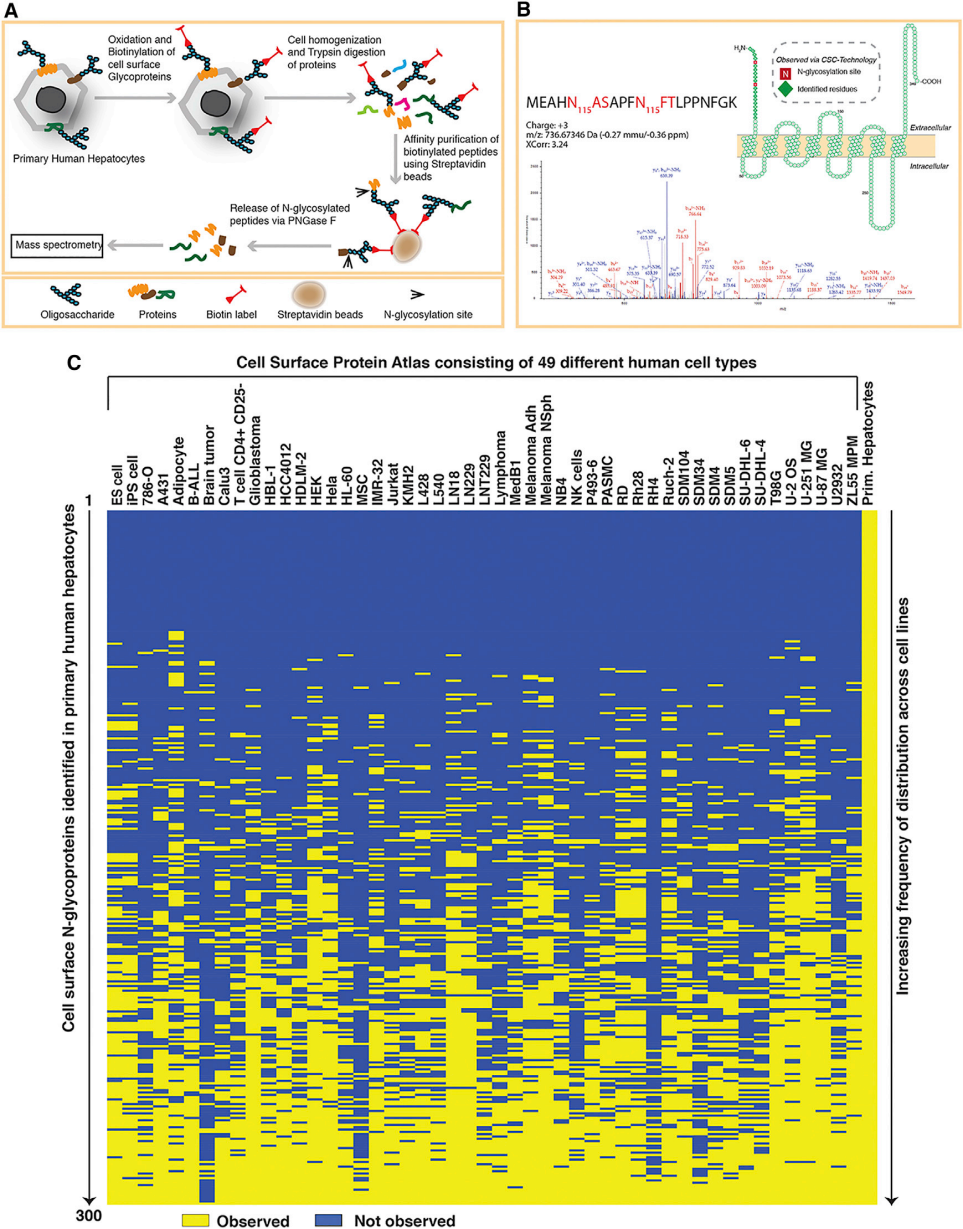
Identification of N-Linked Glycoproteins in Primary Hepatocytes by Cell Surface Capture Technology (A) Illustration outlining the CSC-Technology. (B) Left: Annotated MS/MS spectrum of a representative peptide from SLC10A1 that was identified in primary hepatocytes by CSC-Technology. Two identified sites of N-glycosylation (N_115_) are indicated. Right: An illustration of the transmembrane topology of SLC10A1 highlighting the extracellular peptide identified by CSC-Technology, including sites of N-glycosylation (also see Figure S2). Image generated using Protter (Omasits et al., 2014). (C) Graphical representation of the distribution of proteins identified in primary hepatocytes. Each column represents an individual cell type documented in the Cell Surface Protein Atlas, which consists of 49 different cell types. The last column shows proteins identified on the surface of primary hepatocytes described in the current study. Each row represents a single protein. A yellow bar indicates the protein was reported as present in the cell line, whereas blue indicates that it was not observed. The frequency of distribution of each protein is lowest at the top and highest at the bottom. The identity of each protein in the order presented is provided in Table S1.

To identify cell-surface N-glycoproteins that could potentially be used to purify homogeneous populations of iPSC-derived hepatocytes, we incorporated proteomic and transcriptomic analyses of primary hepatocytes and iPSC-derived cells (Figure 1). We first used the CSC-Technology to identify extracellular domains of cell-surface N-glycoproteins on primary human hepatocytes. This approach (Figure 2A) exploits biotinylation of the oligosaccharide structure attached to the extracellular domain of cell-surface glycoproteins on live cells. Following enzymatic digestion, biotinylated glycopeptides are enriched by affinity chromatography using immobilized streptavidin. Finally, the enzyme N-glycosidase F (PNGaseF), which cleaves between the innermost GlcNAc and the asparagine residue, selectively releases the formerly N-glycosylated peptides for subsequent analysis by high mass accuracy mass spectrometry analysis. Overall, this strategy provides for highly specific identification of cell-surface N-glycoproteins, including transmembrane, glycophosphatidylinositol (GPI)-anchored, and extracellular matrix, with minimal contamination from intracellular membrane proteins (Wollscheid et al., 2009, Bausch-Fluck et al., 2015, Boheler et al., 2014).

Proteins identified in this workflow are confidently assigned as originating from the cell surface if they are identified by at least one peptide containing a deamidated asparagine (resulting from PNGaseF cleavage) within the conserved sequence motif for N-glycosylation (NxS/T/C), although the majority of N-glycoproteins identified here are represented by multiple peptides (Table S1). An annotated tandem mass spectrometry (MS/MS) spectrum for a peptide derived from SLC10A1, illustrating the deamidation within the sequence motif is shown in Figure 2B. Using the above criteria, 300 cell-surface N-glycoproteins, including 66 cluster of differentiation (CD) molecules were identified on primary human hepatocytes (Figure 2C). A complete list of all N-glycoproteins that were identified is provided in Table S1. Functional annotation using DAVID reveals that of the 300 N-glycoproteins identified, 292 are classified as glycoproteins and 228 as transmembrane proteins (Figure S1A). Gene ontology analyses revealed, as expected, that functions typically associated with transmembrane proteins, including receptors, signal transduction, and transporter activity, were highly represented (Figure S1B).

### Identification of N-Glycoproteins That Are Enriched in Primary Hepatocytes

As discussed above, the ability to generate hepatocyte-like cells from iPSCs has enabled the modeling of inborn errors of hepatic metabolism in culture (Cayo et al., 2012, Rashid et al., 2010). However, heterogeneity in the differentiated cell population can complicate the interpretation of results. We reasoned that relatively homogeneous populations of cells could be generated by FACS using antibodies that recognize cell-surface N-glycoproteins that are enriched in hepatocytes. To date, Asialoglycoprotein Receptor 1 (ASGR1) has been used for hepatocyte enrichment (Basma et al., 2009, Peters et al., 2016). Although ASGR1 is highly expressed in the liver, it is also abundant in the gall bladder (Wilhelm et al., 2014). Moreover, studies of hematopoietic cells have revealed that increasing the repertoire of cell-surface proteins available for sorting promotes specificity (Lanier et al., 1983, Spangrude et al., 1988). We, therefore, sought to identify additional proteins whose presence on the hepatocyte cell surface may facilitate the production of homogeneous populations of hepatocyte-like cells from differentiating iPSCs.

We reasoned that N-glycoproteins whose expression was robust and restricted to hepatocytes would be the most suitable for purifying hepatocytes from iPSCs. An extensive searchable database, the Cell Surface Protein Atlas (Atlas), which presents the surfaceome of a broad variety of 47 human cell types, has recently been made available (Bausch-Fluck et al., 2015). We exploited this database along with surfaceome of human embryonic stem cells (ESCs) and iPSCs to obtain the frequency of detection of each protein identified in primary hepatocytes (Figure 2C) (Boheler et al., 2014). Protein distribution frequency ranged from proteins detected only in primary hepatocytes to those whose presence was ubiquitous. As with any mass spectrometry, failure to identify a particular protein does not irrefutably confirm the absence of that protein from the surface of that cell type. Rather, this resource is most valuable as a first step to eliminate proteins that are observed across many cell types and to narrow the focus to proteins that may be informative of a cell type of interest. Of the 300 N-glycoproteins identified, 52 were detected only in primary hepatocytes and 40 of those were annotated as transmembrane or GPI-anchored proteins (Figure 2C; Table S1). As our goal was to identify cell-surface N-glycoproteins that could be used for live cell sorting, we focused our subsequent analyses on these 40 hepatocyte-enriched transmembrane or GPI-anchored proteins (Table 1).

### A Subset of Hepatocyte-Enriched N-Glycoproteins Are Induced during the Differentiation of Human Pluripotent Stem Cells into Hepatocyte-like Cells

**Figure 3:**
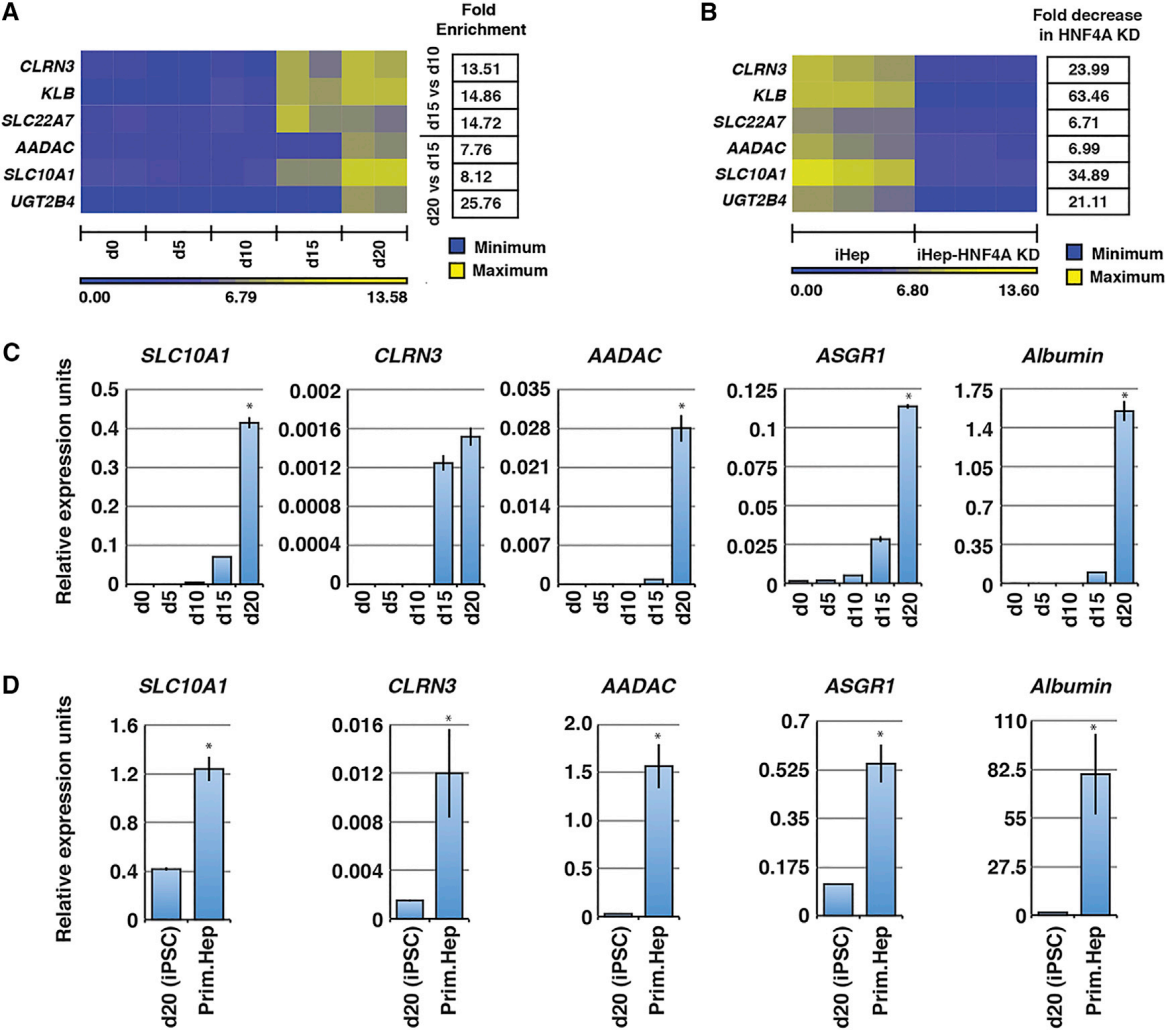
Selection of Cell Surface Proteins for Purifying iPSC-Derived Hepatocyte-like Cells (A) Heatmap showing enrichment of six transcripts encoding hepatocyte-restricted cell surface glycoproteins (CLRN3, KLB, SLC22A7, AADAC, SLC10A1, and UGT2B4) during the differentiation of human ESCs to hepatocyte-like cells. Published oligonucleotide array data from biological replicates at days 0, 5, 10, 15, and 20 of differentiation were analyzed (Si-Tayeb et al., 2010a, Delaforest et al., 2011). Fold enrichment of the six transcripts between days 10 and 15 or days 15 and 20 is given next to the heatmap. (B) Published oligonucleotide expression data were used to examine transcript levels encoding CLRN3, KLB, SLC22A7, AADAC, SLC10A1, and UGT2B4 in hepatocyte-like cells generated from wild-type ESCs and ESCs in which HNF4A is depleted (Delaforest et al., 2011). Fold decrease in the level of transcripts following HNF4A depletion is given next to the heatmap. (C) Bar graphs of qRT-PCR results showing levels of mRNAs encoding cell-surface proteins SLC10A1, CLRN3, AADAC, ASGR1, and albumin on days 0, 5, 10, 15, and 20 of differentiation of iPSCs into hepatocyte-like cells. Data are represented as means ± SEM of biological replicates (n = 3). Significance of enrichment in day 20 cells compared with day 15 cells was determined by Student's t test, ^∗^p < 0.05. (D) Bar graphs of qRT-PCR results showing relative expression levels of mRNAs encoding SLC10A1, CLRN3, AADAC, ASGR1, and albumin in iPSC-derived hepatocyte-like cells in comparison with primary hepatocytes (see also Figure S3A). Data are represented as means ± SEM of biological replicates (n = 3). Significance was determined by Student's t test, ^∗^p < 0.05.

To further narrow the 40 candidates down to those with the most potential for sorting iPSC-derived hepatocyte-like cells, we focused on those N-glycoproteins for which mRNA levels are most robustly expressed in iPSC-derived hepatocyte-like cells and induced during the final stages of differentiation when the hepatocytes reach relative maturity (Figure 1). We have previously described a protocol for the efficient and reproducible differentiation of human ESCs and iPSCs into hepatocyte-like cells using defined culture conditions (Si-Tayeb et al., 2010a, Mallanna and Duncan, 2013) The procedure recapitulates the stages of hepatocyte differentiation that occur during embryogenesis. Definitive endoderm is produced by day 5, endoderm is converted to a hepatic fate by day 10, fetal hepatocyte-like cells are formed by day 15, and cells with more mature hepatocyte characteristics are generated by day 20 (Mallanna and Duncan, 2013, Si-Tayeb et al., 2010a, Yu et al., 2012). We recently established mRNA profiles from each stage of the differentiation process using oligonucleotide arrays (Si-Tayeb et al., 2010a, Delaforest et al., 2011). Using this approach, we examined the temporal expression pattern of mRNAs encoding the 40 hepatocyte-enriched cell-surface N-glycoproteins during differentiation. The mRNA levels encoding six of the proteins (CLRN3, KLB, SLC22A7, AADAC, SLC10A1, and UGT2B4) were found to be enriched ≥8-fold either by day 15 or day 20, coincident with when the cells establish hepatocyte function (Figure 3A). We finally used a genetic approach to examine whether the increases in these mRNA levels was due to expression in hepatocytes (Figure 3B). The transcription factor HNF4A is essential for hepatocyte differentiation and when HNF4A is depleted, PSCs are incapable of adopting a hepatic fate (Delaforest et al., 2011, Li et al., 2000, Parviz et al., 2003). We predicted that if the mRNAs encoding the candidate N-glycoproteins were selectively expressed in ESC-derived hepatocytes, the relative abundance of these mRNAs should be depleted in the absence of HNF4A. As shown in Figure 3B, in contrast to day 20 hepatocytes derived from control ESCs in which mRNAs encoding the six N-glycoproteins were readily detected by oligonucleotide array analyses, these same mRNAs were undetectable in hepatocytes derived from HNF4A-depleted ESCs.

### SLC10A1, CLRN3, and AADAC Are Co-expressed in a Subset of iPSC-Derived Hepatocytes

Of the six N-glycoproteins that were selected as hepatocyte markers, commercially available antibodies that were compatible with FACS were available for SLC10A1, CLRN3, and AADAC. Representative MS/MS spectra and peptide sequences from the CSC-Technology analyses are provided for each of these proteins (Figure S2) and all corresponding peptide details are in Table S1. Oligonucleotide array analyses performed on differentiating ESCs indicated that the mRNAs encoding these proteins were induced as the cells approached maturity (day 20; Figure 3A). We therefore attempted to confirm these expression profiles on independent differentiations of iPSCs by qRT-PCR. As expected, like *Albumin* and *ASGR1*, which were used as markers of differentiation, *SLC10A1, CLRN3*, and *AADAC* mRNAs were close to undetectable in PSCs (day 0), definitive endoderm cells (day 5), and hepatic progenitor cells (day 10) (Figure 3C). Consistent with the oligonucleotide array data, we observed a large induction of *CLRN3* mRNA at day 15, which continued through day 20. *SLC10A1* and *AADAC* transcript levels remained low at day 15 then increased substantially by day 20 of differentiation (Figure 3C). Although *SLC10A1, CLRN3*, and *AADAC* mRNAs were reproducibly induced as the iPSC-derived hepatocytes entered a maturation phase, it is important to note that a comparison of the mRNA levels found in iPSC-derived hepatocytes with those found in primary hepatocytes revealed them to be significantly lower in the iPSC- and ESC-derived cells (Figure 3D). Similar results were obtained when qRT-PCR was performed on hepatocyte-like cells derived from either H1 (WA01) or H9 (WA09) human ESCs (Figure S3A).

**Figure 4:**
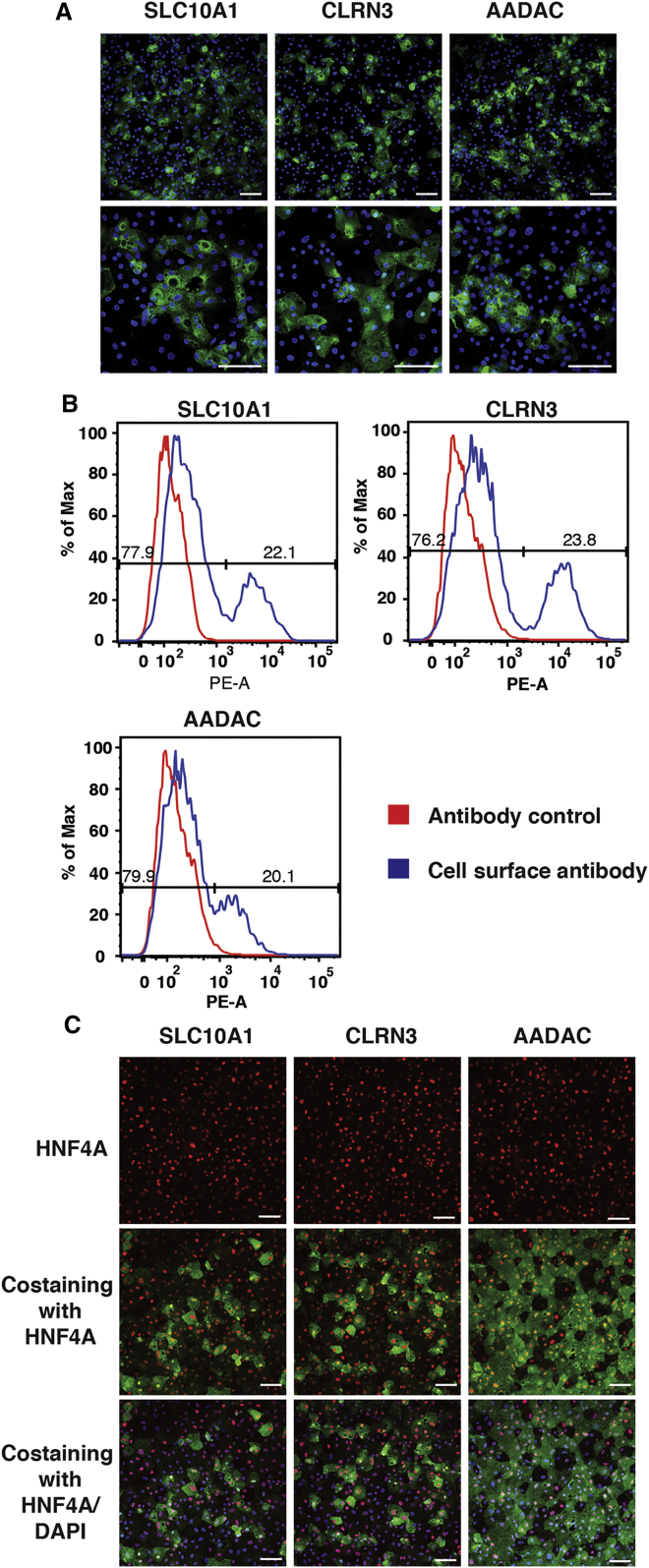
A Subpopulation of iPSC-Derived Hepatocyte-like Cells Express SLC10A1, CLRN3, and AADAC (A) Confocal micrographs showing the results of immunocytochemistry to detect expression of cell-surface proteins SLC10A1, CLRN3, and AADAC in iPSC-derived hepatocyte-like cells (green). Nuclei are identified by DAPI staining (blue) (see also Figures S3B and S3C). Scale bars, 100 μm. (B) FACS histogram plots of iPSC-derived hepatocyte-like cells stained with primary antibodies against proteins SLC10A1, CLRN3, and AADAC and corresponding phycoerythrin (PE) conjugated secondary antibody are shown. Cells stained with PE conjugated secondary antibody only are used as control. (C) Confocal micrographs showing the results of immunocytochemistry to detect expression of HNF4A (red) and cell-surface proteins SLC10A1, CLRN3, and AADAC (green) in iPSC-derived hepatocyte-like cells (see also Figure S4). Nuclei are identified by DAPI staining (blue). Scale bars, 100 μm.

We reasoned that the relatively low levels of mRNAs encoding SLC10A1, CLRN3, and AADAC observed in the iPSC-derived hepatocytes could be due to low expression throughout the entire population of cells or alternatively that expression is restricted to a subpopulation. To distinguish between these possibilities, we examined the cellular distribution of SLC10A1, CLRN3, and AADAC proteins in iPSC-derived hepatocytes by immunocytochemistry and live cell flow cytometry (Figure 4). Confocal imaging of iPSC-derived hepatocytes revealed that the target proteins were uniformly detected throughout the cell membranes but were present on a subpopulation of differentiated cells (Figure 4A). Next, flow cytometry was used to quantify the percent positive population. These analyses revealed that 20%–25% of the total population was positive for each of these cell-surface N-glycoproteins (Figure 4B). To confirm the identity of the SLC10A1-, CLRN3-, and AADAC-positive cells, co-staining experiments using an antibody that recognizes the hepatocyte transcription factor HNF4A were performed. By day 20 of differentiation, >90% of cells expressed HNF4A (Figure 4C). However, while nearly all of the SLC10A1-, CLRN3-, or AADAC-positive cells were also positive for HNF4A, only a subpopulation of HNF4A-positive cells were positive for SLC10A1, CLRN3, or AADAC (Figure 4C; note that fixation conditions required to detect HNF4A resulted in non-specific binding of the anti-AADAC antibody). Pairwise co-staining revealed that SLC10A1, CLRN3, and AADAC are expressed on the same subpopulation of iPSC-derived hepatocytes (Figure S3B).

All these experiments were performed using a single iPSC line (iPSC-K3) that was derived from foreskin fibroblasts as we have described previously (Si-Tayeb et al., 2010b). To exclude the possibility that the heterogeneous expression of SLC10A1, CLRN3, and AADAC reflected any peculiarity of K3 cells, we repeated our analyses on hepatocytes derived from an independent iPSC line (SV20) that was generated from peripheral blood mononuclear cells from an independent donor (Yang et al., 2015). Similar to using K3 iPSCs, SLC10A1, CLRN3, and AADAC were co-expressed in ∼25% of SV20 iPSC-derived hepatocytes (Figure S3C). Based on these results, we conclude that SLC10A1, CLRN3, and AADAC are expressed on a common subpopulation of iPSC-derived hepatocytes. Since ASGR1 has been used by others to purify iPSC-derived hepatocytes by FACS, we compared the distribution of ASGR1 protein with SLC10A1, CLRN3, and AADAC by immunostaining. Although ASGR1 was more broadly expressed, all SLC10A1, CLRN3, and AADAC positive cells also expressed ASGR1 (Figure S4). These results confirm that although the vast majority of cells derived from the iPSCs have adopted a hepatic fate and are HNF4A positive, expression of mature hepatic markers is heterogeneous.

### SLC10A1 Purified iPSC-Derived Hepatocytes Demonstrate Improved Hepatic Phenotype, Depletion of Pluripotent Cell Markers, and Increased Homogeneity

**Figure 5:**
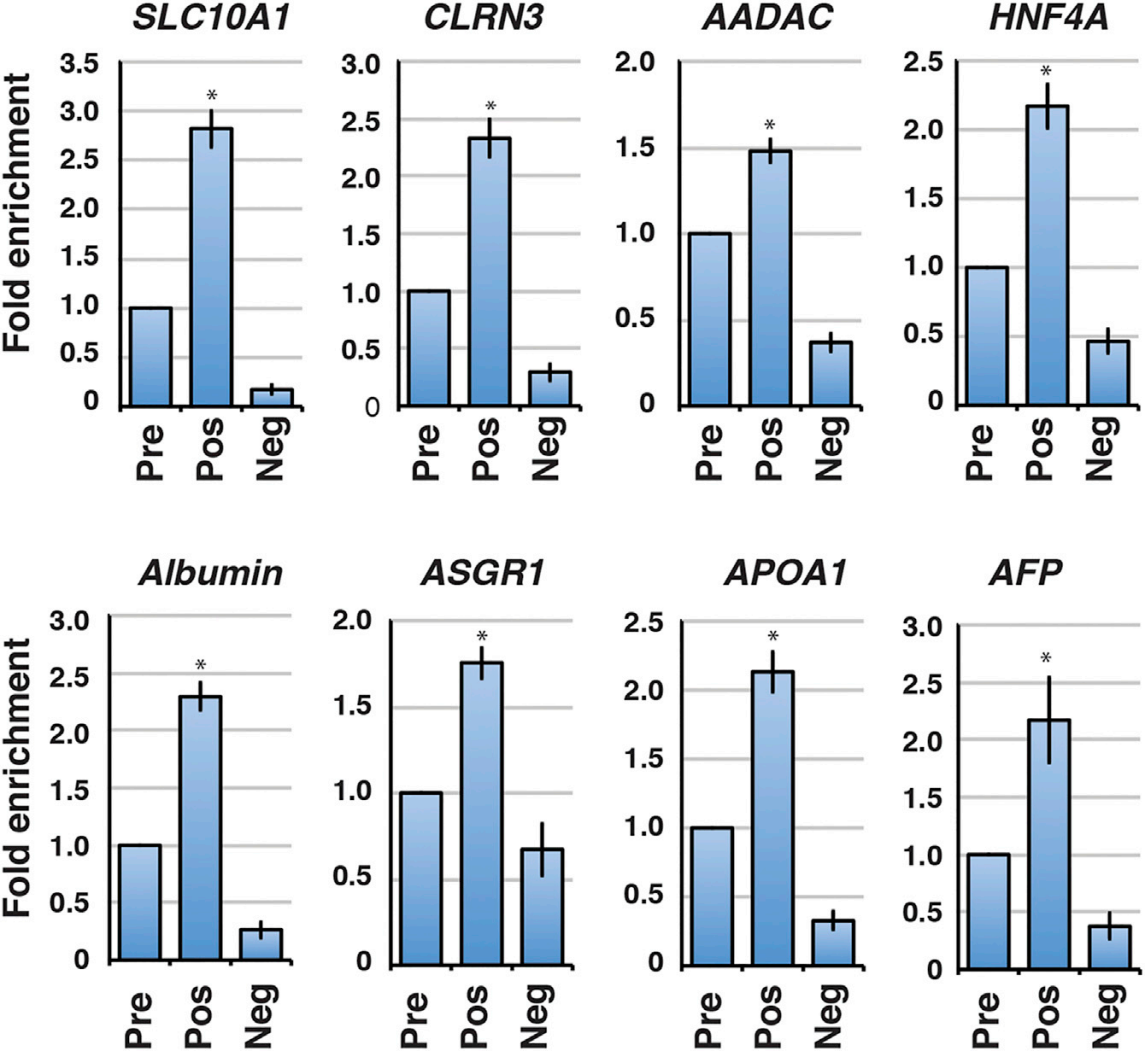
A Subpopulation of iPSC-Derived Hepatocyte-like Cells that Express SLC10A1 Is Enriched for Several Liver-Specific Markers iPSC-derived hepatocyte-like cells were sorted by FACS using an antibody against SLC10A1 (see also Figure S5A). Bar graphs show qRT-PCR analysis to determine the levels of mRNAs encoding the liver-enriched proteins (HNF4A, albumin, ASGR1, APOA1, AFP) and cell-surface proteins (SLC10A1, CLRN3, AADAC) in the SLC10A1-positive and -negative cell fractions. Levels of mRNAs were compared with the pre-sorted cell population that is set to 1.0 (see also Figures S5B–S5D). To avoid any bias introduced by the sorting process, the pre-sort population was collected as the gated viable cell fraction after passing through the FACS. Data are represented as means ± SEM from sorting performed on three independent differentiations (n = 3). Significance of enrichment in the SLC10A1-positive population compared with the pre-sorted population was determined by Student's t test, ^∗^p < 0.05.

Immunohistochemistry performed on human liver sections has revealed that SLC10A1, CLRN3, and AADAC are expressed on >75% of the liver's hepatocytes (data provided by the Human Protein Atlas; Uhlen et al., 2010, Uhlén et al., 2015). The broad distribution of these cell-surface proteins throughout the hepatic parenchyma implies that they could facilitate FACS-based purification of a homogeneous population of iPSC-derived cells with hepatocyte characteristics. To test this hypothesis, we used antibodies to SLC10A1, CLRN3, or AADAC and FACS to collect populations of iPSC-derived hepatocytes at day 20 of differentiation and subsequently measured abundance of characteristic hepatic mRNAs in the sorted cells by qRT-PCR. Gating parameters for FACS for SLC10A1-positive iPSC-derived hepatocytes is shown in Figure S5A. As shown in Figure 5, *SLC10A1* mRNA was enriched in SLC10A1-positive cells by ∼3-fold compared with the levels found in pre-sorted cells and was close to undetectable in the SLC10A1-negative population. Analyses of mRNAs encoding CLRN3, AADAC, HNF4A, albumin, ASGR1, APOA1, and AFP by qRT-PCR revealed that each of these hepatocyte markers was significantly enriched in the SLC10A1-positive population compared with both the SLC10A1-negative cells and the pre-sorted cells (Figure 5; Figure S5B). Similar results were obtained when FACS was performed to collect CLRN3- and AADAC-positive cells (Figures S5C and S5D).

**Figure 6:**
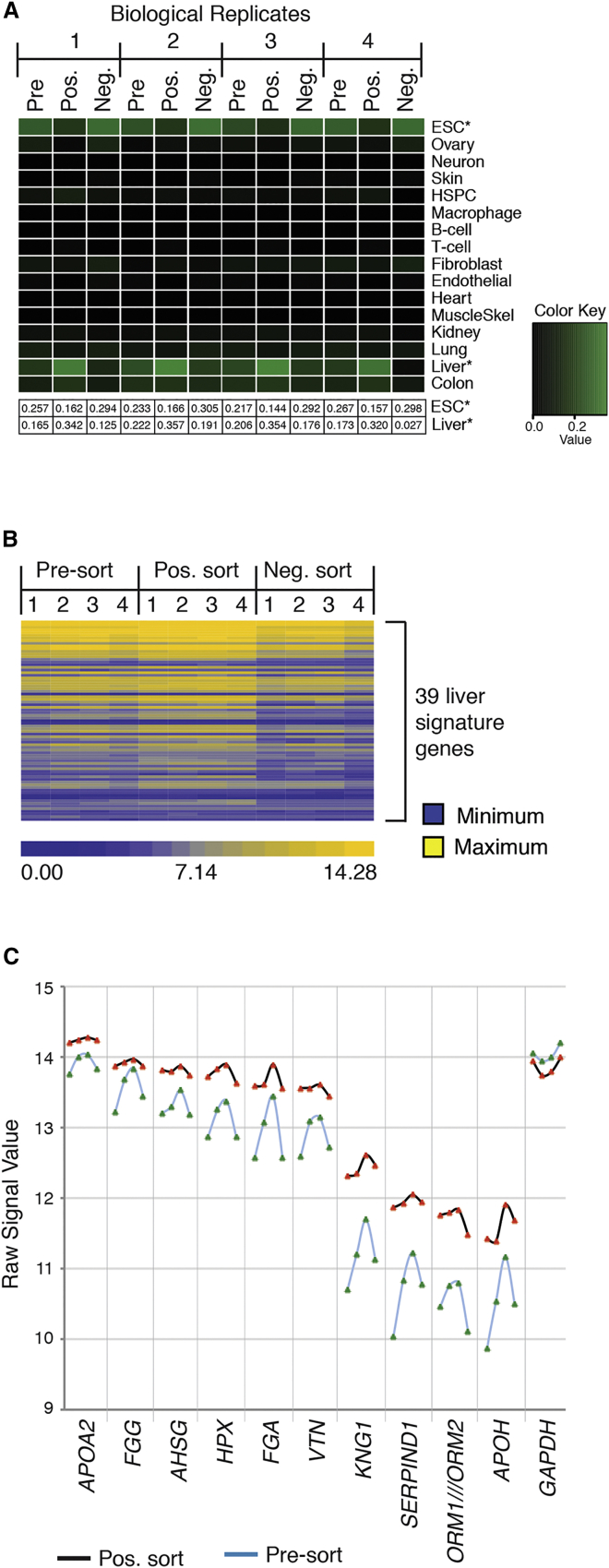
SLC10A1-Positive iPSC-Derived Hepatocyte-like Cells Are Enriched for Hepatic Gene Regulatory Genes and Sorting Them Minimizes Variability Associated with Differentiation (A) Microarray analysis was performed on different cell fractions of iPSC-derived hepatocyte-like cells sorted for expression of SLC10A1. CellNet classification heatmap showing enrichment of liver-specific GRN genes and depletion of ESC-specific GRN genes in the positive sort cell fraction compared with the pre-sort and negative sort cell fractions. Classification scores corresponding to the ESC- and liver-specific GRN genes from the heatmap are presented below the heatmap. Classification scores for different target tissue GRNs represented in the heatmap are provided in Table S2. Microarray data on the pre-sort, positive sort, and negative sort cell fractions from four independent differentiations were used for CellNet analysis (n = 4) (see also Figure S6). (B) Heatmap showing expression levels of 39 liver signature genes in pre-sort, positive sort, and negative sort cell fractions. Probe sets of 39 liver signature genes and the corresponding raw signal values in different cell fractions are provided in Table S3 in the same order as in the heatmap. Microarray data on the pre-sort, positive sort, and negative sort cell fractions from four independent differentiations were used to generate the heatmap (n = 4). (C) Line diagram showing raw signal values of the top ten abundant transcripts selected from 39 liver signature genes in pre-sort and SLC10A1-positive sort cell fractions of hepatocyte-like cells. Signal values of GAPDH transcript served as internal control. Microarray data from four independent differentiations were used to generate the line diagram (n = 4) (see also Figure S6).

Finally, we examined whether FACS selection of SLC10A1-positive iPSC-derived hepatocytes improved overall hepatic characteristics and homogeneity. Four independent iPSC differentiations were performed until day 20, and the cells were subjected to FACS using an anti-SLC10A1 antibody. Pre-sort, SLC10A1-positive, and SLC10A1-negative populations were then subjected to transcriptome analyses using oligonucleotide arrays. Cell profiles were compared using CellNet (Cahan et al., 2014, Morris et al., 2014), which is a computational platform designed to define the similarity between stem-cell-derived cell types and their endogenous counterparts by comparing the expression levels of gene regulatory networks (Figure 6A; Table S2). A higher classification score indicates an increase in the probability that expression of a gene regulatory network resembles that of a target tissue (Cahan et al., 2014, Morris et al., 2014). As expected, in the pre-sorted population, the differentiated cells most closely resembled liver compared with 14 other tissue types. Surprisingly, however, pluripotent cell characteristics were also predominant in the pre-sorted population, suggesting that the cultures contained cells that had failed to differentiate or had retained expression of PSC markers (Figure 6A). When the character of the SLC10A1-expressing cell population was examined, it revealed that the cells had substantially increased hepatic characteristics and reduced pluripotent cell characteristics. In contrast to the SLC10A-positive population, the SLC10A1-negative population lost hepatic characteristics and retained PSC characteristics. We confirmed that the SLC10A1-positive cells more closely resembled hepatocytes compared with the unsorted and SLC10A1-negative cells by examining the levels of mRNAs encoding 39 proteins that are highly enriched in liver tissue (Ge et al., 2005, Si-Tayeb et al., 2010a). These hepatic mRNAs were consistently enriched in the SLC10A1-positive cell population compared with unsorted and negative cell populations (Figure 6B; Table S3).

Although hepatocyte-like cells generated from independent differentiations looked morphologically indistinguishable (Figure S6A), we observed substantial variation in expression of hepatic markers among samples, confirming heterogeneity in the extent of maturation (Figures 6B and 6C; Table S3). However, in contrast to unsorted cells, the SLC10A1-positive population shows uniform levels of hepatic mRNAs among independent differentiations (Figures 6B and 6C; Table S3). These data demonstrate that sorting hepatocyte-like cells using an anti-SLC10A1 antibody facilitates the efficient purification of a homogeneous population of cells with hepatocyte characteristics.

## Discussion

Hepatocyte-like cells generated from iPSCs can be used to model liver development and disease, for toxicity studies, and potentially could offer a source of hepatocytes for cell therapy (Rashid et al., 2010, Delaforest et al., 2011, Cayo et al., 2012, Fox et al., 2014). While the promise of such cells is exciting, improvements to the existing differentiation protocols are necessary to obtain a homogeneous and mature population of hepatocytes whose function recapitulates the native parenchymal cells of the liver. In the current study, we addressed the issue of heterogeneity associated with the differentiation procedure by identifying cell-surface markers that facilitate the purification of homogeneous cell populations.

Traditionally, identification and characterization of cell-surface proteins heavily relied on the availability of antibodies. In such cases, the need for well-characterized antibodies invariably biases study design to focus on CD antigens that are defined by their interaction with monoclonal antibodies (Lanier et al., 1983, Spangrude et al., 1988). Such focus, while not without value, limits the potential repertoire of target proteins that could be used for cell sorting. In contrast, mass spectrometry offers the advantage of being able to identify, characterize, and quantify cell-surface proteins independently of the availability of suitable antibodies. Specifically in the case of the CSC-Technology, the approach exploits the fact that the majority of cell-surface proteins are glycosylated to enable specific detection of transmembrane, GPI-anchored, and extracellular matrix proteins. Moreover, as secreted proteins are also commonly modified by N-linked glycosylation, the CSC-Technology can also detect proteins that are transiting through the secretory pathway, especially if they are abundant. This explains why 71 of 300 proteins identified in our study are annotated as secreted by DAVID. Examples include Apolipoprotein B-100, α-1-antitrypsin, and Coagulation factor V, all of which are abundant serum factors that are known to be secreted by hepatocytes. Although the focus of the current study was to identify proteins that can be used to sort hepatocytes that are derived from iPSCs, the availability of an extensive catalog of proteins present on the hepatocyte cell surface should also be valuable for the study of hepatocyte biology and liver disease. The biological roles of many of the identified proteins within the hepatocyte are poorly understood. This includes one of our target proteins Clarin 3, which is a multi-pass transmembrane protein that has restricted cell distribution among the cell types within the Atlas. While the function of this protein is poorly understood, mutations in a paralog, Clarin 1, have been associated with Usher syndrome, which causes loss of vision and hearing (Joensuu et al., 2001). In addition to providing tools to advance our understanding of liver cell biology, the expanding database of hepatocyte cell-surface proteins should facilitate the identification of receptors involved in infectious disease. In this regard, SLC10A1 (also known as NTCP) has recently been described as a receptor for the hepatitis B and D viruses (Yan et al., 2012). It is, therefore, likely that receptors for known and emerging hepatotropic infectious agents will be present within the catalog of hepatocyte cell-surface proteins we have described.

Previous studies investigating the N-glycoproteome in human liver/hepatocytes are limited. Several proteomic studies have focused on the identification of N-glycoproteins in liver tissue extracts (Sun et al., 2014, Chen et al., 2009, Zhu et al., 2014). Although these approaches provide important information regarding the repertoire of proteins present in the liver, they were not designed to specifically identify cell-surface proteins. Importantly, using whole-liver tissue extract limits the specificity of markers due to the presence of a mixed cell population in the sample. In contrast, Ducret et al. (2015) used a cell-surface capture approach to identify 147 cell-surface N-glycoproteins in primary human hepatocytes. Of the 300 proteins identified in our analyses, Ducret et al. also identified 114 and 186 are unique to our study. Differences in technical approach, including the number of hepatocytes analyzed, likely explain the differences between these two studies. Of note, CLRN3, KLB, SLC22A7, AADAC, SLC10A1, and UGT2B4 were not described as liver-enriched proteins by Ducret et al., which would imply that this screen was not saturated.

The ability to use cell-surface proteins to specifically sort iPSC-derived hepatocyte-like cells is currently limited. The liver-specific cell-surface protein ASGR1 has been used to sort human ESC- and iPSC-derived hepatocyte-like cells (Basma et al., 2009, Peters et al., 2016). These studies demonstrated enrichment of hepatic phenotype in ASGR1-positive cells by performing transcriptional analysis and functional assays. Although we identified ASGR1 on the cell surface of primary hepatocytes, its transcript expression pattern during hPSC differentiation into hepatocyte-like cells did not meet the stringent enrichment criteria we required to select the final candidate proteins for our studies. More recently, microRNA switches have been used to enrich for a desired population of hepatocyte-like cells generated by directed differentiation (Miki et al., 2015). In this approach, reporter mRNAs containing target sequences for miRNAs expressed in the desired cell type are transfected, and diminished reporter expression enables sorting of the target cell population. Although useful, transfection adds technical variability, and differences in transfection efficiency of reporter mRNAs could contribute to differences in the cell types sorted from distinct biological samples. With this in mind, we believe the use of endogenous proteins that are accessible to affinity reagents for live cell sorting will prove most apt for the selection of homogeneous cell populations suitable for research and clinical applications.

Finally, we provide evidence that cell sorting using antibodies to either SLC10A1, CLRN3, or AADAC can improve the quality of hepatocyte-like cells generated from iPSCs. Cell populations resulting from positive selection based on these markers displayed higher levels of characteristic hepatic mRNAs. Importantly, a comparison of marker expression among four independent differentiations revealed that the cells sorted based on the presence of SLC10A1 had substantially lower variation in expression of characteristic mRNAs than the unsorted population. This implies that sorting cells using SLC10A1, CLRN3, or AADAC will improve comparisons among control and patient iPSC-derived hepatocytes. Minimizing heterogeneity within cultures of iPSC-derived cells should facilitate our ability to compare and interpret data resulting from hepatocytes generated from iPSCs that model metabolic liver disease. Until now, because of variations with differentiation, analyses of disease phenotypes have been limited to patients with Mendelian disorders. However, the ability to generate homogeneous populations of iPSC-derived hepatocytes should facilitate analyses of complex phenotypes. For example, several common allelic variations that associate with lipid traits have been identified by genome-wide association studies (Teslovich et al., 2010). Producing homogeneous populations of iPSC-derived hepatocytes from either patient iPSCs or iPSCs modified using genome editing approaches could facilitate direct testing of a specific variant's contribution to levels of secreted lipid.

Use of recombinant laminins as substrates have recently been shown to improve cell organization, cell function, and phenotype of human ESC-derived hepatocyte-like cells (Cameron et al., 2015). Using data published by Cameron et al. (2015), we compared the liver-enriched gene regulatory network (GRN) of SLC10A1-positive cells with hepatocyte-like cells differentiated on recombinant laminin substrates. The GRN of SLC10A1-positive cells appeared to be comparable with hepatocyte-like cells differentiated on recombinant laminin substrates, although we acknowledge that differences in experimental protocol may introduce some error into this comparative analysis (Figure S6B). Importantly, however, SLC10A1-positive cells from independent differentiations exhibited less variation in the liver GRN compared with independent differentiations of hepatocyte-like cells on recombinant laminin substrates, which is consistent with the belief that sorting using such cell-surface markers can enhance the homogeneity of the hepatocyte-like cell population (Figure S6B). These analyses also suggest that by combining approaches, further advances in the quality and homogeneity of iPSC-derived hepatocytes when compared with fresh hepatocytes could be obtained (Figure S6C).

In conclusion, our study provides an expanded dataset of cell-surface N-glycoproteins on primary hepatocytes. We expect this resource, in conjunction with the bioinformatics workflow that includes the evolving Atlas as a platform to identify cell-type-restricted proteins, will be valuable for researchers investigating liver biology and pathophysiology. Importantly, we establish the functional utility of this resource and workflow by identifying three cell-surface N-glycoproteins capable of selecting a homogeneous subpopulation of iPSC-derived hepatocyte-like cells that display enhanced hepatic characteristics.

## Experimental Procedures

### Cell Culture

Procedures for the culture of human iPSCs and their differentiation into hepatocyte-like cells have been described in detail previously (Mallanna and Duncan, 2013). The use of human cells was approved by the Medical College of Wisconsin Stem Cell Research Oversight and Institutional Safety Board committees.

### CSC-Technology

Approximately 100 million primary human hepatocytes were subjected to CSC-Technology workflow as reported previously (Wollscheid et al., 2009, Gundry et al., 2012). Frozen primary human hepatocytes (Hu8176, Hu1443, Hu1460, and Hu1736) were obtained from Life Technologies/Thermo Fisher Scientific. Additional details are provided in Supplemental Experimental Procedures.

### qRT-PCR Analysis

RNA was isolated from iPSC-derived hepatocyte-like cells and primary human hepatocytes using the RNeasy mini or micro kits (QIAGEN). Genomic DNA was removed using 1 μl of RNase-free DNaseI per 5 μg of RNA. First-strand cDNA was synthesized using Moloney murine leukemia virus reverse transcriptase with dNTPs and random hexamer primers. qRT-PCR was performed using either StepOne Plus (Applied Biosystems/Thermo Fisher Scientific) or CFX-384 real-time PCR machine (Bio-Rad) with PrimeTime assays (Table S4) (Integrated DNA Technologies) following the manufacturer's protocols.

### Immunostaining

For immunostaining cell-surface proteins (SLC10A1, CLRN3, AADAC, and ASGR1), fixed, non-permeabilized cells were incubated with antibodies against cell-surface proteins. For co-immunostaining for HNF4A, fixed non-permeabilized cells were incubated with antibodies against cell-surface proteins first, followed by fixing again, permeabilization, and incubation with HNF4A antibody. Finally, cells were incubated with appropriate secondary antibodies and nuclear stain DAPI to complete the staining. Additional details of the immunostaining protocol along with relevant antibody information are provided in the Supplemental Experimental Procedures.

### Fluorescent-Activated Cell Sorting

A subpopulation of iPSC-derived hepatocyte-like cells expressing SLC10A1, CLRN3, and AADAC were sorted using the fluorescent-activated cell sorting (FACS) protocol described in detail in the Supplemental Experimental Procedures. Primary antibodies used were SLC10A1 (Aviva, ARP42097_P050), CLRN3 (Thermo Fisher Scientific, PA5-26137), and AADAC (LSBio, LS-C155827). Mouse monoclonal antibodies (B6E11 and C3B6) were generated commercially (Biomatik) to detect an external epitope on human SLC10A1 (YSRGIYDGDLKDKVPY). Hybridomas and the corresponding monoclonal antibodies have been made available to investigators through the Developmental Studies Hybridoma Bank created by the NICHD and maintained at the University of Iowa (http://dshb.biology.uiowa.edu/Welcome?search=slc10A1).

### Oligonucleotide Array Analysis

Total RNA from different FACS-sorted fractions of iPSC-derived hepatocyte-like cells (pre-sort, SLC10A1 positive sort, SLC10A1 negative sort) from four independent differentiations was isolated using an RNeasy plus micro kit (QIAGEN). Biotinylated cRNA was generated from ∼500 ng of total RNA using the 3′ IVT Plus kit and hybridized to GeneChip Human Genome U133 Plus 2.0 Arrays (Affymetrix). Images were acquired using a GeneChip Scanner 3000 (Affymetrix), and data analysis was performed using the Partek Genomic Suite Statistical Analysis Software (Partek).

## Author Contributions

S.K.M. and S.A.D. designed the study, analyzed the data, and co-wrote the manuscript; S.A.D. supervised the study; S.K.M. performed all experiments except when stated; M.A.C. performed immunostaining and flow cytometry using mouse monoclonal antibodies against SLC10A1; K.T. assisted with microarray experiments; R.L.G. supervised and assisted with CSC-Technology experiments and mass spectrometry data acquisition and analysis. All co-authors approved the final manuscript.

## Figures and Tables

**Table 1:** 40 Cell Surface N-Glycoproteins Identified as Hepatocyte-Restricted Compared with 49 Other Human Cell Types

Gene Symbol	Protein Name	Type
AADAC	arylacetamide deacetylase	TM
ABCA6	ATP-binding cassette sub-family A member 6	TM
ABCB11	bile salt export pump	TM
ABCC6	multidrug resistance-associated protein 6	TM
ABCG8	ATP-binding cassette sub-family G member 8	TM
ANO1	anoctamin-1	TM
AQP9	aquaporin-9	TM
ART4	ecto-ADP-ribosyltransferase 4	GPI
ASGR1	asialoglycoprotein receptor 1	TM
ASGR2	asialoglycoprotein receptor 2	TM
C8A	complement component C8 alpha chain	TM
CDHR5	cadherin-related family member 5	TM
CLRN3	Clarin-3	TM
FAM151A	protein FAM151A	TM
FOLH1	glutamate carboxypeptidase 2	TM
HFE2	hemojuvelin	GPI
KLB	β-klotho	TM
NPC1L1	Niemann-Pick C1-like protein 1	TM
SLC10A1	sodium/bile acid cotransporter	TM
SLC1A2	excitatory amino acid transporter 2	TM
SLC22A1	solute carrier family 22 member 1	TM
SLC22A3	solute carrier family 22 member 3	TM
SLC22A7	solute carrier family 22 member 7	TM
SLC22A9	solute carrier family 22 member 9	TM
SLC2A8	solute carrier family 2, facilitated glucose transporter member 8	TM
SLC39A5	zinc transporter ZIP5	TM
SLC46A3	solute carrier family 46 member 3	TM
SLC6A12	sodium- and chloride-dependent betaine transporter	TM
SLCO1B1	solute carrier organic anion transporter family member 1B1	TM
SLCO1B3	solute carrier organic anion transporter family member 1B3	TM
TFR2	transferrin receptor protein 2	TM
TM4SF4	transmembrane 4 L6 family member 4	TM
TM4SF5	transmembrane 4 L6 family member 5	TM
TMEM110	store-operated calcium entry regulator STIMATE	TM
TMPRSS6	transmembrane protease serine 6	TM
TMUB1	transmembrane and ubiquitin-like domain-containing protein 1	TM
UGT1A4	UDP-glucuronosyltransferase 1-4	TM
UGT2B15	UDP-glucuronosyltransferase 2B15	TM
UGT2B4	UDP-glucuronosyltransferase 2B4	TM
UGT2B7	UDP-glucuronosyltransferase 2B7	TM

TM, transmembrane; GPI, glycophosphatidylinositol.

## Accession Numbers

All original gene array CEL files are available from the Gene Expression Omnibus (GEO) accession number GEO: GSE80279.

## Supplemental Information

Document S1. Supplemental Experimental Procedures, Figures S1–S6, and Table S4

Table S1. 300 Cell-Surface N-Glycoproteins Identified by the CSC-Technology in Primary Human Hepatocytes, Related to Figure 2

Table S2. CellNet Analysis of SLC10A1 Sorted iPSC-Derived Hepatocyte-like Cells, Related to Figure 6A

Table S3. Enrichment of Liver Signature Genes in SLC10A1 Sorted iPSC-Derived Hepatocyte-like Cells, Related to Figures 6B and 6C

Document S2. Article plus Supplemental Information
