# Supplementary material for: Mapping the Cell-Surface N-Glycoproteome of Human Hepatocytes Reveals Markers for Selecting a Homogeneous Population of iPSC-Derived Hepatocytes
Source: Stem Cell Reports. 2016 Aug 25;7(3):543–56. doi: 10.1016/j.stemcr.2016.07.016 (PMC5032032; doi:10.1016/j.stemcr.2016.07.016)
Supplement: Document S1. Supplemental Experimental Procedures, Figures S1–S6, and Table S4 [file mmc1.pdf]

Stem Cell Reports, Volume 7

Supplemental Information

Mapping the Cell-Surface N-Glycoproteome of Human Hepatocytes Reveals Markers for Selecting a Homogeneous Population of iPSC-Derived Hepatocytes

Sunil K. Mallanna, Max A. Cayo, Kirk Twaroski, Rebekah L. Gundry, and Stephen A. Duncan

Figure S1, related to Figure 2. Bar graph showing results of DAVID analysis of 300 proteins identified in primary hepatocytes by cell surface capture. Bar graphs showing a representation of the top 10 proteins categorized using SwissProt keywords **(A)**, and gene ontology molecular function terms **(B)** that were enriched in the cell surface capture (CSC) data compared to gene distribution in the human genome (Y-axis). Numbers in parenthesis represent the number of proteins out of a total of 300 identified by CSC that fall under the indicated annotation. Enrichment of each classification is expressed as a log10 p value on the X-axis.

A

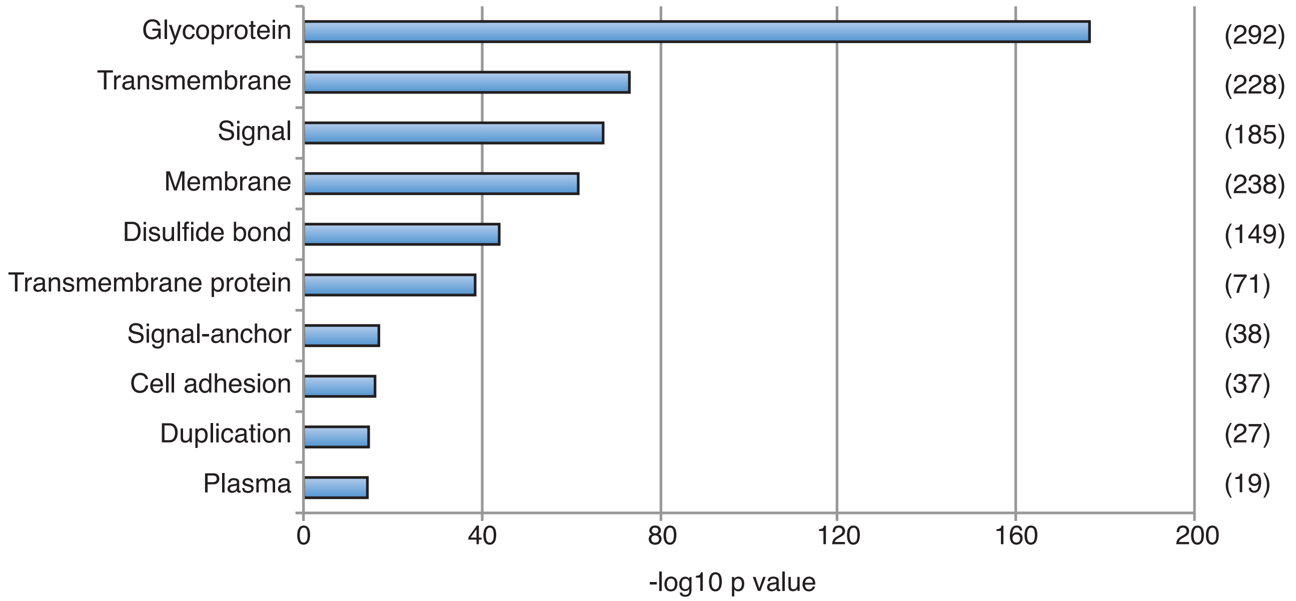

B

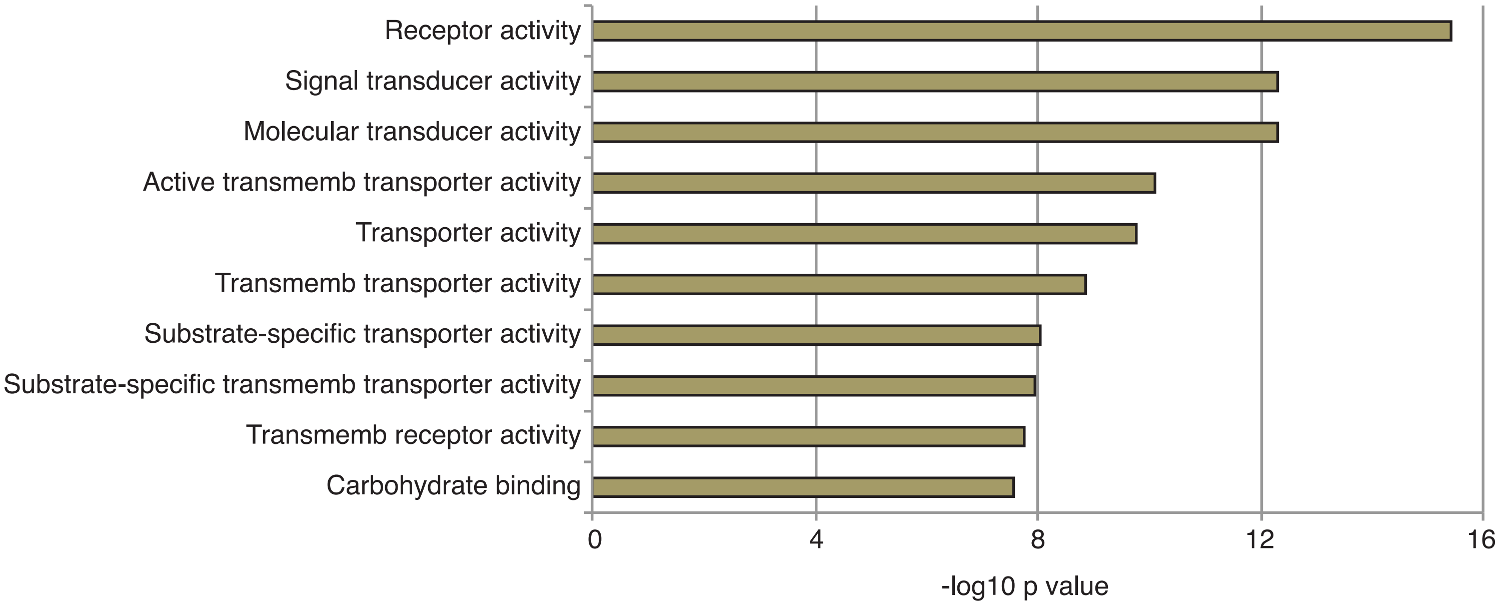

Figure S2, related to Figure 2 and 3. Representative annotated MS/MS Spectra for three candidate proteins.

SLC10A1

Sequence: MEAHNASAPFNFTLPPNFGK, N5-Deamidated (0.98402 Da), N11-Deamidated (0.98402 Da), M1-Oxidation (15.99492 Da)
Charge: +3, Monoisotopic m/z: 736.67346 Da (-0.27 mmu/-0.36 ppm), MH+: 2208.00583 Da, RT: 57.5920 min,
Identified with: Sequest HT (v1.17); XCorr:3.24, Percolator q-Value:0, Percolator PEP:0.0109, Ions matched by search engine: 0/0
Fragment match tolerance used for search: 0.6 Da

Fragment Matches

Value Type: Theo. Mass [Da]

Ion Series | Neutral Losses | Precursor Ions

#1	b^+	b^{2+}	b^{3+}	Seq.	y^+	y^{2+}	y^{3+}	#2
1	148.04268	74.52498	50.01908	M-Oxidation				20
2	277.08527	139.04627	93.03327	E	2060.97123	1030.98925	687.66193	19
3	348.12238	174.56483	116.71231	A	1931.92864	966.46796	644.64773	18
4	485.18129	243.09429	162.39862	H	1860.89153	930.94940	620.96869	17
5	600.20824	300.60776	200.74093	N-Deamid...	1723.83261	862.41994	575.28239	16
6	671.24535	336.12631	224.41997	A	1608.80567	804.90647	536.94007	15
7	758.27738	379.64233	253.43064	S	1537.76856	769.38792	513.26104	14
8	829.31449	415.16089	277.10968	A	1450.73653	725.87190	484.25036	13
9	926.36726	463.68727	309.46060	P	1379.69941	690.35335	460.57132	12
10	1073.43567	537.22147	358.48341	F	1282.64665	641.82696	428.22040	11
11	1188.46262	594.73495	396.82572	N-Deamid...	1135.57824	568.29276	379.19760	10
12	1335.53103	668.26915	445.84853	F	1020.55129	510.77928	340.85528	9
13	1436.57871	718.79299	479.53109	T	873.48288	437.24508	291.83248	8
14	1549.66277	775.33502	517.22577	L	772.43520	386.72124	258.14992	7
15	1646.71554	823.86141	549.57670	P	659.35114	330.17921	220.45523	6
16	1743.76830	872.38779	581.92762	P	562.29837	281.65282	188.10431	5

Fragment Spectrum

OT2_Gundry_2013-01-22_SM42-03.raw #8767 RT: 57.5920 min
ITMS, 737.0066@cid35.00, z=+3, Mono m/z=736.67346 Da, MH+=2208.00583 Da, Match Tol.=0.8 Da

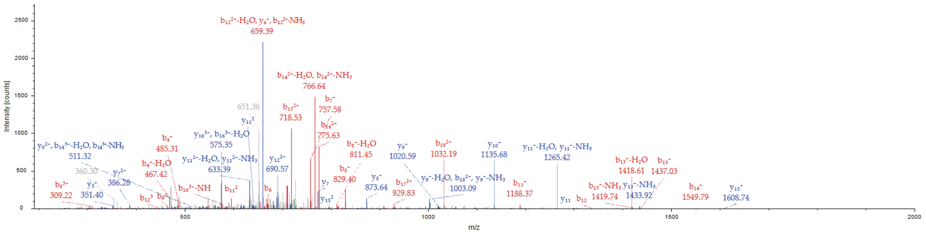

CLRN3

Sequence: DSASNGSIFITY, N5-Deamidated (0.98402 Da)
Charge: +2, Monoisotopic m/z: 638.29095 Da (+0.35 mmu/+0.54 ppm), MH+: 1275.57463 Da, RT: 47.9004 min,
Identified with: Sequest HT (v1.17); XCorr:3.31, Percolator q-Value:0, Percolator PEP:0.000377, Ions matched by search engine: 0/0
Fragment match tolerance used for search: 0.6 Da

Fragment Matches

Value Type: Theo. Mass [Da]

Ion Series | Neutral Losses | Precursor Ions

#1	b^+	b^{2+}	Seq.	y^+	y^{2+}	#2
1	116.03422	58.52075	D			12
2	203.06625	102.03676	S	1160.54700	580.77714	11
3	274.10336	137.55532	A	1073.51497	537.26112	10
4	361.13539	181.07133	S	1002.47785	501.74257	9
5	476.16233	238.58481	N-Deamid...	915.44583	458.22655	8
6	533.18380	267.09554	G	800.41888	400.71308	7
7	620.21583	310.61155	S	743.39742	372.20235	6
8	733.29989	367.15358	I	656.36539	328.68633	5
9	880.36830	440.68779	F	543.28133	272.14430	4
10	993.45237	497.22982	I	396.21291	198.61009	3
11	1094.50005	547.75366	T	283.12885	142.06806	2
12			Y	182.08117	91.54422	1

Fragment Spectrum

OT2_Gundry_2013-01-22_SM42-03.raw #7325 RT: 47.9004 min
ITMS, 638.2898@cid35.00, z=+2, Mono m/z=638.29095 Da, MH+=1275.57463 Da, Match Tol.=0.8 Da

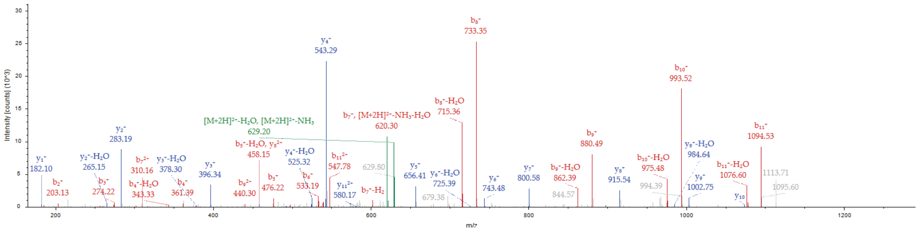

AADAC

Sequence: VVGSFDEVPPTSDENVTVTETK, N15-Deamidated (0.98402 Da)
Charge: +2, Monoisotopic m/z: 1176.06055 Da (+0.08 mmu/+0.07 ppm), MH+: 2351.11382 Da, RT: 38.7348 min,
Identified with: Sequest HT (v1.17); XCorr:5.25, Percolator q-Value:0, Percolator PEP:0.000209, Ions matched by search engine: 0/0
Fragment match tolerance used for search: 0.6 Da

Fragment Matches

Value Type: Theo. Mass [Da]

Ion Series | Neutral Losses | Precursor Ions

#1	b^+	b^{2+}	Seq.	y^+	y^{2+}	#2
1	100.07569	50.54148	V			22
2	199.14410	100.07569	V	2252.04524	1126.52626	21
3	256.16557	128.58642	G	2152.97683	1076.99205	20
4	343.19760	172.10244	S	2095.95537	1048.48132	19
5	490.26601	245.63664	F	2008.92334	1004.96531	18
6	605.29295	303.15011	D	1861.85492	931.43110	17
7	734.33555	367.67141	E	1746.82798	873.91763	16
8	833.40396	417.20562	V	1617.78539	809.39633	15
9	930.45672	465.73200	P	1518.71697	759.86213	14
10	1027.50949	514.25838	P	1421.66421	711.33574	13
11	1128.55717	564.78222	T	1324.61145	662.80936	12
12	1215.58919	608.29824	S	1223.56377	612.28552	11
13	1330.61614	665.81171	D	1136.53174	568.76951	10
14	1459.65873	730.33300	E	1021.50480	511.25604	9
15	1574.68567	787.84648	N-Deamid...	892.46220	446.73474	8
16	1673.75409	837.38068	V	777.43526	389.22127	7

Fragment Spectrum

OT2_Gundry_2013-01-22_SM42-01.raw #5712 RT: 38.7348 min
ITMS, 1176.5618@cid35.00, z=+2, Mono m/z=1176.06055 Da, MH+=2351.11382 Da, Match Tol.=0.8 Da

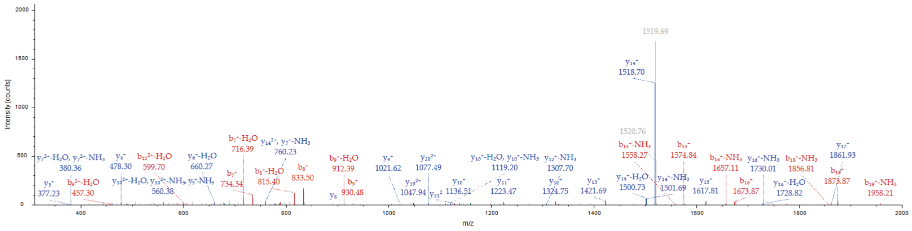

Figure S3, related to Figure 3 and Figure 4. Expression of SLC10A1, CLRN3, and AADAC in hepatocyte-like cells
(A) SLC10A1, CLRN3, and AADAC mRNAs are expressed at lower levels in human ES cell derived hepatocyte-like cells compared to primary hepatocytes. Bar graphs of qRT-PCR results showing levels of mRNAs encoding cell surface proteins SLC10A1, CLRN3, and AADAC in H1 and H9 human ES Cell derived hepatocyte-like cells in comparison to primary hepatocytes. ASGR1 and Albumin mRNA levels are included as reference points. Data from H1 and H9 human ES cell derived hepatocyte-like cells are from a single differentiation. Data from primary hepatocytes is presented as a mean of 3 independent samples.
(B and C) A subpopulation of iPSC-derived hepatocyte-like cells co-express SLC10A1, CLRN3, and AADAC. Micrographs showing the results of immunocytochemistry to detect co-staining of cell surface proteins SLC10A1 (clone C3B6 mouse monoclonal, red), CLRN3 (rabbit polyclonal, green), and AADAC (rabbit polyclonal, green) in K3 iPSC-derived hepatocyte-like cells (B), and SV20 iPSC-derived hepatocyte-like cells (C). Nuclei are identified by DAPI staining. Scale bars = 100 um.

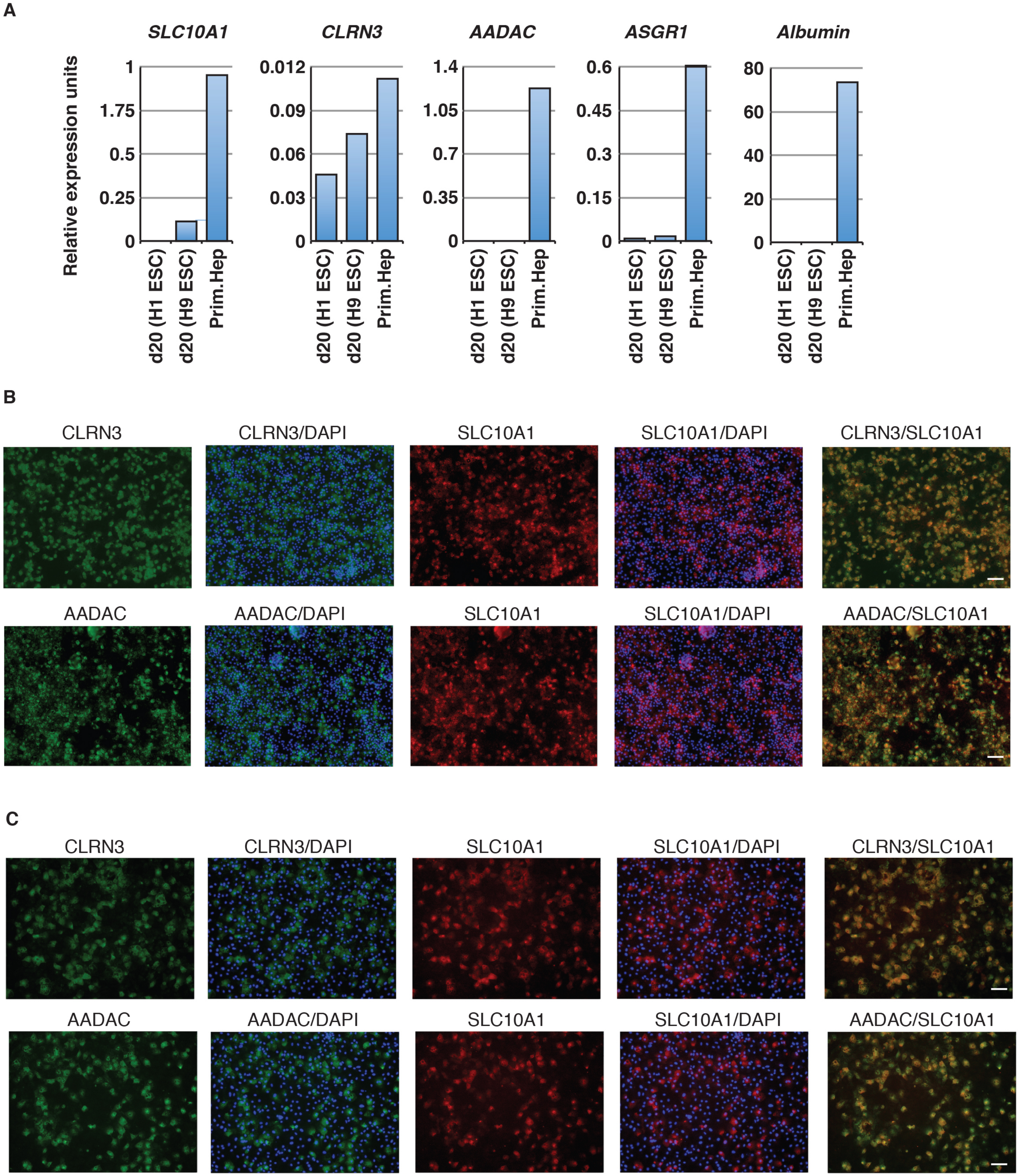

Figure S4, related to Figure 4. ASGR1 is expressed in a subpopulation of iPSC-derived hepatocyte-like cells that co-express SLC10A1, CLRN3, and AADAC. Confocal micrographs showing the results of immunocytochemistry to detect co-staining of ASGR1 (mouse monoclonal, green), with SLC10A1 (rabbit polyclonal, red), CLRN3 (rabbit polyclonal, red), and AADAC (rabbit polyclonal, red) in K3 iPSC-derived hepatocyte-like cells. Nuclei are identified by DAPI staining (blue). White arrows point to representative cells that stained positive with only ASGR1 suggesting less-restrictive expression pattern of ASGR1 in hepatocyte-like cells compared to SLC10A1, CLRN3, and AADAC. Scale bars = 100 um.

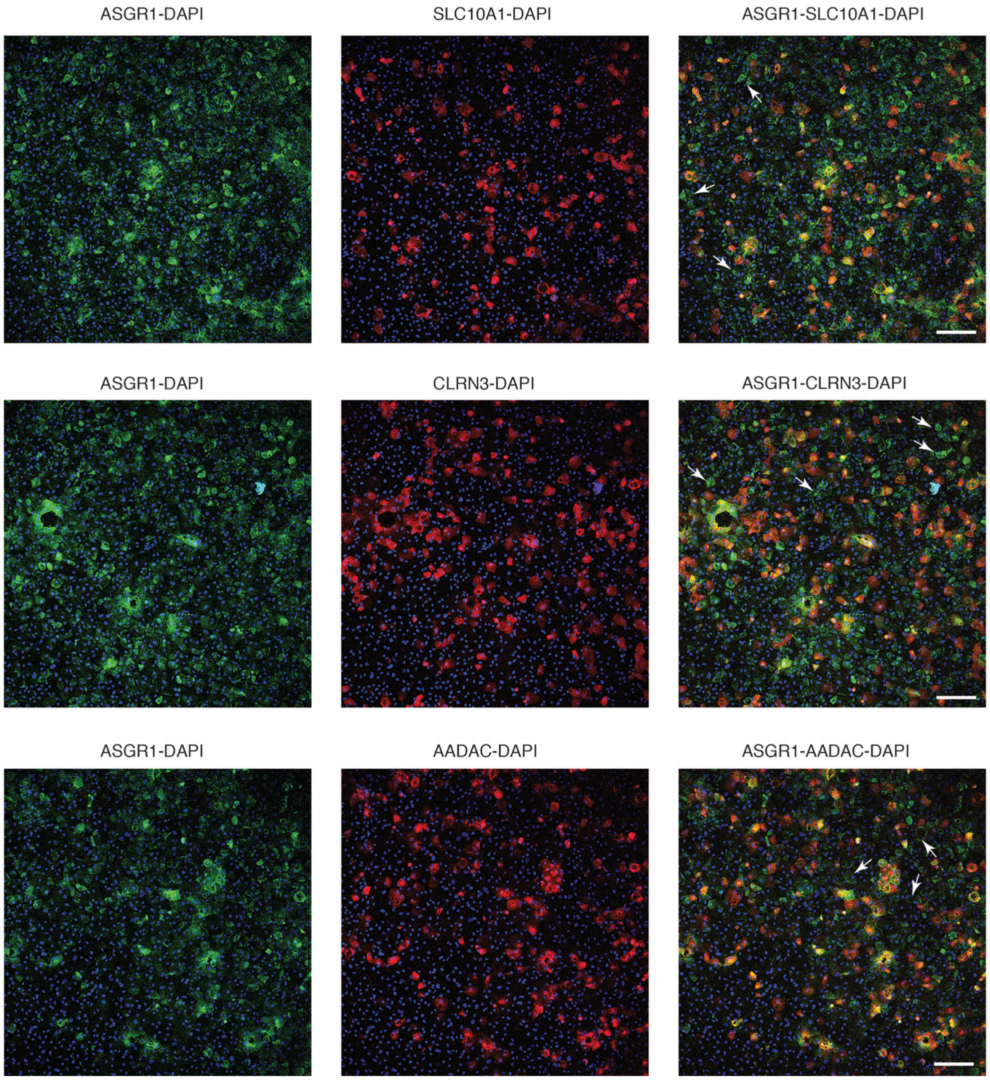

Figure S5, related to Figure 4 and 5. A subpopulation of iPSC-derived hepatocyte-like cells sorted by FACS using antibodies that recognize SLC10A1, CLRN3, or AADAC express several hepatic mRNAs. (A) Dot plot showing gating parameters for FACS sorting SLC10A1 positive and negative iPSC-derived hepatocyte-like cell population. Dead cells are excluded out by staining with DAPI and live cells within the compartment P1 are used for sorting positive and negative cell fractions. No antibody control and PE-conjugated secondary antibody control are used to set up gating parameters for collecting negative cell fraction. iPSC-derived hepatocyte-like cells stained with SLC10A1 antibody and corresponding PE-conjugated secondary antibody is used to set up gating parameters for collecting SLC10A1 positive cell fraction. **(B)** Data in Figure 5 is presented here to demonstrate relative enrichment of selected mRNAs in SLC10A1-positive cell fraction compared to SLC10A1-negative cell fraction which is set to 1. Data is presented as mean and SEM from sorting performed on 3 independent differentiations. **(C and D)** Bar graphs show qRT-PCR analyses to determine relative levels of selected mRNAs in pre-sort, positive sort, and negative sort cell fractons from CLRN3 (C) and AADAC (D) sorting experiments. Data presented is from a single differentiation and sorting experiment.

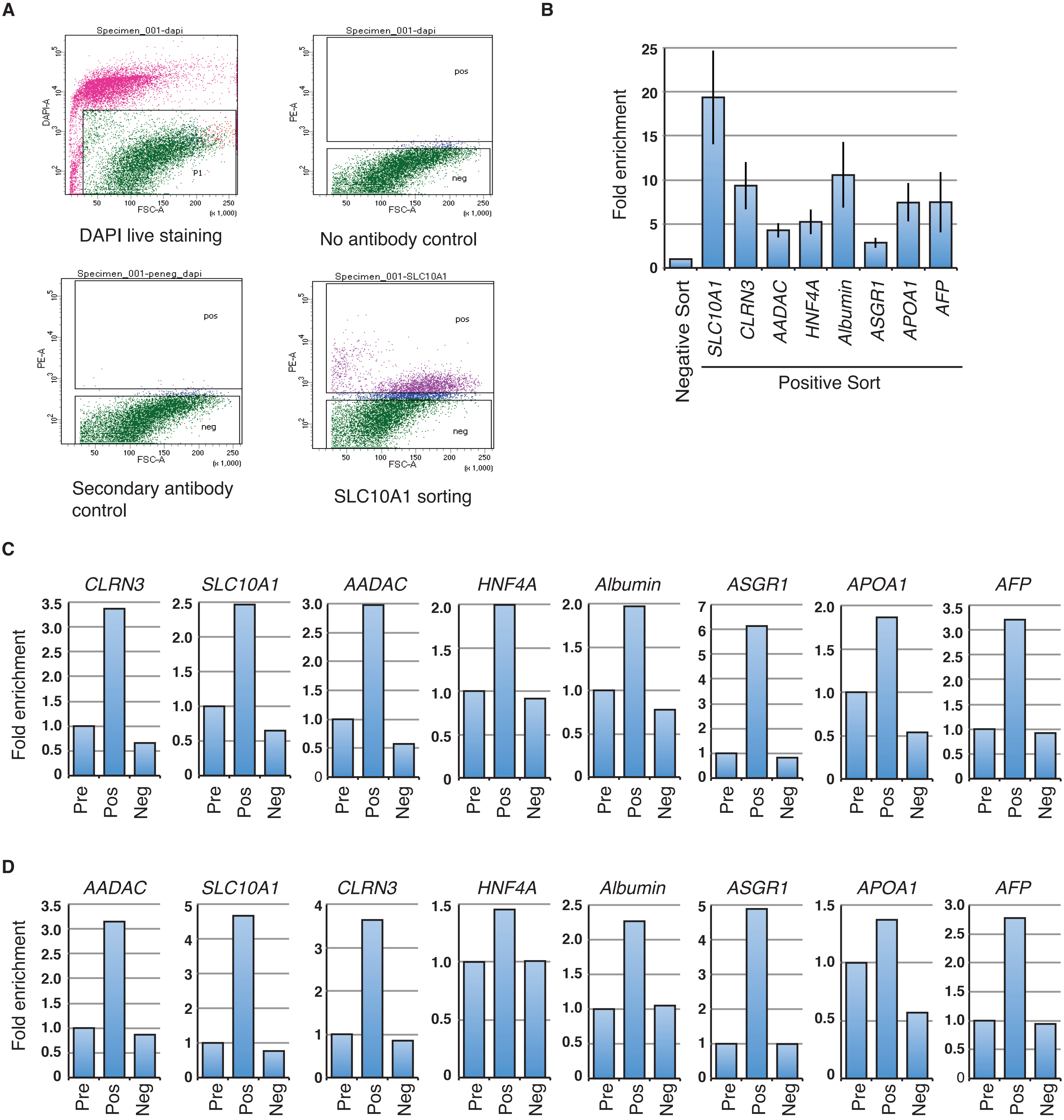

Figure S6, related to Figure 6. CellNet analysis of different fractions of iPSC-derived hepatocyte-like cells FACS sorted using SLC10A1 antibody. (A) Brightfield micrographs showing K3 iPSC-derived hepatocyte-like cells from four independent differentiations used for FACS sorting and microarray analysis in Figure 6. Scale bar = 100 um. **(B)** CellNet analysis to compare GRN of SLC10A1 positive cell fractions of hepatocyte-like cells (n = 4 independent differentiations; pos_1-4) with hepatocyte-like cells differentiated by Cameron et al (Cameron et al., 2015, Stem Cell Reports, 5, 1250-62) on Laminin 111 mix (n = 3 independent differentiations; 1-3) and Laminin 521 (n = 3 independent differentiations; 1-3). Each bar represents data from an independent experiment. **(C)** CellNet analysis to compare GRN of SLC10A1 positive (pos_1-4) and negative cell fractions (neg_1-4) of hepatocyte-like cells from 4 independent differentiations with published array data (Cameron et al., 2015, Stem Cell Reports, 5, 1250-62) from freshly isolated human hepatocytes (n = 3 samples; 1-3). Hepatocyte microarray data were obtained from EMBL-EBI: E-MTAB-3994**.** Each bar represents data from an independent experiment.

A

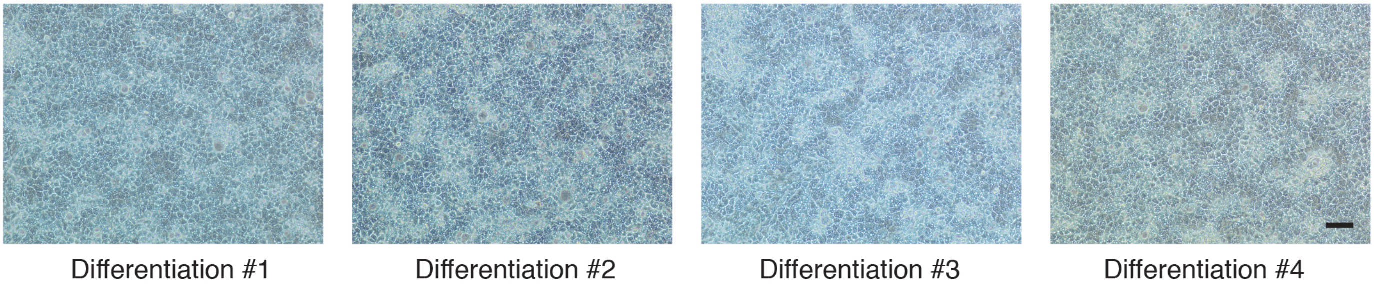

B

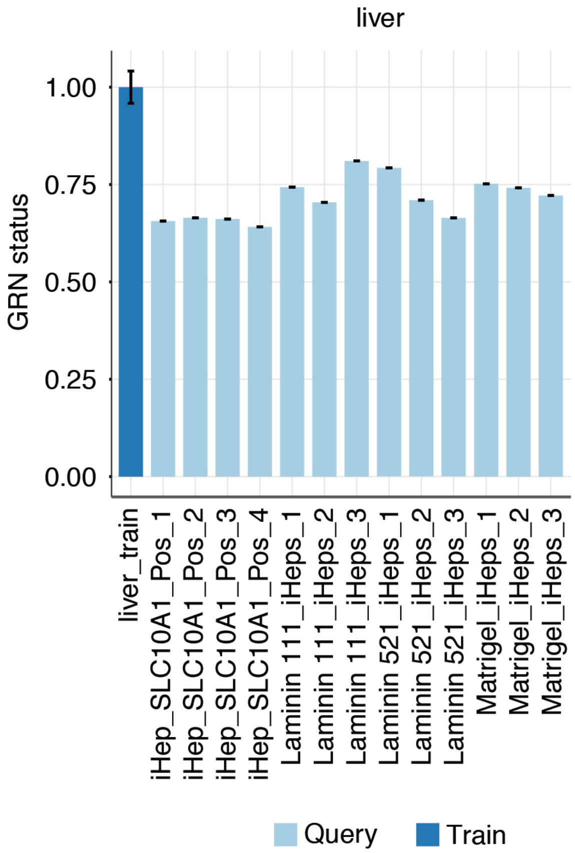

C

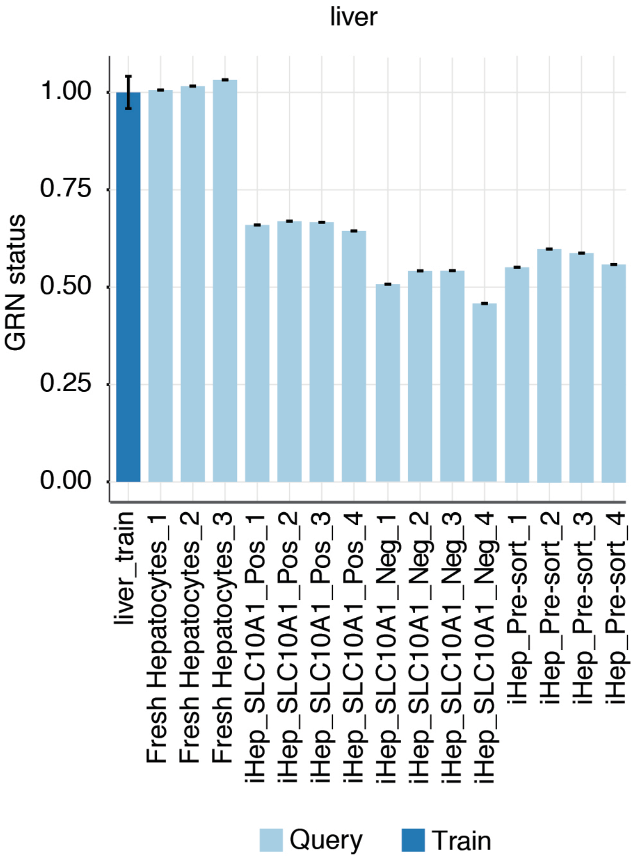

Table S4. List of PrimeTime assays used in qRT-PCR analysis

Gene	Forward	Probe	Reverse
SLC10A1	TGTACAGGAGGAGAGGCATC	AACCTCAGCATTGTGATGACCACCT	ACCTGTCCAATGTCTTCAGTC
CLRN3	GAGCTCTGGGTTTACCTTCTAC	AGCATCAGCAACCCTTACCAGACA	GTGTTCGCCACAAACAGTATC
AADAC	GCCATGGAGAATGATGTGGATA	ACATTTGTGGAGCTCCTGGGACTT	AAAGCTCCCGACAACCTTAAA
HNF4a	TGGACAAAGACAAGAGGAACC	CAAGAAATGCTTCCGGGCTGGC	ATAGCTTGACCTTCGAGTGC
Albumin	AAATCCCACTGCATTGCCGAAGTG	TGCCTGCTGACTTGCCTTCATTAGCT	AGCAGCAGCACGACAGAGTAATCA
ASGR1	TCCTTTCTGAGCCATTGCC	CGTGAAGCAGTTCGTGTCTGACCT	TGAAGTCGCTAGAGTCCCAG
ApoA1	CTTTGAGCACATCCACGTACA	CTGCCAGAAATGCCGAGCCTG	GCCGTGCTCTTCCTGAC
AFP	CTGCAATTGAGAAACCCACTG	TTGGAGAAGTACGGACATTCAGACTGC	TTCCCTCTTCACTTTGGCTG

Supplemental Experimental Procedures

Mass Spectrometry and Data Analysis. Peptides from the CSC-Technology were analyzed over three technical replicate injections on an LTQ Orbitrap Velos as described in PMID: 25068131. MS/MS spectra were processed using Proteome Discoverer 2.2 against the UniProt Human database. SequestHT and MSAmanada search algorithms were used and followed by Percolator (default settings) for post-search processing. Precursor mass tolerance was 10 ppm, fragment mass tolerance was 0.6 Da., and 2 missed cleavages were allowed. Allowed variable modifications were deamidation (N), N-terminal acetylation, and oxidation (M). Carbamidomethylation (C) was a fixed modification. To be considered in the final dataset, proteins had to be deemed "high confidence" based on either SequestHT or MSAmanada, and identified by at least 3 peptide spectrum matches.

Immunostaining. iPS cell-derived hepatocyte-like cells were washed once with 1x PBS, fixed with 4% PFA for 20 mins at room temperature followed by washing with 1x PBS for 3 x 5 mins. For staining cell surface proteins, fixed cells were blocked with 3% BSA in 1x PBS for 60 mins at room temperature followed by incubation with primary antibody against desired cell surface protein overnight at 4°C. For co-staining cell surface proteins with intracellular protein HNF4A, fixed cells were incubated with primary antibody against cell surface protein as described above, fixed again with 4% PFA for 20 mins at room temperature, washed with 1x PBS for 3 x 5 mins. Fixed cells were permeabilized with 0.4% Triton-X for 20 minutes at room temperature and washed with 1x PBS for 3 x 5 mins. Permeabilized cells were blocked with 3% BSA in 1x PBS for 60 mins at room temperature followed by incubation with primary antibody against HNF4A overnight at 4°C. Cells incubated with primary antibody were washed with 1x PBS for 3 x 5 mins, incubated with appropriate secondary antibody for 60 mins at room temperature, washed again with 1x PBS for 3 x 5 mins and imaged. Primary antibodies used were SLC10A1 (Aviva, ARP42097_P050, 1:200; mouse monoclonal, 1:50), CLRN3 (Thermo Fisher Scientific, PA5-26137, 1:200), AADAC (LSBio, LS-C155827, 1:200), ASGR1 (BD Biosciences, 563654, 1:100), and HNF4 (Santa Cruz, sc-6556, 1:250). Secondary antibodies used were A11055 Alexa fluor anti-goat 488 nm, A21207 Alexa fluor anti-rabbit 594 nm, A21206 Alexa fluor anti-rabbit 488 nm, A21203 Alexa fluor anti-mouse 594 nm. Secondary antibodies were used at 1:500 and nuclear stain DAPI (D1306, Thermo Fisher Scientific, 5 mg/ml) was used at 1:5000. Images were processed identically using Adobe Photoshop to optimize brightness and contrast.

FACS sorting iPSC-derived hepatocyte-like cells using SLC10A1 antibody. iPS cell-derived hepatocyte-like cells in 6-well plates were washed once with Versene and incubated in 1 ml of Versene per well at 37°C for 20-25 mins until cell to cell contact looks loose when looking through microscope. Add 1 ml of pre-warmed DMEM to each well, remove Versene-DMEM mixture, and add 1 ml of pre-warmed Collagenase mixture (2 mg/ml Collagenase XI, 1 mg/ml dispase, 100 U/ml DNAse, 0.2% DMSO in 50:50 mixture of HBM and DMEM mixture). Incubate cells in Collagenase mixture at 37°C for ~35-40 mins. During incubation pipette cells 2-3 times using 1 ml pipette to break up clumps to aid dissociation. Incubation with Collagenase mixture should result in cell clumps smaller than 5 cells. Add 500 ul of Versene to each well, collect Collagenase-Versene mixture in a 1.5 ml tube and centrifuge at 400g for 5 mins at RT. Resuspend cell pellet in each 1.5 ml tube in 500-750 ul of Versene, and transfer cell suspension to an suspension culture plate and incubate at 37°C for ~45 mins. During incubation, pipette cells 3 times using 5 ml serological pipette to aid in cell dissociation. Towards the end of Versene incubation, majority of cells should be dissociated to single cells. Filter dissociated cells using 3-4 100 um cell strainers to remove large cell clumps, if any. Collect residual cells struck to the strainer by adding 2 ml of cold 0.5% BSA per strainer. Pool all strained cells and centrifuge at 400g for 5 mins at 4°C. Remove Versene-BSA mixture and block by resuspending in 5-10 ml of 1% BSA, and incubate on ice for ~45 mins. While cells are incubating in blocking mixture, stain cells using trypan blue and determine viability. Aliquot required number of cells for sorting using antibody against SLC10A1 and necessary controls for setting up gating parameters and for collecting gated pre-sort cell fraction (Cell only control, DAPI only control, DAPI + secondary antibody control). Remove blocking buffer by centrifuging at 400g for 5 mins at 4°C. Resuspend cell pellet using 25 ul of 0.25% BSA in 1x PBS per one million cells. To the cells meant to be sorted with SLC10A1 antibody, add 1 ug of SLC10A1 antibody (Aviva, ARP42097_P050) per 1 million cells. Incubate cells at 4°C for 1 hour using an end-to-end rotator. After incubation, wash by resuspending in 1.3 ml of 0.25% BSA in 1x PBS followed by pelleting cells at 400g for 5 mins at 4°C. Resuspend the cell pellet using 25 ul of 0.25% BSA in 1x PBS per 1 million cells and 0.4 ul of donkey anti-rabbit PE (Santa Cruz, sc-3745) per 1 million cells to the sample meant to be sorted with SLC10A1 antibody and DAPI + secondary antibody control sample. Incubate cells at 4°C for 30 mins with flicking tubes every 10 mins to allow proper suspension of cells during incubation. After incubation with secondary antibody, wash cells twice by suspending in 0.25% BSA in 1x PBS followed by pelleting by centrifugation at 400g for 5 mins at 4°C. After washing resuspend the cells in 0.25% BSA in 1x PBS, filter cells using 100 um cell strainers to remove clumps formed by any dead

cells. Finally, add 2 ul of 1:50 diluted 5 mg/ml DAPI stock (D1306, Thermo Fisher Scientific) per 1 million cells to 'DAPI only control', 'DAPI + secondary antibody control', and 'SLC10A1 sort' samples to gate out dead cells. Mixed cells by pipetting gently and cells were sorted using BD FACS Aria IIIu sorter. Cell gating was set to collect live cells from an unstained sample as pre-sort. Dot plot from 'no antibody' and 'secondary antibody only' control samples were used to establish gating parameters for collecting SLC10A1 positive and negative cell fractions. Sorted cells were collected in 1.5 ml tubes containing 100 ul of FBS. Sorted cells were pelleted by centrifugation at 8000 g for 5 mins at 4°C, suspended in RLT plus buffer from RNeasy plus micro kit, flash froze in liquid nitrogen, and stored at -80°C for future use for preparing RNA.

FACS sorting iPSC-derived hepatocyte-like cells using CLRN3 or AADAC antibody. General protocol for FACS sorting iPSC-derived hepatocyte-like cells using CLRN3 or AADAC antibody is similar to sorting protocol described above using SLC10A1 antibody. For sorting CLRN3 positive hepatocyte-like cells, 2 ug of CLRN3 antibody (Thermo Scientific, PA5-26137) per 1 million cells was used. For sorting AADAC positive hepatocyte-like cells, 2 ug of AADAC antibody (LSBio, LS-C155827) per 1 million cells was used. For sorting using CLRN3 or AADAC antibodies, DAPI staining was not done and cell gating was set to collect cells from an unstained sample as pre-sort. Dot plot from 'no antibody' and 'secondary antibody only' control samples were used to establish gating parameters for collecting positive and negative cell fractions.

Statistical Analyses. Student's t-test (two-tailed, unpaired, equal variance) was used when comparing two different conditions/samples and ANOVA-1 way was used when comparing more than two conditions/samples.

Z-score analysis. For identifying transcripts that increased during maturation stage of the differentiation protocol presented in Figure 3A, previously published microarray data from our laboratory on H9 undifferentiated and day 5, 10, 15, and 20 hepatic differentiated cells were used (samples 1 and 2 from GEO database accession numbers GSE14897 and GSE25417). Fold change in transcript levels between days 10 and 15, and days 15 and 20 were calculated by ANOVA-1 way analysis. Z-score value was calculated using mean and standard deviation of fold change for the entire data set. Z-score value of 3 corresponding to 9.27 and 5.06 fold enrichment between days 10 and 15, and days 15 and 20, respectively, was used as cut off.
