# Supplementary material for: Mapping the Cell-Surface N-Glycoproteome of Human Hepatocytes Reveals Markers for Selecting a Homogeneous Population of iPSC-Derived Hepatocytes
Source: Stem Cell Reports. 2016 Aug 25;7(3):543–56. doi: 10.1016/j.stemcr.2016.07.016 (PMC5032032; doi:10.1016/j.stemcr.2016.07.016)
Supplement: Document S2. Article plus Supplemental Information [file mmc5.pdf]

Stem Cell Reports
Resource
OPEN ACCESS

Mapping the Cell-Surface N-Glycoproteome of Human Hepatocytes Reveals Markers for Selecting a Homogeneous Population of iPSC-Derived Hepatocytes

Sunil K. Mallanna,[1] Max A. Cayo,[2] Kirk Twaroski,[2,4] Rebekah L. Gundry,[3] and Stephen A. Duncan[1,*]
[1]Department of Regenerative Medicine and Cell Biology, Medical University of South Carolina, Charleston, SC 29425, USA
[2]Department of Cell Biology, Neurobiology and Anatomy, Program in Regenerative Medicine
[3]Department of Biochemistry
Medical College of Wisconsin, Milwaukee, WI 53226, USA
[4]Department of Pediatrics, University of Minnesota, Minneapolis, MN 55454, USA
*Correspondence: duncanst@musc.edu
http://dx.doi.org/10.1016/j.stemcr.2016.07.016

SUMMARY

When comparing hepatic phenotypes between iPSC-derived hepatocyte-like cells from different liver disease patients, cell heterogeneity can confound interpretation. We proposed that homogeneous cell populations could be generated by fluorescence-activated cell sorting (FACS). Using cell-surface capture proteomics, we identified a total of 300 glycoproteins on hepatocytes. Analyses of the expression profiles during the differentiation of iPSCs revealed that SLC10A1, CLRN3, and AADAC were highly enriched during the final stages of hepatocyte differentiation. FACS purification of hepatocyte-like cells expressing SLC10A1, CLRN3, or AADAC demonstrated enrichment of cells with hepatocyte characteristics. Moreover, transcriptome analyses revealed that cells expressing the liver gene regulatory network were enriched while cells expressing a pluripotent stem cell network were depleted. In conclusion, we report an extensive catalog of cell-surface N-linked glycoproteins expressed in primary hepatocytes and identify cell-surface proteins that facilitate the purification of homogeneous populations of iPSC-derived hepatocyte-like cells.

INTRODUCTION

Directed differentiation of pluripotent stem cells (PSCs) to cells of a specific fate holds promise to study a wide variety of human diseases (Robinton and Daley, 2012). Several groups have reported the generation of hepatocyte-like cells from human PSCs by the sequential addition of growth factors (Agarwal et al., 2008; Basma et al., 2009; Cai et al., 2007; Hay et al., 2008; Song et al., 2009; Si-Tayeb et al., 2010a; Sullivan et al., 2010). The cells produced by these approaches share many characteristics with primary hepatocytes, although transcriptional profiling has suggested that the cells in general tend to be less mature than their native counterparts (Si-Tayeb et al., 2010a). Nevertheless, induced PSCs (iPSCs) derived from patients with inborn errors in hepatic metabolism have been used to successfully model several liver diseases in culture (Rashid et al., 2010; Cayo et al., 2012; Choi et al., 2013; Tafaleng et al., 2015). Most of the liver diseases that have been successfully modeled originate from patients with Mendelian inherited mutations that show robust phenotypes. Examples include familial hypercholesterolemia and α-1-antitrypsin deficiency, which are caused by mutations in the *LDLR* (*Low-Density Lipoprotein Receptor*) and *SERPINA1* (*Serpin Peptidase Inhibitor, Clade A [Alpha-1 Antiproteinase, Antitrypsin], Member 1*) genes, respectively (Rashid et al., 2010; Cayo et al., 2012; Tafaleng et al., 2015; Choi et al., 2013). While such successes are encouraging, it has become clear that complex traits and subtle phenotypes are more challenging to reproduce, primarily due to variations in the efficiency of differentiation among cells. Most, if not all, differentiation protocols generate iPSC-derived hepatocyte-like cells with heterogeneous expression of mature hepatic markers.

The generation of antibodies that facilitate purification of homogeneous subpopulations of cells has significantly advanced the study of lymphoid lineages and hematopoietic stem cells (Lanier et al., 1983; Spangrude et al., 1988). More recently, similar approaches have been used to identify and characterize subpopulations of liver cells in mice with progenitor cell characteristics (Dorrell et al., 2008, 2011). Antibodies that recognize cell-surface antigens have historically been generated by immunization of recipient animals with whole cells or membrane fractions. Antibodies that specifically recognize a given cell type are then identified by secondary screens. This procedure has recently been used successfully to identify monoclonal antibodies that can detect iPSC-derived endoderm cells that have an enhanced propensity to adopt a hepatic fate (Holtzinger et al., 2015). Although such an approach has been invaluable, the nature of the antigen can be complex and identification of epitopes challenging.

As an alternative to antibody-based methods, we used the chemoproteomic cell surface capture technology (CSC-Technology; Wollscheid et al., 2009; Gundry et al., 2012), which incorporates selective labeling, enrichment,

This is an open access article under the CC BY license (http://creativecommons.org/licenses/by/4.0/).

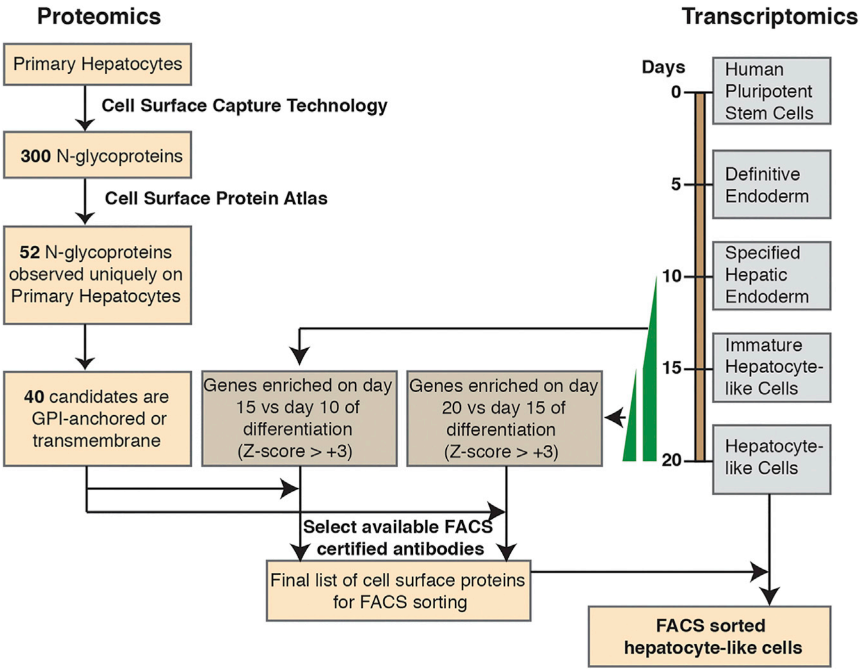

Figure 1. Identification of Cell-Surface Proteins for Purifying a Subpopulation of iPSC-Derived Hepatocyte-like Cells
A schematic overview of the approach used to identify cell-surface N-glycoproteins and candidate markers for hepatocyte sorting. Proteomic and oligonucleotide array analyses were combined to identify cell-surface N-glycoproteins that are enriched in hepatocyte-like cells that are derived from iPSCs.

and mass-spectrometry-based identification of extracellular domains of cell-surface N-glycoproteins to discover accessible surface markers on human hepatocytes. By establishing a “surfaceome” of human hepatocytes, this resource reveals targets for fluorescence-activated cell sorting (FACS)-based isolation, contributes to our knowledge of N-glycoproteins that define the hepatocyte phenotype, and is a first step toward revealing N-glycoproteins with potentially important roles in hepatocyte function and disease. Using a combination of proteomic and transcriptional profiling along with antibody-based sorting, we revealed that SLC10A1, CLRN3, and AADAC are cell-surface N-glycoproteins that are present on a common population of iPSC-derived hepatocyte-like cells that express elevated levels of several hepatic markers. Transcriptional profiling of purified SLC10A1-positive cells revealed enrichment of hepatic character, improved homogeneity, and depletion of cells expressing pluripotent markers.

RESULTS

Cell-Surface N-Glycoproteins on Primary Human Hepatocytes

To identify cell-surface N-glycoproteins that could potentially be used to purify homogeneous populations of iPSC-derived hepatocytes, we incorporated proteomic and transcriptomic analyses of primary hepatocytes and iPSC-derived cells (Figure 1). We first used the CSC-Technology to identify extracellular domains of cell-surface N-glycoproteins on primary human hepatocytes. This approach (Figure 2A) exploits biotinylation of the oligosaccharide structure attached to the extracellular domain of cell-surface glycoproteins on live cells. Following enzymatic digestion, biotinylated glycopeptides are enriched by affinity chromatography using immobilized streptavidin. Finally, the enzyme N-glycosidase F (PNGaseF), which cleaves between the innermost GlcNAc and the asparagine residue, selectively releases the formerly N-glycosylated peptides for subsequent analysis by high mass accuracy mass spectrometry analysis. Overall, this strategy provides for highly specific identification of cell-surface N-glycoproteins, including transmembrane, glycophosphatidylinositol (GPI)-anchored, and extracellular matrix, with minimal contamination from intracellular membrane proteins (Wollscheid et al., 2009; Bausch-Fluck et al., 2015; Boheler et al., 2014).

Proteins identified in this workflow are confidently assigned as originating from the cell surface if they are identified by at least one peptide containing a deamidated asparagine (resulting from PNGaseF cleavage) within the conserved sequence motif for N-glycosylation (NxS/T/C), although the majority of N-glycoproteins identified here are represented by multiple peptides (Table S1). An annotated tandem mass spectrometry (MS/MS) spectrum for a peptide derived from SLC10A1, illustrating the deamidation within the sequence motif is shown in Figure 2B. Using the above criteria, 300 cell-surface N-glycoproteins, including 66 cluster of differentiation (CD) molecules were identified on primary human hepatocytes

(Figure 2C). A complete list of all N-glycoproteins that were identified is provided in Table S1. Functional annotation using DAVID reveals that of the 300 N-glycoproteins identified, 292 are classified as glycoproteins and 228 as transmembrane proteins (Figure S1A). Gene ontology analyses revealed, as expected, that functions typically associated with transmembrane proteins, including receptors, signal transduction, and transporter activity, were highly represented (Figure S1B).

Identification of N-Glycoproteins That Are Enriched in Primary Hepatocytes

As discussed above, the ability to generate hepatocyte-like cells from iPSCs has enabled the modeling of inborn errors of hepatic metabolism in culture (Cayo et al., 2012; Rashid et al., 2010). However, heterogeneity in the differentiated cell population can complicate the interpretation of results. We reasoned that relatively homogeneous populations of cells could be generated by FACS using antibodies that recognize cell-surface N-glycoproteins that are enriched in hepatocytes. To date, Asialoglycoprotein Receptor 1 (ASGR1) has been used for hepatocyte enrichment (Basma et al., 2009; Peters et al., 2016). Although ASGR1 is highly expressed in the liver, it is also abundant in the gall bladder (Wilhelm et al., 2014). Moreover, studies of hematopoietic cells have revealed that increasing the repertoire of cell-surface proteins available for sorting promotes specificity (Lanier et al., 1983; Spangrude et al., 1988). We, therefore, sought to identify additional proteins whose presence on the hepatocyte cell surface may facilitate the production of homogeneous populations of hepatocyte-like cells from differentiating iPSCs.

We reasoned that N-glycoproteins whose expression was robust and restricted to hepatocytes would be the most suitable for purifying hepatocytes from iPSCs. An extensive searchable database, the Cell Surface Protein Atlas (Atlas), which presents the surfaceome of a broad variety of 47 human cell types, has recently been made available (Bausch-Fluck et al., 2015). We exploited this database along with surfaceome of human embryonic stem cells (ESCs) and iPSCs to obtain the frequency of detection of each protein identified in primary hepatocytes (Figure 2C) (Boheler et al., 2014). Protein distribution frequency ranged from proteins detected only in primary hepatocytes to those whose presence was ubiquitous. As with any mass spectrometry, failure to identify a particular protein does not irrefutably confirm the absence of that protein from the surface of that cell type. Rather, this resource is most valuable as a first step to eliminate proteins that are observed across many cell types and to narrow the focus to proteins that may be informative of a cell type of interest. Of the 300 N-glycoproteins identified, 52 were detected only in primary hepatocytes and 40 of those were annotated as transmembrane or GPI-anchored proteins (Figure 2C; Table S1). As our goal was to identify cell-surface N-glycoproteins that could be used for live cell sorting, we focused our subsequent analyses on these 40 hepatocyte-enriched transmembrane or GPI-anchored proteins (Table 1).

A Subset of Hepatocyte-Enriched N-Glycoproteins Are Induced during the Differentiation of Human Pluripotent Stem Cells into Hepatocyte-like Cells

To further narrow the 40 candidates down to those with the most potential for sorting iPSC-derived hepatocyte-like cells, we focused on those N-glycoproteins for which mRNA levels are most robustly expressed in iPSC-derived hepatocyte-like cells and induced during the final stages of differentiation when the hepatocytes reach relative maturity (Figure 1). We have previously described a protocol for the efficient and reproducible differentiation of human ESCs and iPSCs into hepatocyte-like cells using defined culture conditions (Si-Tayeb et al., 2010a; Mallanna and Duncan, 2013) The procedure recapitulates the stages of hepatocyte differentiation that occur during embryogenesis. Definitive endoderm is produced by day 5, endoderm is converted to a hepatic fate by day 10, fetal hepatocyte-like cells are formed by day 15, and cells with more mature hepatocyte characteristics are generated by day 20 (Mallanna and Duncan, 2013; Si-Tayeb et al., 2010a; Yu et al., 2012). We recently established mRNA profiles from each stage of the differentiation process using oligonucleotide arrays (Si-Tayeb et al., 2010a; Delaforest et al., 2011). Using this approach, we examined the temporal expression pattern of mRNAs encoding the 40 hepatocyte-enriched cell-surface N-glycoproteins during differentiation. The mRNA levels encoding six of the proteins (CLRN3, KLB, SLC22A7, AADAC, SLC10A1, and UGT2B4) were found to be enriched $\geq$8-fold either by day 15 or day 20, coincident with when the cells establish hepatocyte function (Figure 3A). We finally used a genetic approach to examine whether the increases in these mRNA levels was due to expression in hepatocytes (Figure 3B). The transcription factor HNF4A is essential for hepatocyte differentiation and when HNF4A is depleted, PSCs are incapable of adopting a hepatic fate (Delaforest et al., 2011; Li et al., 2000; Parviz et al., 2003). We predicted that if the mRNAs encoding the candidate N-glycoproteins were selectively expressed in ESC-derived hepatocytes, the relative abundance of these mRNAs should be depleted in the absence of HNF4A. As shown in Figure 3B, in contrast to day 20 hepatocytes derived from control ESCs in which mRNAs encoding the six N-glycoproteins were readily detected by oligonucleotide array analyses, these same mRNAs were undetectable in hepatocytes derived from HNF4A-depleted ESCs.

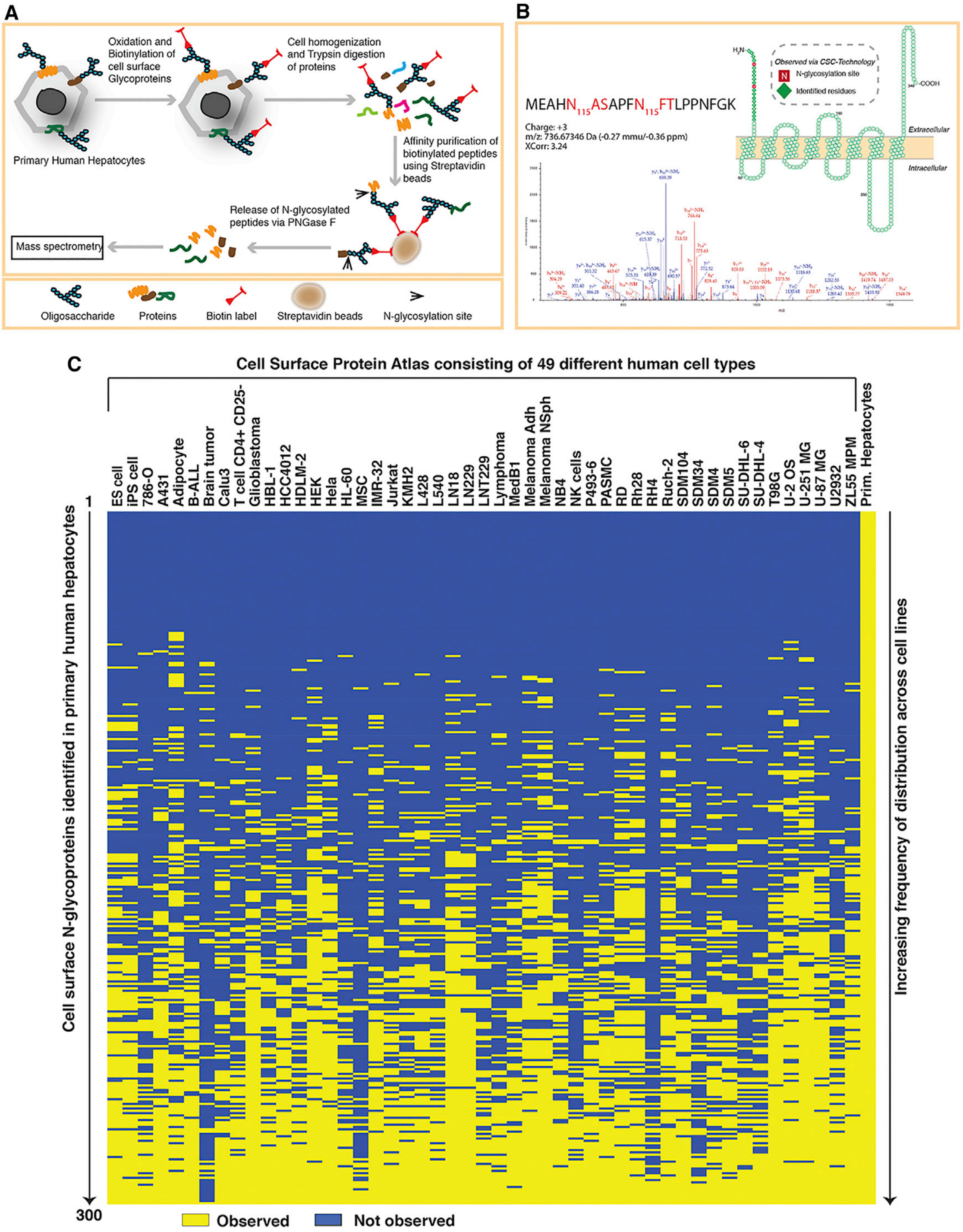

Figure 2. Identification of N-Linked Glycoproteins in Primary Hepatocytes by Cell Surface Capture Technology

(A) Illustration outlining the CSC-Technology.

(B) Left: Annotated MS/MS spectrum of a representative peptide from SLC10A1 that was identified in primary hepatocytes by CSC-Technology. Two identified sites of N-glycosylation (N_{115}) are indicated. Right: An illustration of the transmembrane topology of SLC10A1 highlighting the extracellular peptide identified by CSC-Technology, including sites of N-glycosylation (also see Figure S2). Image generated using Protter (Omasits et al., 2014).

(legend continued on next page)

SLC10A1, CLRN3, and AADAC Are Co-expressed in a Subset of iPSC-Derived Hepatocytes

Of the six N-glycoproteins that were selected as hepatocyte markers, commercially available antibodies that were compatible with FACS were available for SLC10A1, CLRN3, and AADAC. Representative MS/MS spectra and peptide sequences from the CSC-Technology analyses are provided for each of these proteins (Figure S2) and all corresponding peptide details are in Table S1. Oligonucleotide array analyses performed on differentiating ESCs indicated that the mRNAs encoding these proteins were induced as the cells approached maturity (day 20; Figure 3A). We therefore attempted to confirm these expression profiles on independent differentiations of iPSCs by qRT-PCR. As expected, like *Albumin* and *ASGR1*, which were used as markers of differentiation, *SLC10A1, CLRN3*, and *AADAC* mRNAs were close to undetectable in PSCs (day 0), definitive endoderm cells (day 5), and hepatic progenitor cells (day 10) (Figure 3C). Consistent with the oligonucleotide array data, we observed a large induction of *CLRN3* mRNA at day 15, which continued through day 20. *SLC10A1* and *AADAC* transcript levels remained low at day 15 then increased substantially by day 20 of differentiation (Figure 3C). Although *SLC10A1, CLRN3*, and *AADAC* mRNAs were reproducibly induced as the iPSC-derived hepatocytes entered a maturation phase, it is important to note that a comparison of the mRNA levels found in iPSC-derived hepatocytes with those found in primary hepatocytes revealed them to be significantly lower in the iPSC- and ESC-derived cells (Figure 3D). Similar results were obtained when qRT-PCR was performed on hepatocyte-like cells derived from either H1 (WA01) or H9 (WA09) human ESCs (Figure S3A).

We reasoned that the relatively low levels of mRNAs encoding SLC10A1, CLRN3, and AADAC observed in the iPSC-derived hepatocytes could be due to low expression throughout the entire population of cells or alternatively that expression is restricted to a subpopulation. To distinguish between these possibilities, we examined the cellular distribution of SLC10A1, CLRN3, and AADAC proteins in iPSC-derived hepatocytes by immunocytochemistry and live cell flow cytometry (Figure 4). Confocal imaging of iPSC-derived hepatocytes revealed that the target proteins were uniformly detected throughout the cell membranes but were present on a subpopulation of differentiated cells (Figure 4A). Next, flow cytometry was used to quantify the percent positive population. These analyses revealed that 20%–25% of the total population was positive for each of these cell-surface N-glycoproteins (Figure 4B). To confirm the identity of the SLC10A1-, CLRN3-, and AADAC-positive cells, co-staining experiments using an antibody that recognizes the hepatocyte transcription factor HNF4A were performed. By day 20 of differentiation, >90% of cells expressed HNF4A (Figure 4C). However, while nearly all of the SLC10A1-, CLRN3-, or AADAC-positive cells were also positive for HNF4A, only a subpopulation of HNF4A-positive cells were positive for SLC10A1, CLRN3, or AADAC (Figure 4C; note that fixation conditions required to detect HNF4A resulted in non-specific binding of the anti-AADAC antibody). Pairwise co-staining revealed that SLC10A1, CLRN3, and AADAC are expressed on the same subpopulation of iPSC-derived hepatocytes (Figure S3B).

All these experiments were performed using a single iPSC line (iPSC-K3) that was derived from foreskin fibroblasts as we have described previously (Si-Tayeb et al., 2010b). To exclude the possibility that the heterogeneous expression of SLC10A1, CLRN3, and AADAC reflected any peculiarity of K3 cells, we repeated our analyses on hepatocytes derived from an independent iPSC line (SV20) that was generated from peripheral blood mononuclear cells from an independent donor (Yang et al., 2015). Similar to using K3 iPSCs, SLC10A1, CLRN3, and AADAC were co-expressed in ~25% of SV20 iPSC-derived hepatocytes (Figure S3C). Based on these results, we conclude that SLC10A1, CLRN3, and AADAC are expressed on a common subpopulation of iPSC-derived hepatocytes. Since ASGR1 has been used by others to purify iPSC-derived hepatocytes by FACS, we compared the distribution of ASGR1 protein with SLC10A1, CLRN3, and AADAC by immunostaining. Although ASGR1 was more broadly expressed, all SLC10A1, CLRN3, and AADAC positive cells also expressed ASGR1 (Figure S4). These results confirm that although the vast majority of cells derived from the iPSCs have adopted a hepatic fate and are HNF4A positive, expression of mature hepatic markers is heterogeneous.

SLC10A1 Purified iPSC-Derived Hepatocytes Demonstrate Improved Hepatic Phenotype, Depletion of Pluripotent Cell Markers, and Increased Homogeneity

Immunohistochemistry performed on human liver sections has revealed that SLC10A1, CLRN3, and AADAC are expressed on >75% of the liver's hepatocytes (data provided by the Human Protein Atlas; Uhlen et al., 2010;

(C) Graphical representation of the distribution of proteins identified in primary hepatocytes. Each column represents an individual cell type documented in the Cell Surface Protein Atlas, which consists of 49 different cell types. The last column shows proteins identified on the surface of primary hepatocytes described in the current study. Each row represents a single protein. A yellow bar indicates the protein was reported as present in the cell line, whereas blue indicates that it was not observed. The frequency of distribution of each protein is lowest at the top and highest at the bottom. The identity of each protein in the order presented is provided in Table S1.

Table 1. 40 Cell Surface N-Glycoproteins Identified as Hepatocyte-Restricted Compared with 49 Other Human Cell Types

Gene Symbol	Protein Name	Type
AADAC	arylacetamide deacetylase	TM
ABCA6	ATP-binding cassette sub-family A member 6	TM
ABCB11	bile salt export pump	TM
ABCC6	multidrug resistance-associated protein 6	TM
ABCG8	ATP-binding cassette sub-family G member 8	TM
ANO1	anoctamin-1	TM
AQP9	aquaporin-9	TM
ART4	ecto-ADP-ribosyltransferase 4	GPI
ASGR1	asialoglycoprotein receptor 1	TM
ASGR2	asialoglycoprotein receptor 2	TM
C8A	complement component C8 alpha chain	TM
CDHR5	cadherin-related family member 5	TM
CLRN3	Clarin-3	TM
FAM151A	protein FAM151A	TM
FOLH1	glutamate carboxypeptidase 2	TM
HFE2	hemojuvelin	GPI
KLB	β-klotho	TM
NPC1L1	Niemann-Pick C1-like protein 1	TM
SLC10A1	sodium/bile acid cotransporter	TM
SLC1A2	excitatory amino acid transporter 2	TM
SLC22A1	solute carrier family 22 member 1	TM
SLC22A3	solute carrier family 22 member 3	TM
SLC22A7	solute carrier family 22 member 7	TM
SLC22A9	solute carrier family 22 member 9	TM
SLC2A8	solute carrier family 2, facilitated glucose transporter member 8	TM
SLC39A5	zinc transporter ZIP5	TM
SLC46A3	solute carrier family 46 member 3	TM
SLC6A12	sodium- and chloride-dependent betaine transporter	TM
SLCO1B1	solute carrier organic anion transporter family member 1B1	TM
SLCO1B3	solute carrier organic anion transporter family member 1B3	TM
TFR2	transferrin receptor protein 2	TM
TM4SF4	transmembrane 4 L6 family member 4	TM
TM4SF5	transmembrane 4 L6 family member 5	TM
TMEM110	store-operated calcium entry regulator STIMATE	TM
TMPRSS6	transmembrane protease serine 6	TM
TMUB1	transmembrane and ubiquitin-like domain-containing protein 1	TM
UGT1A4	UDP-glucuronosyltransferase 1-4	TM
UGT2B15	UDP-glucuronosyltransferase 2B15	TM
UGT2B4	UDP-glucuronosyltransferase 2B4	TM
UGT2B7	UDP-glucuronosyltransferase 2B7	TM

TM, transmembrane; GPI, glycophosphatidylinositol.

Uhlén et al., 2015). The broad distribution of these cell-surface proteins throughout the hepatic parenchyma implies that they could facilitate FACS-based purification of a homogeneous population of iPSC-derived cells with hepatocyte characteristics. To test this hypothesis, we used antibodies to SLC10A1, CLRN3, or AADAC and FACS to collect populations of iPSC-derived hepatocytes at day 20 of differentiation and subsequently measured abundance of characteristic hepatic mRNAs in the sorted cells by qRT-PCR. Gating parameters for FACS for SLC10A1-positive iPSC-derived hepatocytes is shown in Figure S5A. As shown in Figure 5, *SLC10A1* mRNA was enriched in SLC10A1-positive cells by ~3-fold compared with the levels found in pre-sorted cells and was close to undetectable in the SLC10A1-negative population. Analyses of mRNAs encoding CLRN3, AADAC, HNF4A, albumin, ASGR1, APOA1, and AFP by qRT-PCR revealed that each of these hepatocyte markers was significantly enriched in the SLC10A1-positive population compared with both the SLC10A1-negative cells and the pre-sorted cells (Figure 5; Figure S5B). Similar results were obtained when FACS was performed to collect CLRN3- and AADAC-positive cells (Figures S5C and S5D).

Finally, we examined whether FACS selection of SLC10A1-positive iPSC-derived hepatocytes improved overall hepatic characteristics and homogeneity. Four independent iPSC differentiations were performed until day 20, and the cells were subjected to FACS using an anti-SLC10A1 antibody. Pre-sort, SLC10A1-positive, and SLC10A1-negative populations were then subjected to transcriptome analyses using oligonucleotide arrays. Cell profiles were compared using CellNet (Cahan et al., 2014;

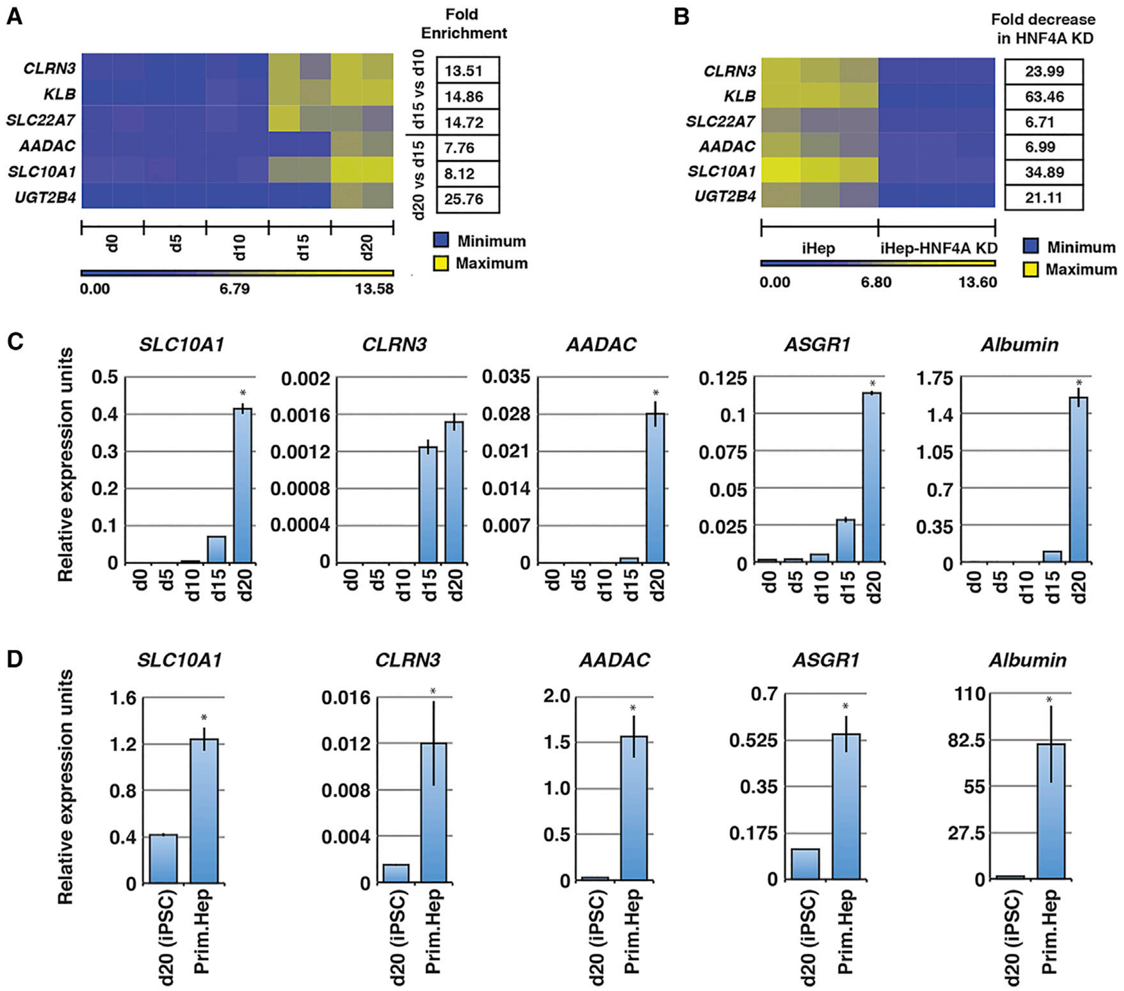

Figure 3. Selection of Cell Surface Proteins for Purifying iPSC-Derived Hepatocyte-like Cells

(A) Heatmap showing enrichment of six transcripts encoding hepatocyte-restricted cell surface glycoproteins (CLRN3, KLB, SLC22A7, AADAC, SLC10A1, and UGT2B4) during the differentiation of human ESCs to hepatocyte-like cells. Published oligonucleotide array data from biological replicates at days 0, 5, 10, 15, and 20 of differentiation were analyzed (Si-Tayeb et al., 2010a; Delaforest et al., 2011). Fold enrichment of the six transcripts between days 10 and 15 or days 15 and 20 is given next to the heatmap.

(B) Published oligonucleotide expression data were used to examine transcript levels encoding CLRN3, KLB, SLC22A7, AADAC, SLC10A1, and UGT2B4 in hepatocyte-like cells generated from wild-type ESCs and ESCs in which HNF4A is depleted (Delaforest et al., 2011). Fold decrease in the level of transcripts following HNF4A depletion is given next to the heatmap.

(C) Bar graphs of qRT-PCR results showing levels of mRNAs encoding cell-surface proteins SLC10A1, CLRN3, AADAC, ASGR1, and albumin on days 0, 5, 10, 15, and 20 of differentiation of iPSCs into hepatocyte-like cells. Data are represented as means ± SEM of biological replicates ($n = 3$). Significance of enrichment in day 20 cells compared with day 15 cells was determined by Student's t test, $*p < 0.05$.

(D) Bar graphs of qRT-PCR results showing relative expression levels of mRNAs encoding SLC10A1, CLRN3, AADAC, ASGR1, and albumin in iPSC-derived hepatocyte-like cells in comparison with primary hepatocytes (see also Figure S3A). Data are represented as means ± SEM of biological replicates ($n = 3$). Significance was determined by Student's t test, $*p < 0.05$.

Morris et al., 2014), which is a computational platform designed to define the similarity between stem-cell-derived cell types and their endogenous counterparts by comparing the expression levels of gene regulatory networks (Figure 6A; Table S2). A higher classification score indicates an increase in the probability that expression of a gene regulatory network resembles that of a target tissue (Cahan et al., 2014; Morris et al., 2014). As expected, in the pre-sorted population, the differentiated cells most closely resembled liver compared with 14 other tissue types. Surprisingly, however, pluripotent cell characteristics were also predominant in the pre-sorted population, suggesting that the cultures contained cells that had failed to differentiate or had retained expression of PSC markers (Figure 6A).

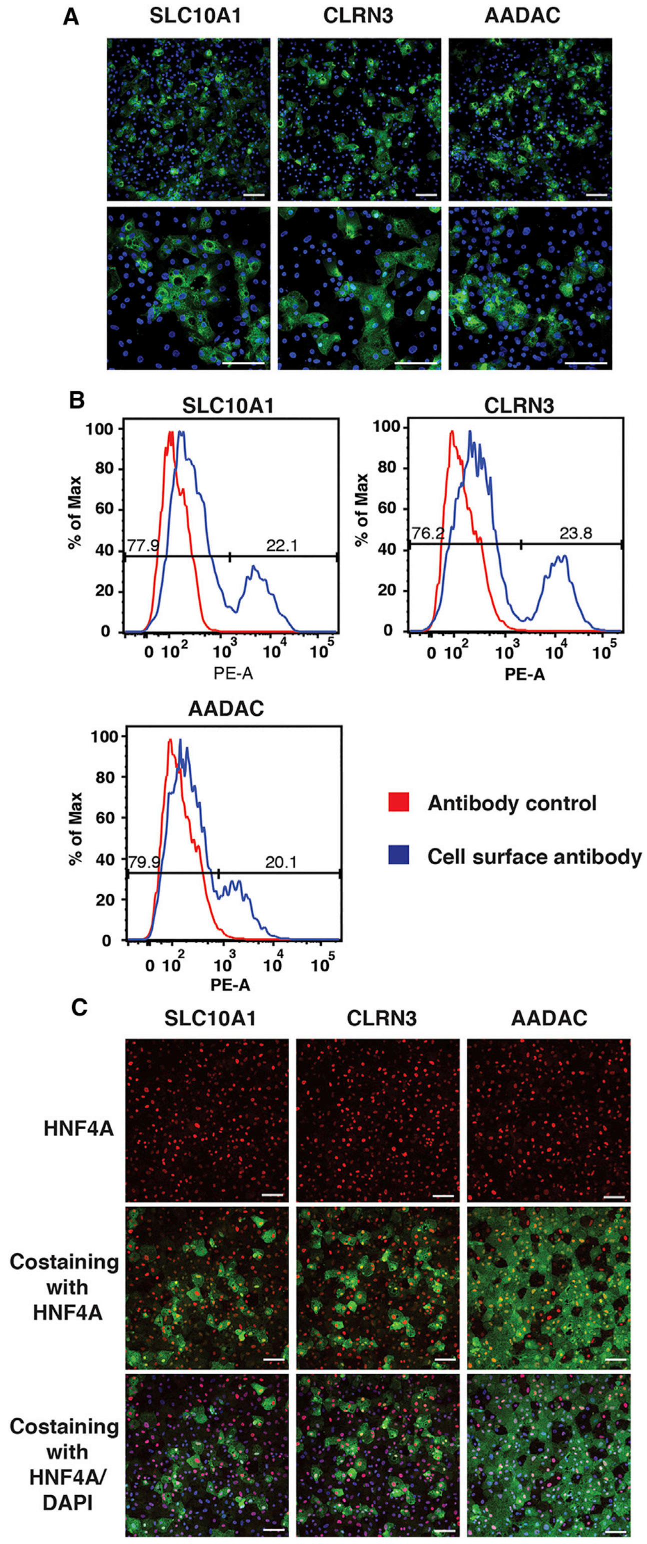

Figure 4. A Subpopulation of iPSC-Derived Hepatocyte-like Cells Express SLC10A1, CLRN3, and AADAC

(A) Confocal micrographs showing the results of immunocytochemistry to detect expression of cell-surface proteins SLC10A1, CLRN3, and AADAC in iPSC-derived hepatocyte-like cells (green). Nuclei are identified by DAPI staining (blue) (see also Figures S3B and S3C). Scale bars, 100 μm.

(B) FACS histogram plots of iPSC-derived hepatocyte-like cells stained with primary antibodies against proteins SLC10A1, CLRN3, and AADAC and corresponding phycoerythrin (PE) conjugated secondary antibody are shown. Cells stained with PE conjugated secondary antibody only are used as control.

(C) Confocal micrographs showing the results of immunocytochemistry to detect expression of HNF4A (red) and cell-surface proteins SLC10A1, CLRN3, and AADAC (green) in iPSC-derived hepatocyte-like cells (see also Figure S4). Nuclei are identified by DAPI staining (blue). Scale bars, 100 μm.

When the character of the SLC10A1-expressing cell population was examined, it revealed that the cells had substantially increased hepatic characteristics and reduced pluripotent cell characteristics. In contrast to the SLC10A-positive population, the SLC10A1-negative population lost hepatic characteristics and retained PSC characteristics. We confirmed that the SLC10A1-positive cells more closely resembled hepatocytes compared with the unsorted and SLC10A1-negative cells by examining the levels of mRNAs encoding 39 proteins that are highly enriched in liver tissue (Ge et al., 2005; Si-Tayeb et al., 2010a). These hepatic mRNAs were consistently enriched in the SLC10A1-positive cell population compared with unsorted and negative cell populations (Figure 6B; Table S3).

Although hepatocyte-like cells generated from independent differentiations looked morphologically indistinguishable (Figure S6A), we observed substantial variation in expression of hepatic markers among samples, confirming heterogeneity in the extent of maturation (Figures 6B and 6C; Table S3). However, in contrast to unsorted cells, the SLC10A1-positive population shows uniform levels of hepatic mRNAs among independent differentiations (Figures 6B and 6C; Table S3). These data demonstrate that sorting hepatocyte-like cells using an anti-SLC10A1 antibody facilitates the efficient purification of a homogeneous population of cells with hepatocyte characteristics.

DISCUSSION

Hepatocyte-like cells generated from iPSCs can be used to model liver development and disease, for toxicity studies, and potentially could offer a source of hepatocytes for cell therapy (Rashid et al., 2010; Delaforest et al., 2011; Cayo et al., 2012; Fox et al., 2014). While the promise of such cells is exciting, improvements to the existing differentiation protocols are necessary to obtain a homogeneous and mature population of hepatocytes whose function recapitulates the native parenchymal cells of the liver. In the current study, we addressed the issue of heterogeneity

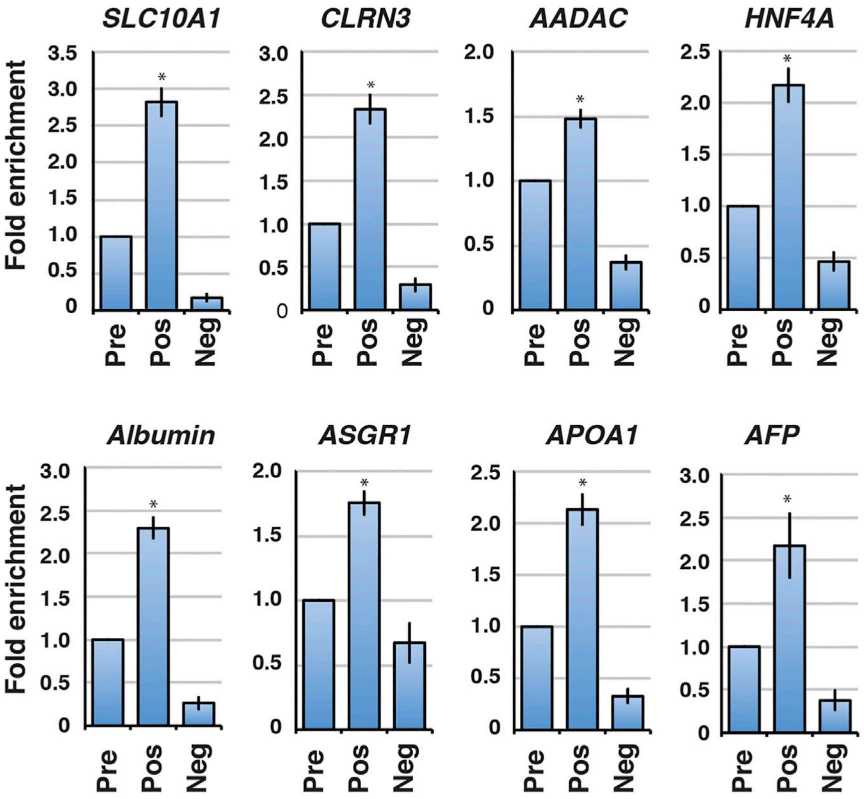

Figure 5. A Subpopulation of iPSC-Derived Hepatocyte-like Cells that Express SLC10A1 Is Enriched for Several Liver-Specific Markers

iPSC-derived hepatocyte-like cells were sorted by FACS using an antibody against SLC10A1 (see also Figure S5A). Bar graphs show qRT-PCR analysis to determine the levels of mRNAs encoding the liver-enriched proteins (HNF4A, albumin, ASGR1, APOA1, AFP) and cell-surface proteins (SLC10A1, CLRN3, AADAC) in the SLC10A1-positive and -negative cell fractions. Levels of mRNAs were compared with the pre-sorted cell population that is set to 1.0 (see also Figures S5B–S5D). To avoid any bias introduced by the sorting process, the pre-sort population was collected as the gated viable cell fraction after passing through the FACS. Data are represented as means ± SEM from sorting performed on three independent differentiations (n = 3). Significance of enrichment in the SLC10A1-positive population compared with the pre-sorted population was determined by Student's t test, $^*p < 0.05$.

associated with the differentiation procedure by identifying cell-surface markers that facilitate the purification of homogeneous cell populations.

Traditionally, identification and characterization of cell-surface proteins heavily relied on the availability of antibodies. In such cases, the need for well-characterized antibodies invariably biases study design to focus on CD antigens that are defined by their interaction with monoclonal antibodies (Lanier et al., 1983; Spangrude et al., 1988). Such focus, while not without value, limits the potential repertoire of target proteins that could be used for cell sorting. In contrast, mass spectrometry offers the advantage of being able to identify, characterize, and quantify cell-surface proteins independently of the availability of suitable antibodies. Specifically in the case of the CSC-Technology, the approach exploits the fact that the majority of cell-surface proteins are glycosylated to enable specific detection of transmembrane, GPI-anchored, and extracellular matrix proteins. Moreover, as secreted proteins are also commonly modified by N-linked glycosylation, the CSC-Technology can also detect proteins that are transiting through the secretory pathway, especially if they are abundant. This explains why 71 of 300 proteins identified in our study are annotated as secreted by DAVID. Examples include Apolipoprotein B-100, α-1-antitrypsin, and Coagulation factor V, all of which are abundant serum factors that are known to be secreted by hepatocytes. Although the focus of the current study was to identify proteins that can be used to sort hepatocytes that are derived from iPSCs, the availability of an extensive catalog of proteins present on the hepatocyte cell surface should also be valuable for the study of hepatocyte biology and liver disease. The biological roles of many of the identified proteins within the hepatocyte are poorly understood. This includes one of our target proteins Clarin 3, which is a multi-pass transmembrane protein that has restricted cell distribution among the cell types within the Atlas. While the function of this protein is poorly understood, mutations in a paralog, Clarin 1, have been associated with Usher syndrome, which causes loss of vision and hearing (Joensuu et al., 2001). In addition to providing tools to advance our understanding of liver cell biology, the expanding database of hepatocyte cell-surface proteins should facilitate the identification of receptors involved in infectious disease. In this regard, SLC10A1 (also known as NTCP) has recently been described as a receptor for the hepatitis B and D viruses (Yan et al., 2012). It is, therefore, likely that receptors for known and emerging hepatotropic infectious agents will be present within the catalog of hepatocyte cell-surface proteins we have described.

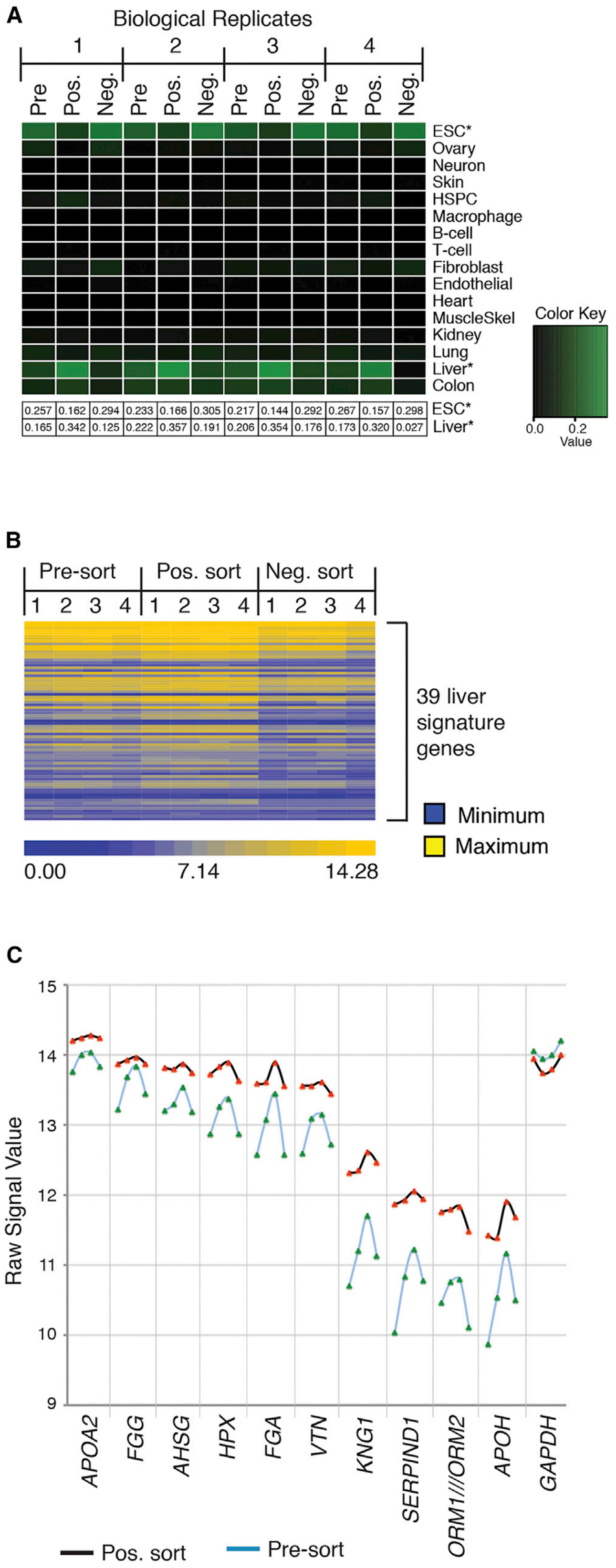

Figure 6. SLC10A1-Positive iPSC-Derived Hepatocyte-like Cells Are Enriched for Hepatic Gene Regulatory Genes and Sorting Them Minimizes Variability Associated with Differentiation

(A) Microarray analysis was performed on different cell fractions of iPSC-derived hepatocyte-like cells sorted for expression of SLC10A1. CellNet classification heatmap showing enrichment of liver-specific GRN genes and depletion of ESC-specific GRN genes in the positive sort cell fraction compared with the pre-sort and negative sort cell fractions. Classification scores corresponding to the ESC- and liver-specific GRN genes from the heatmap are presented below the heatmap. Classification scores for different target tissue GRNs represented in the heatmap are provided in Table S2. Microarray data on the pre-sort, positive sort, and negative sort cell fractions from four independent differentiations were used for CellNet analysis (n = 4) (see also Figure S6).

(B) Heatmap showing expression levels of 39 liver signature genes in pre-sort, positive sort, and negative sort cell fractions. Probe sets of 39 liver signature genes and the corresponding raw signal values in different cell fractions are provided in Table S3 in the same order as in the heatmap. Microarray data on the pre-sort, positive sort, and negative sort cell fractions from four independent differentiations were used to generate the heatmap (n = 4).

(C) Line diagram showing raw signal values of the top ten abundant transcripts selected from 39 liver signature genes in pre-sort and SLC10A1-positive sort cell fractions of hepatocyte-like cells. Signal values of GAPDH transcript served as internal control. Microarray data from four independent differentiations were used to generate the line diagram (n = 4) (see also Figure S6).

Previous studies investigating the N-glycoproteome in human liver/hepatocytes are limited. Several proteomic studies have focused on the identification of N-glycoproteins in liver tissue extracts (Sun et al., 2014; Chen et al., 2009; Zhu et al., 2014). Although these approaches provide important information regarding the repertoire of proteins present in the liver, they were not designed to specifically identify cell-surface proteins. Importantly, using whole-liver tissue extract limits the specificity of markers due to the presence of a mixed cell population in the sample. In contrast, Ducret et al. (2015) used a cell-surface capture approach to identify 147 cell-surface N-glycoproteins in primary human hepatocytes. Of the 300 proteins identified in our analyses, Ducret et al. also identified 114 and 186 are unique to our study. Differences in technical approach, including the number of hepatocytes analyzed, likely explain the differences between these two studies. Of note, CLRN3, KLB, SLC22A7, AADAC, SLC10A1, and UGT2B4 were not described as liver-enriched proteins by Ducret et al., which would imply that this screen was not saturated.

The ability to use cell-surface proteins to specifically sort iPSC-derived hepatocyte-like cells is currently limited. The liver-specific cell-surface protein ASGR1 has been used to sort human ESC- and iPSC-derived hepatocyte-like cells (Basma et al., 2009; Peters et al., 2016). These studies

demonstrated enrichment of hepatic phenotype in ASGR1-positive cells by performing transcriptional analysis and functional assays. Although we identified ASGR1 on the cell surface of primary hepatocytes, its transcript expression pattern during hPSC differentiation into hepatocyte-like cells did not meet the stringent enrichment criteria we required to select the final candidate proteins for our studies. More recently, microRNA switches have been used to enrich for a desired population of hepatocyte-like cells generated by directed differentiation (Miki et al., 2015). In this approach, reporter mRNAs containing target sequences for miRNAs expressed in the desired cell type are transfected, and diminished reporter expression enables sorting of the target cell population. Although useful, transfection adds technical variability, and differences in transfection efficiency of reporter mRNAs could contribute to differences in the cell types sorted from distinct biological samples. With this in mind, we believe the use of endogenous proteins that are accessible to affinity reagents for live cell sorting will prove most apt for the selection of homogeneous cell populations suitable for research and clinical applications.

Finally, we provide evidence that cell sorting using antibodies to either SLC10A1, CLRN3, or AADAC can improve the quality of hepatocyte-like cells generated from iPSCs. Cell populations resulting from positive selection based on these markers displayed higher levels of characteristic hepatic mRNAs. Importantly, a comparison of marker expression among four independent differentiations revealed that the cells sorted based on the presence of SLC10A1 had substantially lower variation in expression of characteristic mRNAs than the unsorted population. This implies that sorting cells using SLC10A1, CLRN3, or AADAC will improve comparisons among control and patient iPSC-derived hepatocytes. Minimizing heterogeneity within cultures of iPSC-derived cells should facilitate our ability to compare and interpret data resulting from hepatocytes generated from iPSCs that model metabolic liver disease. Until now, because of variations with differentiation, analyses of disease phenotypes have been limited to patients with Mendelian disorders. However, the ability to generate homogeneous populations of iPSC-derived hepatocytes should facilitate analyses of complex phenotypes. For example, several common allelic variations that associate with lipid traits have been identified by genome-wide association studies (Teslovich et al., 2010). Producing homogeneous populations of iPSC-derived hepatocytes from either patient iPSCs or iPSCs modified using genome editing approaches could facilitate direct testing of a specific variant's contribution to levels of secreted lipid.

Use of recombinant laminins as substrates have recently been shown to improve cell organization, cell function, and phenotype of human ESC-derived hepatocyte-like cells (Cameron et al., 2015). Using data published by Cameron et al. (2015), we compared the liver-enriched gene regulatory network (GRN) of SLC10A1-positive cells with hepatocyte-like cells differentiated on recombinant laminin substrates. The GRN of SLC10A1-positive cells appeared to be comparable with hepatocyte-like cells differentiated on recombinant laminin substrates, although we acknowledge that differences in experimental protocol may introduce some error into this comparative analysis (Figure S6B). Importantly, however, SLC10A1-positive cells from independent differentiations exhibited less variation in the liver GRN compared with independent differentiations of hepatocyte-like cells on recombinant laminin substrates, which is consistent with the belief that sorting using such cell-surface markers can enhance the homogeneity of the hepatocyte-like cell population (Figure S6B). These analyses also suggest that by combining approaches, further advances in the quality and homogeneity of iPSC-derived hepatocytes when compared with fresh hepatocytes could be obtained (Figure S6C).

In conclusion, our study provides an expanded dataset of cell-surface N-glycoproteins on primary hepatocytes. We expect this resource, in conjunction with the bioinformatics workflow that includes the evolving Atlas as a platform to identify cell-type-restricted proteins, will be valuable for researchers investigating liver biology and pathophysiology. Importantly, we establish the functional utility of this resource and workflow by identifying three cell-surface N-glycoproteins capable of selecting a homogeneous subpopulation of iPSC-derived hepatocyte-like cells that display enhanced hepatic characteristics.

EXPERIMENTAL PROCEDURES

Cell Culture

Procedures for the culture of human iPSCs and their differentiation into hepatocyte-like cells have been described in detail previously (Mallanna and Duncan, 2013). The use of human cells was approved by the Medical College of Wisconsin Stem Cell Research Oversight and Institutional Safety Board committees.

CSC-Technology

Approximately 100 million primary human hepatocytes were subjected to CSC-Technology workflow as reported previously (Wollscheid et al., 2009; Gundry et al., 2012). Frozen primary human hepatocytes (Hu8176, Hu1443, Hu1460, and Hu1736) were obtained from Life Technologies/Thermo Fisher Scientific. Additional details are provided in Supplemental Experimental Procedures.

qRT-PCR Analysis

RNA was isolated from iPSC-derived hepatocyte-like cells and primary human hepatocytes using the RNeasy mini or micro kits (QIAGEN). Genomic DNA was removed using 1 μl of RNase-free

DNaseI per 5 μg of RNA. First-strand cDNA was synthesized using Moloney murine leukemia virus reverse transcriptase with dNTPs and random hexamer primers. qRT-PCR was performed using either StepOne Plus (Applied Biosystems/Thermo Fisher Scientific) or CFX-384 real-time PCR machine (Bio-Rad) with PrimeTime assays (Table S4) (Integrated DNA Technologies) following the manufacturer's protocols.

Immunostaining

For immunostaining cell-surface proteins (SLC10A1, CLRN3, AADAC, and ASGR1), fixed, non-permeabilized cells were incubated with antibodies against cell-surface proteins. For co-immunostaining for HNF4A, fixed non-permeabilized cells were incubated with antibodies against cell-surface proteins first, followed by fixing again, permeabilization, and incubation with HNF4A antibody. Finally, cells were incubated with appropriate secondary antibodies and nuclear stain DAPI to complete the staining. Additional details of the immunostaining protocol along with relevant antibody information are provided in the Supplemental Experimental Procedures.

Fluorescent-Activated Cell Sorting

A subpopulation of iPSC-derived hepatocyte-like cells expressing SLC10A1, CLRN3, and AADAC were sorted using the fluorescent-activated cell sorting (FACS) protocol described in detail in the Supplemental Experimental Procedures. Primary antibodies used were SLC10A1 (Aviva, ARP42097_P050), CLRN3 (Thermo Fisher Scientific, PA5-26137), and AADAC (LSBio, LS-C155827). Mouse monoclonal antibodies (B6E11 and C3B6) were generated commercially (Biomatik) to detect an external epitope on human SLC10A1 (YSRGIYDGDLKDKVPY). Hybridomas and the corresponding monoclonal antibodies have been made available to investigators through the Developmental Studies Hybridoma Bank created by the NICHD and maintained at the University of Iowa (http://dshb.biology.uiowa.edu/Welcome?search=slc10A1).

Oligonucleotide Array Analysis

Total RNA from different FACS-sorted fractions of iPSC-derived hepatocyte-like cells (pre-sort, SLC10A1 positive sort, SLC10A1 negative sort) from four independent differentiations was isolated using an RNeasy plus micro kit (QIAGEN). Biotinylated cRNA was generated from ~500 ng of total RNA using the 3′ IVT Plus kit and hybridized to GeneChip Human Genome U133 Plus 2.0 Arrays (Affymetrix). Images were acquired using a GeneChip Scanner 3000 (Affymetrix), and data analysis was performed using the Partek Genomic Suite Statistical Analysis Software (Partek).

ACCESSION NUMBERS

All original gene array CEL files are available from the Gene Expression Omnibus (GEO) accession number GEO: GSE80279.

SUPPLEMENTAL INFORMATION

Supplemental Information includes Supplemental Experimental Procedures, six figures, and four tables and can be found with this article online at http://dx.doi.org/10.1016/j.stemcr.2016.07.016.

AUTHOR CONTRIBUTIONS

S.K.M. and S.A.D. designed the study, analyzed the data, and co-wrote the manuscript; S.A.D. supervised the study; S.K.M. performed all experiments except when stated; M.A.C. performed immunostaining and flow cytometry using mouse monoclonal antibodies against SLC10A1; K.T. assisted with microarray experiments; R.L.G. supervised and assisted with CSC-Technology experiments and mass spectrometry data acquisition and analysis. All co-authors approved the final manuscript.

ACKNOWLEDGMENTS

The authors acknowledge Hope Campbell at Blood Center of Wisconsin for performing FACS sorting. This work was supported by gifts from the Marcus Family, the Phoebe R. and John D. Lewis Foundation, the Sophia Wolf Quadracci Memorial Fund, the Advancing a Healthier Wisconsin Fund, and by NIH grants DK102716, HG006398, and HD082570 to S.A.D. and NIH grant HL094708 to R.L.G. Confocal analyses were supported by COBRE P30 GM103342. We would like to dedicate this manuscript to the memory of John (Jack) Lewis (1927–2015).

Received: May 9, 2016
Revised: July 18, 2016
Accepted: July 20, 2016
Published: August 25, 2016

REFERENCES

Agarwal, S., Holton, K.L., and Lanza, R. (2008). Efficient differentiation of functional hepatocytes from human embryonic stem cells. Stem Cells *26*, 1117–1127.

Basma, H., Soto-Gutierrez, A., Yannam, G.R., Liu, L., Ito, R., Yamamoto, T., Ellis, E., Carson, S.D., Sato, S., Chen, Y., et al. (2009). Differentiation and transplantation of human embryonic stem cell-derived hepatocytes. Gastroenterology *136*, 990–999.

Bausch-Fluck, D., Hofmann, A., Bock, T., Frei, A.P., Cerciello, F., Jacobs, A., Moest, H., Omasits, U., Gundry, R.L., Yoon, C., et al. (2015). A mass spectrometric-derived cell surface protein atlas. PLoS One *10*, e0121314.

Boheler, K.R., Bhattacharya, S., Kropp, E.M., Chuppa, S., Riordon, D.R., Bausch-Fluck, D., Burridge, P.W., Wu, J.C., Wersto, R.P., Chan, G.C., et al. (2014). A human pluripotent stem cell surface N-glycoproteome resource reveals markers, extracellular epitopes, and drug targets. Stem Cell Rep. *3*, 185–203.

Cahan, P., Li, H., Morris, S.A., Lummertz da Rocha, E., Daley, G.Q., and Collins, J.J. (2014). CellNet: network biology applied to stem cell engineering. Cell *158*, 903–915.

Cai, J., Zhao, Y., Liu, Y., Ye, F., Song, Z., Qin, H., Meng, S., Chen, Y., Zhou, R., Song, X., et al. (2007). Directed differentiation of human embryonic stem cells into functional hepatic cells. Hepatology *45*, 1229–1239.

Cameron, K., Tan, R., Schmidt-Heck, W., Campos, G., Lyall, M.J., Wang, Y., Lucendo-Villarin, B., Szkolnicka, D., Bates, N., Kimber, S.J., et al. (2015). Recombinant laminins drive the differentiation

and self-organization of hESC-derived hepatocytes. Stem Cell Rep. *5*, 1250–1262.

Cayo, M.A., Cai, J., DeLaForest, A., Noto, F.K., Nagaoka, M., Clark, B.S., Collery, R.F., Si-Tayeb, K., and Duncan, S.A. (2012). JD induced pluripotent stem cell-derived hepatocytes faithfully recapitulate the pathophysiology of familial hypercholesterolemia. Hepatology *56*, 2163–2171.

Chen, R., Jiang, X., Sun, D., Han, G., Wang, F., Ye, M., Wang, L., and Zou, H. (2009). Glycoproteomics analysis of human liver tissue by combination of multiple enzyme digestion and hydrazide chemistry. J. Proteome Res. *8*, 651–661.

Choi, S.M., Kim, Y., Shim, J.S., Park, J.T., Wang, R.H., Leach, S.D., Liu, J.O., Deng, C., Ye, Z., and Jang, Y.Y. (2013). Efficient drug screening and gene correction for treating liver disease using patient-specific stem cells. Hepatology *57*, 2458–2468.

Delaforest, A., Nagaoka, M., Si-Tayeb, K., Noto, F.K., Konopka, G., Battle, M.A., and Duncan, S.A. (2011). HNF4A is essential for specification of hepatic progenitors from human pluripotent stem cells. Development *138*, 4143–4153.

Dorrell, C., Erker, L., Lanxon-Cookson, K.M., Abraham, S.L., Victoroff, T., Ro, S., Canaday, P.S., Streeter, P.R., and Grompe, M. (2008). Surface markers for the murine oval cell response. Hepatology *48*, 1282–1291.

Dorrell, C., Erker, L., Schug, J., Kopp, J.L., Canaday, P.S., Fox, A.J., Smirnova, O., Duncan, A.W., Finegold, M.J., Sander, M., et al. (2011). Prospective isolation of a bipotential clonogenic liver progenitor cell in adult mice. Genes Dev. *25*, 1193–1203.

Ducret, A., Kux van Geijtenbeek, S., Röder, D., Simon, S., Chin, D., Berrera, M., Gruenbaum, L., Ji, C., and Cutler, P. (2015). Identification of six cell surface proteins for specific liver targeting. Proteomics Clin. Appl. *9*, 651–661.

Fox, I.J., Daley, G.Q., Goldman, S.A., Huard, J., Kamp, T.J., and Trucco, M. (2014). Stem cell therapy. Use of differentiated pluripotent stem cells as replacement therapy for treating disease. Science *345*, 1247391.

Ge, X., Yamamoto, S., Tsutsumi, S., Midorikawa, Y., Ihara, S., Wang, S.M., and Aburatani, H. (2005). Interpreting expression profiles of cancers by genome-wide survey of breadth of expression in normal tissues. Genomics *86*, 127–141.

Gundry, R.L., Riordon, D.R., Tarasova, Y., Chuppa, S., Bhattacharya, S., Juhasz, O., Wiedemeier, O., Milanovich, S., Noto, F.K., Tchernyshyov, I., et al. (2012). A cell surfaceome map for immunophenotyping and sorting pluripotent stem cells. Mol. Cell Proteomics *11*, 303–316.

Hay, D.C., Zhao, D., Fletcher, J., Hewitt, Z.A., McLean, D., Urruticoechea-Uriguen, A., Black, J.R., Elcombe, C., Ross, J.A., Wolf, R., et al. (2008). Efficient differentiation of hepatocytes from human embryonic stem cells exhibiting markers recapitulating liver development in vivo. Stem Cells *26*, 894–902.

Holtzinger, A., Streeter, P.R., Sarangi, F., Hillborn, S., Niapour, M., Ogawa, S., and Keller, G. (2015). New markers for tracking endoderm induction and hepatocyte differentiation from human pluripotent stem cells. Development *142*, 4253–4265.

Joensuu, T., Hämäläinen, R., Yuan, B., Johnson, C., Tegelberg, S., Gasparini, P., Zelante, L., Pirvola, U., Pakarinen, L., Lehesjoki, A.E., et al. (2001). Mutations in a novel gene with transmembrane domains underlie Usher syndrome type 3. Am. J. Hum. Genet. *69*, 673–684.

Lanier, L.L., Engleman, E.G., Gatenby, P., Babcock, G.F., Warner, N.L., and Herzenberg, L.A. (1983). Correlation of functional properties of human lymphoid cell subsets and surface marker phenotypes using multiparameter analysis and flow cytometry. Immunol. Rev. *74*, 143–160.

Li, J., Ning, G., and Duncan, S.A. (2000). Mammalian hepatocyte differentiation requires the transcription factor HNF-4alpha. Genes Dev. *14*, 464–474.

Mallanna, S.K., and Duncan, S.A. (2013). Differentiation of hepatocytes from pluripotent stem cells. Curr. Protoc. Stem Cell Biol. *26*, Unit 1G.4.

Miki, K., Endo, K., Takahashi, S., Funakoshi, S., Takei, I., Katayama, S., Toyoda, T., Kotaka, M., Takaki, T., Umeda, M., et al. (2015). Efficient detection and purification of cell populations using synthetic microRNA switches. Cell Stem Cell *16*, 699–711.

Morris, S.A., Cahan, P., Li, H., Zhao, A.M., San Roman, A.K., Shivdasani, R.A., Collins, J.J., and Daley, G.Q. (2014). Dissecting engineered cell types and enhancing cell fate conversion via CellNet. Cell *158*, 889–902.

Omasits, U., Ahrens, C.H., Müller, S., and Wollscheid, B. (2014). Protter: interactive protein feature visualization and integration with experimental proteomic data. Bioinformatics *30*, 884–886.

Parviz, F., Matullo, C., Garrison, W.D., Savatski, L., Adamson, J.W., Ning, G., Kaestner, K.H., Rossi, J.M., Zaret, K.S., and Duncan, S.A. (2003). Hepatocyte nuclear factor 4alpha controls the development of a hepatic epithelium and liver morphogenesis. Nat. Genet. *34*, 292–296.

Peters, D.T., Henderson, C.A., Warren, C.R., Friesen, M., Xia, F., Becker, C.E., Musunuru, K., and Cowan, C.A. (2016). Asialoglycoprotein receptor 1 is a specific cell-surface marker for isolating hepatocytes derived from human pluripotent stem cells. Development *143*, 1475–1481.

Rashid, S.T., Corbineau, S., Hannan, N., Marciniak, S.J., Miranda, E., Alexander, G., Huang-Doran, I., Griffin, J., Ahrlund-Richter, L., Skepper, J., et al. (2010). Modeling inherited metabolic disorders of the liver using human induced pluripotent stem cells. J. Clin. Invest. *120*, 3127–3136.

Robinton, D.A., and Daley, G.Q. (2012). The promise of induced pluripotent stem cells in research and therapy. Nature *481*, 295–305.

Si-Tayeb, K., Noto, F.K., Nagaoka, M., Li, J., Battle, M.A., Duris, C., North, P.E., Dalton, S., and Duncan, S.A. (2010a). Highly efficient generation of human hepatocyte-like cells from induced pluripotent stem cells. Hepatology *51*, 297–305.

Si-Tayeb, K., Noto, F.K., Sepac, A., Sedlic, F., Bosnjak, Z.J., Lough, J.W., and Duncan, S.A. (2010b). Generation of human induced pluripotent stem cells by simple transient transfection of plasmid DNA encoding reprogramming factors. BMC Dev. Biol. *10*, 81.

Song, Z., Cai, J., Liu, Y., Zhao, D., Yong, J., Duo, S., Song, X., Guo, Y., Zhao, Y., Qin, H., et al. (2009). Efficient generation of hepatocyte-like cells from human induced pluripotent stem cells. Cell Res. *19*, 1233–1242.

Spangrude, G.J., Heimfeld, S., and Weissman, I.L. (1988). Purification and characterization of mouse hematopoietic stem cells. Science *241*, 58–62.

Sullivan, G.J., Hay, D.C., Park, I.H., Fletcher, J., Hannoun, Z., Payne, C.M., Dalgetty, D., Black, J.R., Ross, J.A., Samuel, K., et al. (2010). Generation of functional human hepatic endoderm from human induced pluripotent stem cells. Hepatology *51*, 329–335.

Sun, Z., Sun, D., Wang, F., Cheng, K., Zhang, Z., Xu, B., Ye, M., Wang, L., and Zou, H. (2014). Differential analysis of N-glycoproteome between hepatocellular carcinoma and normal human liver tissues by combination of multiple protease digestion and solid phase based labeling. Clin. Proteomics *11*, 26.

Tafaleng, E.N., Chakraborty, S., Han, B., Hale, P., Wu, W., Soto-Gutierrez, A., Feghali-Bostwick, C.A., Wilson, A.A., Kotton, D.N., Nagaya, M., et al. (2015). Induced pluripotent stem cells model personalized variations in liver disease resulting from α1-antitrypsin deficiency. Hepatology *62*, 147–157.

Teslovich, T.M., Musunuru, K., Smith, A.V., Edmondson, A.C., Stylianou, I.M., Koseki, M., Pirruccello, J.P., Ripatti, S., Chasman, D.I., Willer, C.J., et al. (2010). Biological, clinical and population relevance of 95 loci for blood lipids. Nature *466*, 707–713.

Uhlen, M., Oksvold, P., Fagerberg, L., Lundberg, E., Jonasson, K., Forsberg, M., Zwahlen, M., Kampf, C., Wester, K., Hober, S., et al. (2010). Towards a knowledge-based human protein atlas. Nat. Biotechnol. *28*, 1248–1250.

Uhlén, M., Fagerberg, L., Hallström, B.M., Lindskog, C., Oksvold, P., Mardinoglu, A., Sivertsson, Å., Kampf, C., Sjöstedt, E., Asplund, A., et al. (2015). Proteomics. Tissue-based map of the human proteome. Science *347*, 1260419.

Wilhelm, M., Schlegl, J., Hahne, H., Moghaddas Gholami, A., Lieberenz, M., Savitski, M.M., Ziegler, E., Butzmann, L., Gessulat, S., Marx, H., et al. (2014). Mass-spectrometry-based draft of the human proteome. Nature *509*, 582–587.

Wollscheid, B., Bausch-Fluck, D., Henderson, C., O'Brien, R., Bibel, M., Schiess, R., Aebersold, R., and Watts, J.D. (2009). Mass-spectrometric identification and relative quantification of N-linked cell surface glycoproteins. Nat. Biotechnol. *27*, 378–386.

Yan, H., Zhong, G., Xu, G., He, W., Jing, Z., Gao, Z., Huang, Y., Qi, Y., Peng, B., Wang, H., et al. (2012). Sodium taurocholate cotransporting polypeptide is a functional receptor for human hepatitis B and D virus. Elife *1*, e00049.

Yang, W., Liu, Y., Slovik, K.J., Wu, J.C., Duncan, S.A., Rader, D.J., and Morrisey, E.E. (2015). Generation of iPSCs as a pooled culture using magnetic activated cell sorting of newly reprogrammed cells. PLoS One *10*, e0134995.

Yu, Y., Liu, H., Ikeda, Y., Amiot, B.P., Rinaldo, P., Duncan, S.A., and Nyberg, S.L. (2012). Hepatocyte-like cells differentiated from human induced pluripotent stem cells: relevance to cellular therapies. Stem Cell Res. *9*, 196–207.

Zhu, J., Sun, Z., Cheng, K., Chen, R., Ye, M., Xu, B., Sun, D., Wang, L., Liu, J., Wang, F., et al. (2014). Comprehensive mapping of protein N-glycosylation in human liver by combining hydrophilic interaction chromatography and hydrazide chemistry. J. Proteome Res. *13*, 1713–1721.

Stem Cell Reports, Volume 7

Supplemental Information

Mapping the Cell-Surface N-Glycoproteome of Human Hepatocytes Reveals Markers for Selecting a Homogeneous Population of iPSC-Derived Hepatocytes

Sunil K. Mallanna, Max A. Cayo, Kirk Twaroski, Rebekah L. Gundry, and Stephen A. Duncan

Figure S1, related to Figure 2. Bar graph showing results of DAVID analysis of 300 proteins identified in primary hepatocytes by cell surface capture. Bar graphs showing a representation of the top 10 proteins categorized using SwissProt keywords **(A)**, and gene ontology molecular function terms **(B)** that were enriched in the cell surface capture (CSC) data compared to gene distribution in the human genome (Y-axis). Numbers in parenthesis represent the number of proteins out of a total of 300 identified by CSC that fall under the indicated annotation. Enrichment of each classification is expressed as a log10 p value on the X-axis.

A

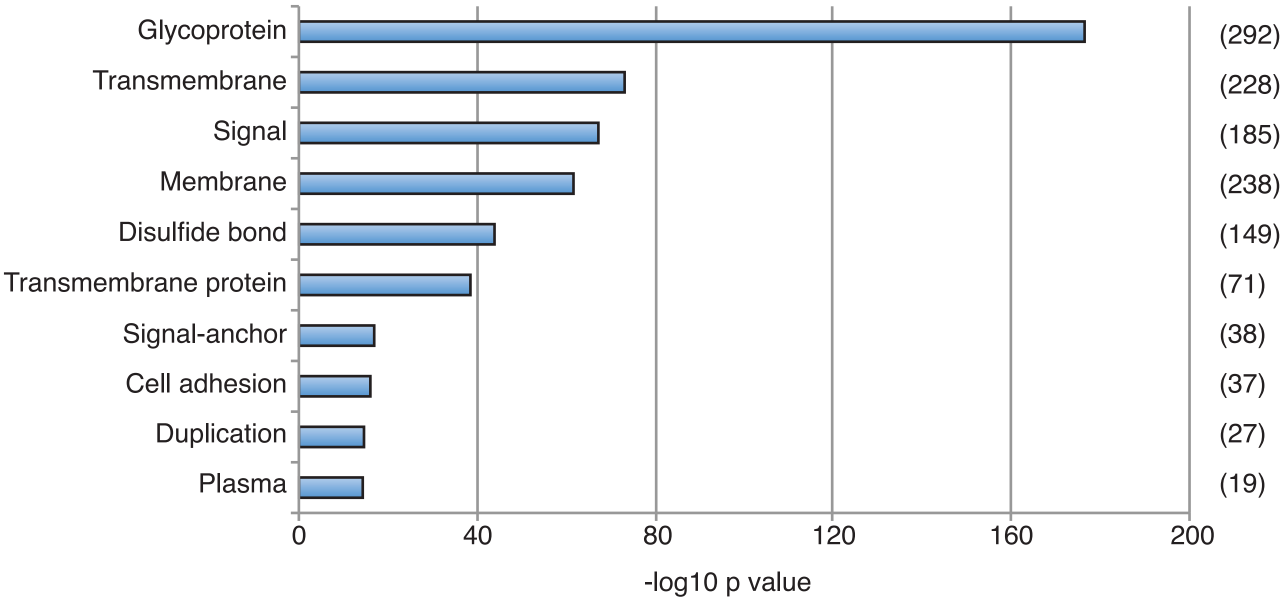

B

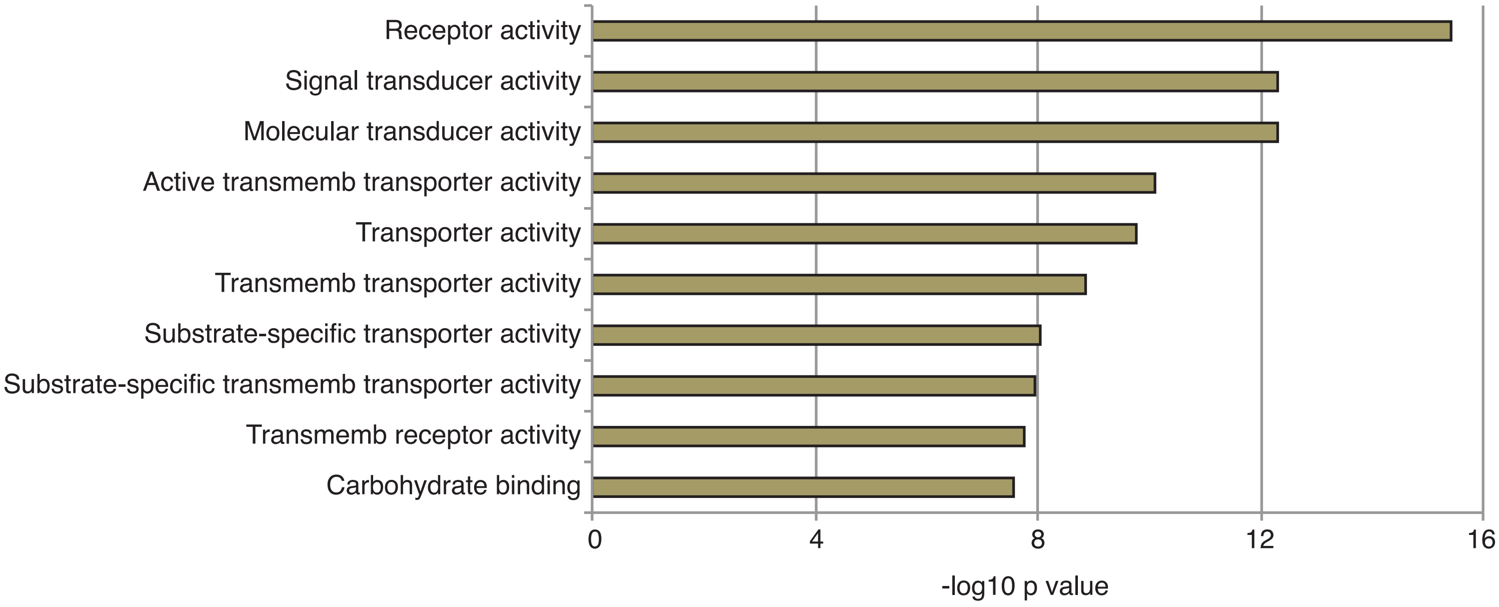

Figure S2, related to Figure 2 and 3. Representative annotated MS/MS Spectra for three candidate proteins.

SLC10A1

Sequence: MEAHNASAPFNFTLPPNFGK, N5-Deamidated (0.98402 Da), N11-Deamidated (0.98402 Da), M1-Oxidation (15.99492 Da)
Charge: +3, Monoisotopic m/z: 736.67346 Da (-0.27 mmu/-0.36 ppm), MH+: 2208.00583 Da, RT: 57.5920 min,
Identified with: Sequest HT (v1.17); XCorr:3.24, Percolator q-Value:0, Percolator PEP:0.0109, Ions matched by search engine: 0/0
Fragment match tolerance used for search: 0.6 Da

Fragment Matches

Value Type: Theo. Mass [Da]

Ion Series | Neutral Losses | Precursor Ions

#1	b^+	b^{2+}	b^{3+}	Seq.	y^+	y^{2+}	y^{3+}	#2
1	148.04268	74.52498	50.01908	M-Oxidation				20
2	277.08527	139.04627	93.03327	E	2060.97123	1030.98925	687.66193	19
3	348.12238	174.56483	116.71231	A	1931.92864	966.46796	644.64773	18
4	485.18129	243.09429	162.39862	H	1860.89153	930.94940	620.96869	17
5	600.20824	300.60776	200.74093	N-Deamid...	1723.83261	862.41994	575.28239	16
6	671.24535	336.12631	224.41997	A	1608.80567	804.90647	536.94007	15
7	758.27738	379.64233	253.43064	S	1537.76856	769.38792	513.26104	14
8	829.31449	415.16089	277.10968	A	1450.73653	725.87190	484.25036	13
9	926.36726	463.68727	309.46060	P	1379.69941	690.35335	460.57132	12
10	1073.43567	537.22147	358.48341	F	1282.64665	641.82696	428.22040	11
11	1188.46262	594.73495	396.82572	N-Deamid...	1135.57824	568.29276	379.19760	10
12	1335.53103	668.26915	445.84853	F	1020.55129	510.77928	340.85528	9
13	1436.57871	718.79299	479.53109	T	873.48288	437.24508	291.83248	8
14	1549.66277	775.33502	517.22577	L	772.43520	386.72124	258.14992	7
15	1646.71554	823.86141	549.57670	P	659.35114	330.17921	220.45523	6
16	1743.76830	872.38779	581.92762	P	562.29837	281.65282	188.10431	5

Fragment Spectrum

OT2_Gundry_2013-01-22_SM42-03.raw #8767 RT: 57.5920 min
ITMS, 737.0066@cid35.00, z=+3, Mono m/z=736.67346 Da, MH+=2208.00583 Da, Match Tol.=0.8 Da

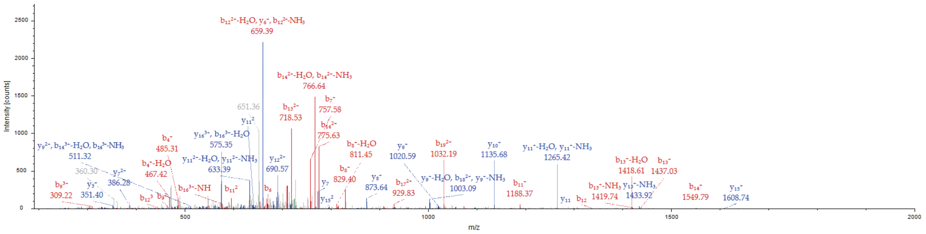

CLRN3

Sequence: DSASNGSIFITY, N5-Deamidated (0.98402 Da)
Charge: +2, Monoisotopic m/z: 638.29095 Da (+0.35 mmu/+0.54 ppm), MH+: 1275.57463 Da, RT: 47.9004 min,
Identified with: Sequest HT (v1.17); XCorr:3.31, Percolator q-Value:0, Percolator PEP:0.000377, Ions matched by search engine: 0/0
Fragment match tolerance used for search: 0.6 Da

Fragment Matches

Value Type: Theo. Mass [Da]

Ion Series | Neutral Losses | Precursor Ions

#1	b^+	b^{2+}	Seq.	y^+	y^{2+}	#2
1	116.03422	58.52075	D			12
2	203.06625	102.03676	S	1160.54700	580.77714	11
3	274.10336	137.55532	A	1073.51497	537.26112	10
4	361.13539	181.07133	S	1002.47785	501.74257	9
5	476.16233	238.58481	N-Deamid...	915.44583	458.22655	8
6	533.18380	267.09554	G	800.41888	400.71308	7
7	620.21583	310.61155	S	743.39742	372.20235	6
8	733.29989	367.15358	I	656.36539	328.68633	5
9	880.36830	440.68779	F	543.28133	272.14430	4
10	993.45237	497.22982	I	396.21291	198.61009	3
11	1094.50005	547.75366	T	283.12885	142.06806	2
12			Y	182.08117	91.54422	1

Fragment Spectrum

OT2_Gundry_2013-01-22_SM42-03.raw #7325 RT: 47.9004 min
ITMS, 638.2898@cid35.00, z=+2, Mono m/z=638.29095 Da, MH+=1275.57463 Da, Match Tol.=0.8 Da

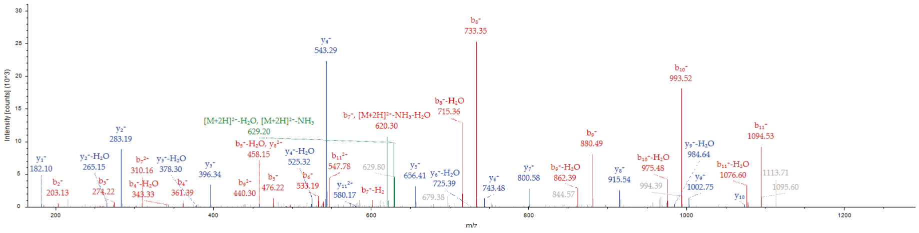

AADAC

Sequence: VVGSFDEVPPTSDENVTVTETK, N15-Deamidated (0.98402 Da)
Charge: +2, Monoisotopic m/z: 1176.06055 Da (+0.08 mmu/+0.07 ppm), MH+: 2351.11382 Da, RT: 38.7348 min,
Identified with: Sequest HT (v1.17); XCorr:5.25, Percolator q-Value:0, Percolator PEP:0.000209, Ions matched by search engine: 0/0
Fragment match tolerance used for search: 0.6 Da

Fragment Matches

Value Type: Theo. Mass [Da]

Ion Series | Neutral Losses | Precursor Ions

#1	b^+	b^{2+}	Seq.	y^+	y^{2+}	#2
1	100.07569	50.54148	V			22
2	199.14410	100.07569	V	2252.04524	1126.52626	21
3	256.16557	128.58642	G	2152.97683	1076.99205	20
4	343.19760	172.10244	S	2095.95537	1048.48132	19
5	490.26601	245.63664	F	2008.92334	1004.96531	18
6	605.29295	303.15011	D	1861.85492	931.43110	17
7	734.33555	367.67141	E	1746.82798	873.91763	16
8	833.40396	417.20562	V	1617.78539	809.39633	15
9	930.45672	465.73200	P	1518.71697	759.86213	14
10	1027.50949	514.25838	P	1421.66421	711.33574	13
11	1128.55717	564.78222	T	1324.61145	662.80936	12
12	1215.58919	608.29824	S	1223.56377	612.28552	11
13	1330.61614	665.81171	D	1136.53174	568.76951	10
14	1459.65873	730.33300	E	1021.50480	511.25604	9
15	1574.68567	787.84648	N-Deamid...	892.46220	446.73474	8
16	1673.75409	837.38068	V	777.43526	389.22127	7

Fragment Spectrum

OT2_Gundry_2013-01-22_SM42-01.raw #5712 RT: 38.7348 min
ITMS, 1176.5618@cid35.00, z=+2, Mono m/z=1176.06055 Da, MH+=2351.11382 Da, Match Tol.=0.8 Da

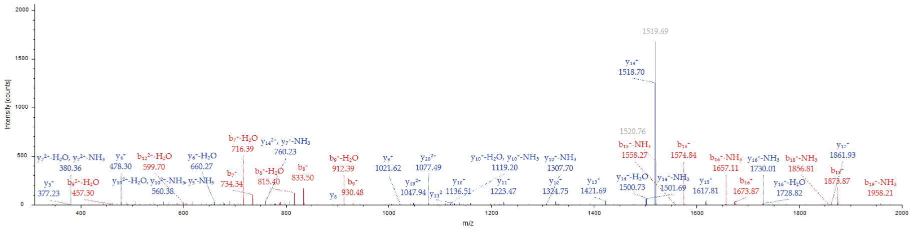

Figure S3, related to Figure 3 and Figure 4. Expression of SLC10A1, CLRN3, and AADAC in hepatocyte-like cells
(A) SLC10A1, CLRN3, and AADAC mRNAs are expressed at lower levels in human ES cell derived hepatocyte-like cells compared to primary hepatocytes. Bar graphs of qRT-PCR results showing levels of mRNAs encoding cell surface proteins SLC10A1, CLRN3, and AADAC in H1 and H9 human ES Cell derived hepatocyte-like cells in comparison to primary hepatocytes. ASGR1 and Albumin mRNA levels are included as reference points. Data from H1 and H9 human ES cell derived hepatocyte-like cells are from a single differentiation. Data from primary hepatocytes is presented as a mean of 3 independent samples.
(B and C) A subpopulation of iPSC-derived hepatocyte-like cells co-express SLC10A1, CLRN3, and AADAC. Micrographs showing the results of immunocytochemistry to detect co-staining of cell surface proteins SLC10A1 (clone C3B6 mouse monoclonal, red), CLRN3 (rabbit polyclonal, green), and AADAC (rabbit polyclonal, green) in K3 iPSC-derived hepatocyte-like cells (B), and SV20 iPSC-derived hepatocyte-like cells (C). Nuclei are identified by DAPI staining. Scale bars = 100 um.

A

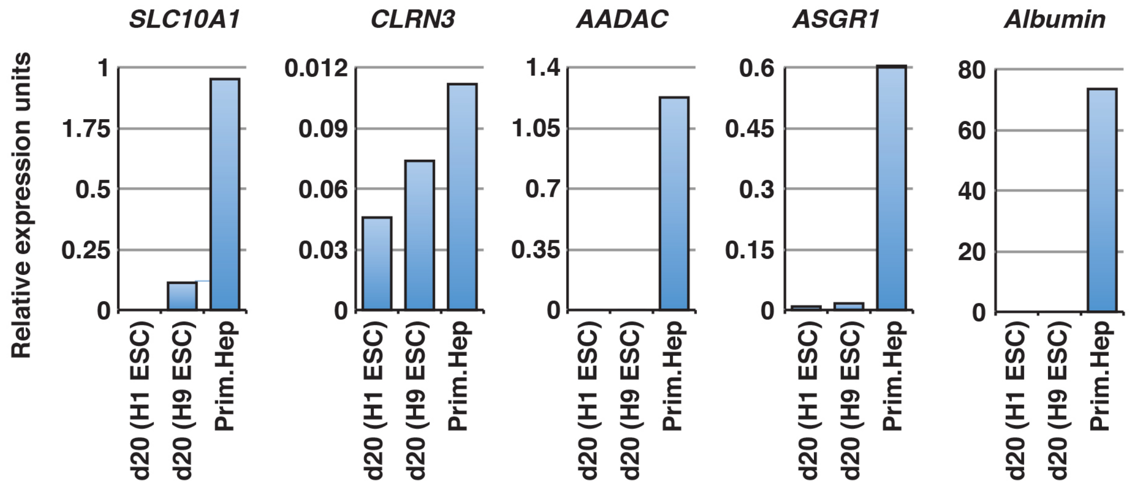

B

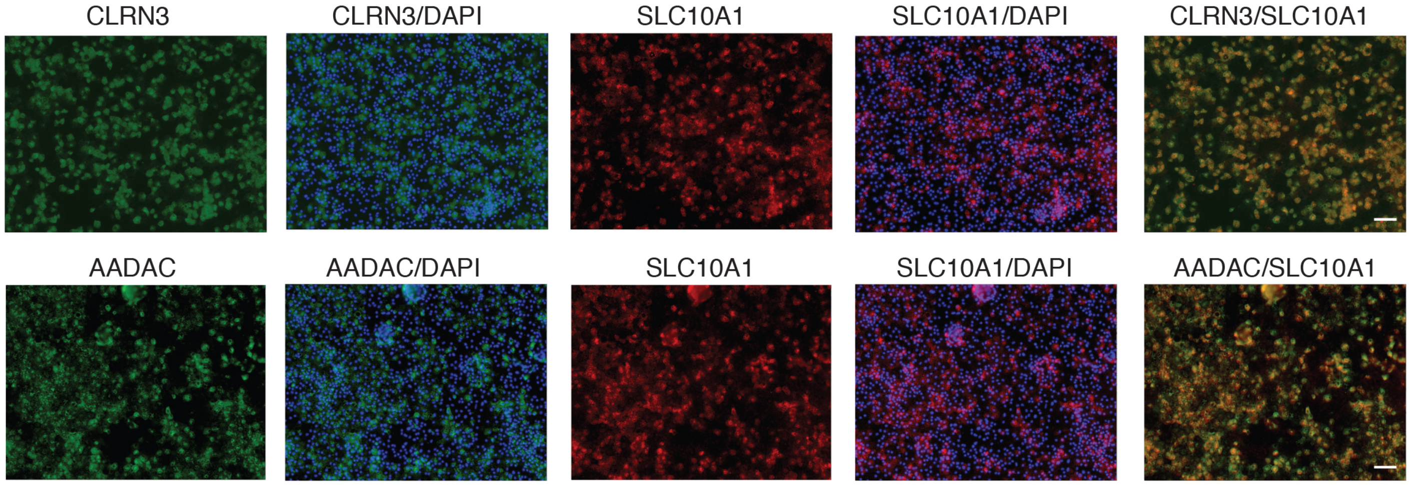

C

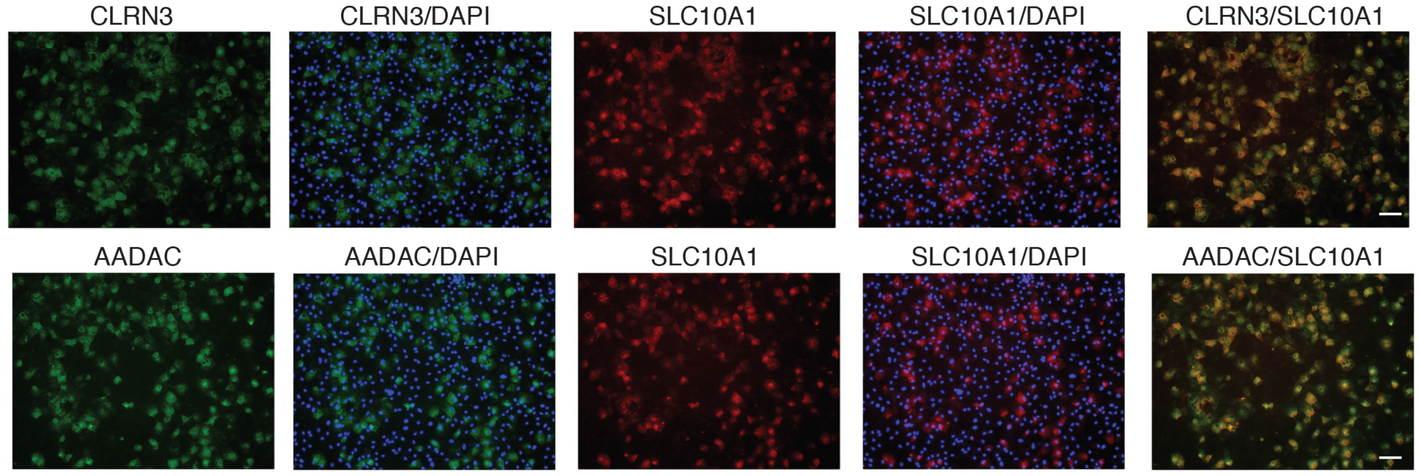

Figure S4, related to Figure 4. ASGR1 is expressed in a subpopulation of iPSC-derived hepatocyte-like cells that co-express SLC10A1, CLRN3, and AADAC. Confocal micrographs showing the results of immunocytochemistry to detect co-staining of ASGR1 (mouse monoclonal, green), with SLC10A1 (rabbit polyclonal, red), CLRN3 (rabbit polyclonal, red), and AADAC (rabbit polyclonal, red) in K3 iPSC-derived hepatocyte-like cells. Nuclei are identified by DAPI staining (blue). White arrows point to representative cells that stained positive with only ASGR1 suggesting less-restrictive expression pattern of ASGR1 in hepatocyte-like cells compared to SLC10A1, CLRN3, and AADAC. Scale bars = 100 um.

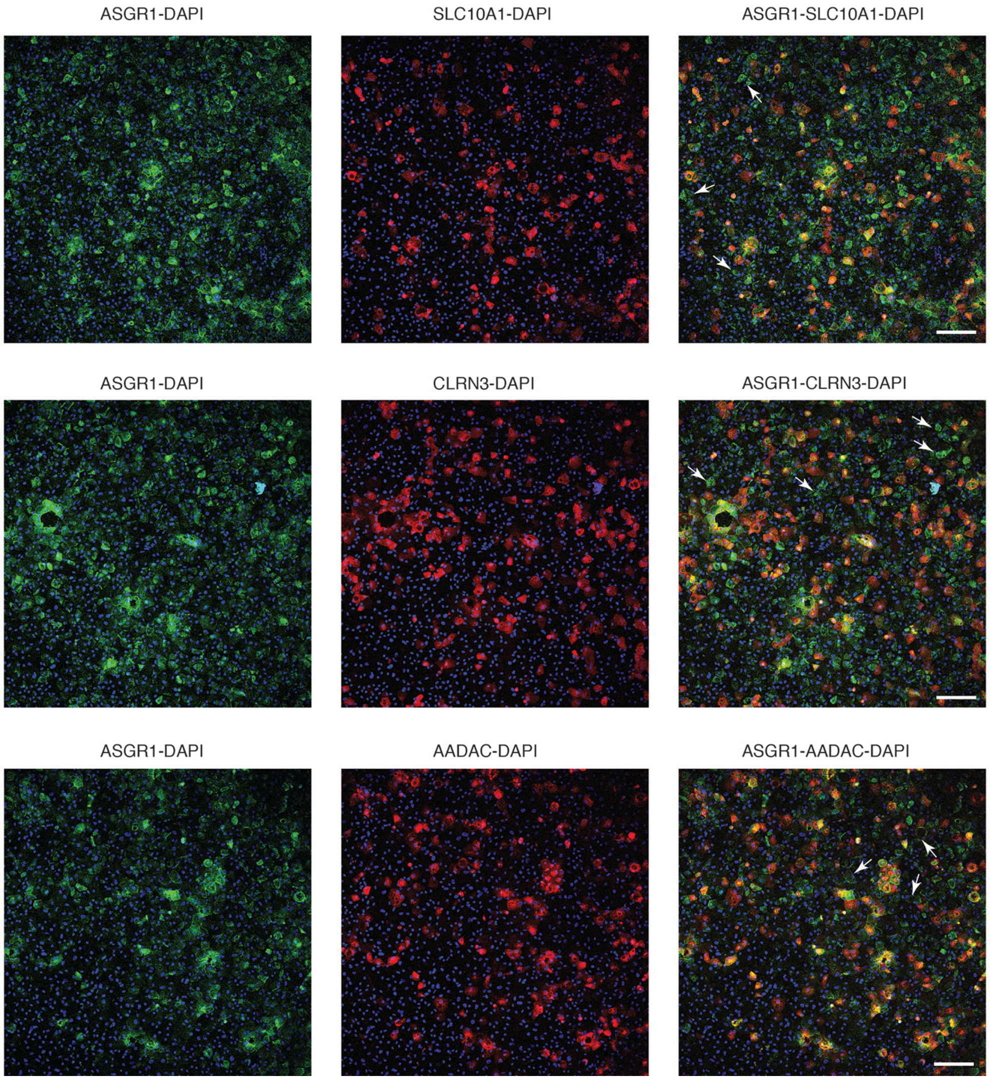

Figure S5, related to Figure 4 and 5. A subpopulation of iPSC-derived hepatocyte-like cells sorted by FACS using antibodies that recognize SLC10A1, CLRN3, or AADAC express several hepatic mRNAs. (A) Dot plot showing gating parameters for FACS sorting SLC10A1 positive and negative iPSC-derived hepatocyte-like cell population. Dead cells are excluded out by staining with DAPI and live cells within the compartment P1 are used for sorting positive and negative cell fractions. No antibody control and PE-conjugated secondary antibody control are used to set up gating parameters for collecting negative cell fraction. iPSC-derived hepatocyte-like cells stained with SLC10A1 antibody and corresponding PE-conjugated secondary antibody is used to set up gating parameters for collecting SLC10A1 positive cell fraction. **(B)** Data in Figure 5 is presented here to demonstrate relative enrichment of selected mRNAs in SLC10A1-positive cell fraction compared to SLC10A1-negative cell fraction which is set to 1. Data is presented as mean and SEM from sorting performed on 3 independent differentiations. **(C and D)** Bar graphs show qRT-PCR analyses to determine relative levels of selected mRNAs in pre-sort, positive sort, and negative sort cell fractons from CLRN3 (C) and AADAC (D) sorting experiments. Data presented is from a single differentiation and sorting experiment.

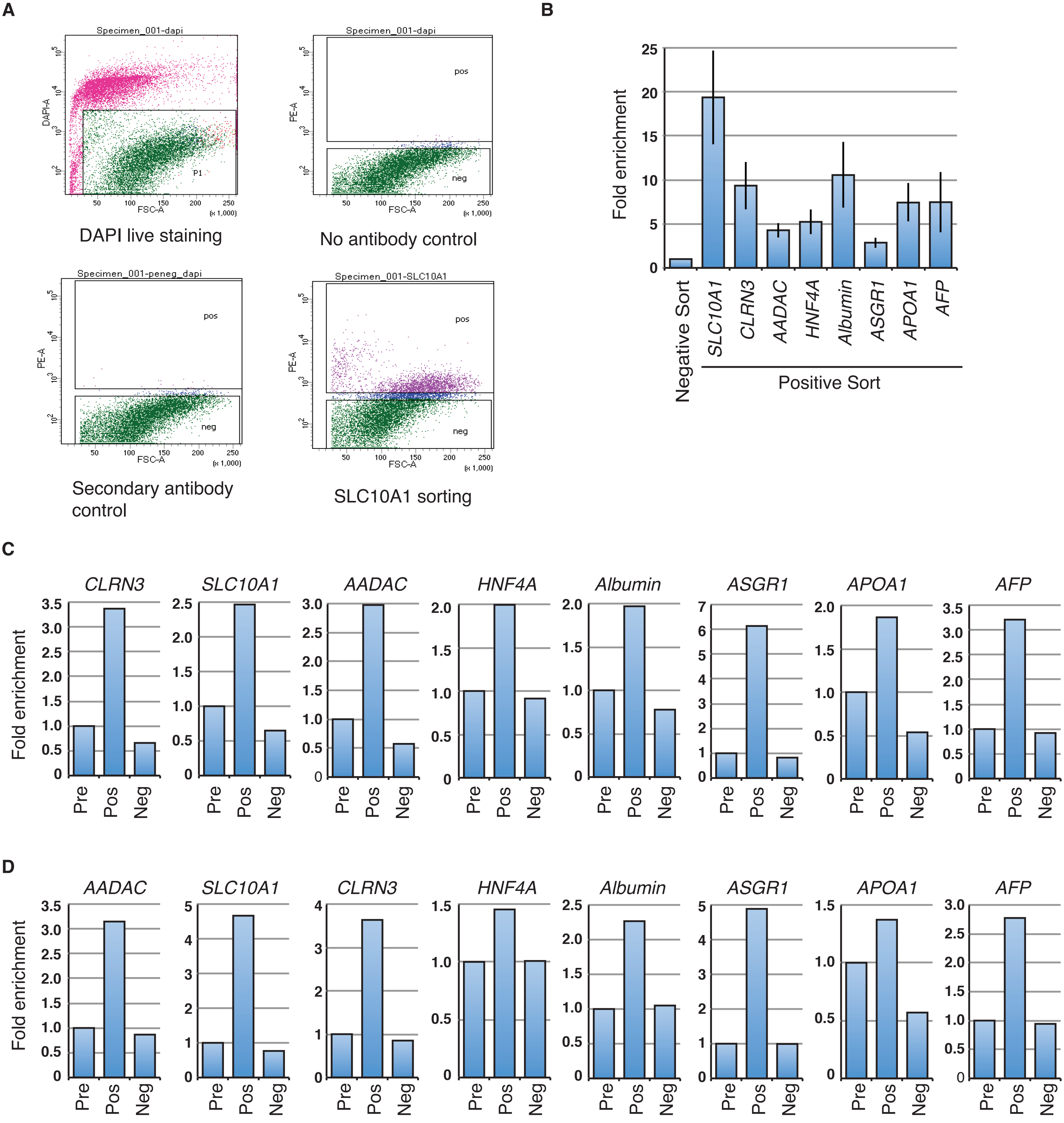

Figure S6, related to Figure 6. CellNet analysis of different fractions of iPSC-derived hepatocyte-like cells FACS sorted using SLC10A1 antibody. (A) Brightfield micrographs showing K3 iPSC-derived hepatocyte-like cells from four independent differentiations used for FACS sorting and microarray analysis in Figure 6. Scale bar = 100 um. **(B)** CellNet analysis to compare GRN of SLC10A1 positive cell fractions of hepatocyte-like cells (n = 4 independent differentiations; pos_1-4) with hepatocyte-like cells differentiated by Cameron et al (Cameron et al., 2015, Stem Cell Reports, 5, 1250-62) on Laminin 111 mix (n = 3 independent differentiations; 1-3) and Laminin 521 (n = 3 independent differentiations; 1-3). Each bar represents data from an independent experiment. **(C)** CellNet analysis to compare GRN of SLC10A1 positive (pos_1-4) and negative cell fractions (neg_1-4) of hepatocyte-like cells from 4 independent differentiations with published array data (Cameron et al., 2015, Stem Cell Reports, 5, 1250-62) from freshly isolated human hepatocytes (n = 3 samples; 1-3). Hepatocyte microarray data were obtained from EMBL-EBI: E-MTAB-3994**.** Each bar represents data from an independent experiment.

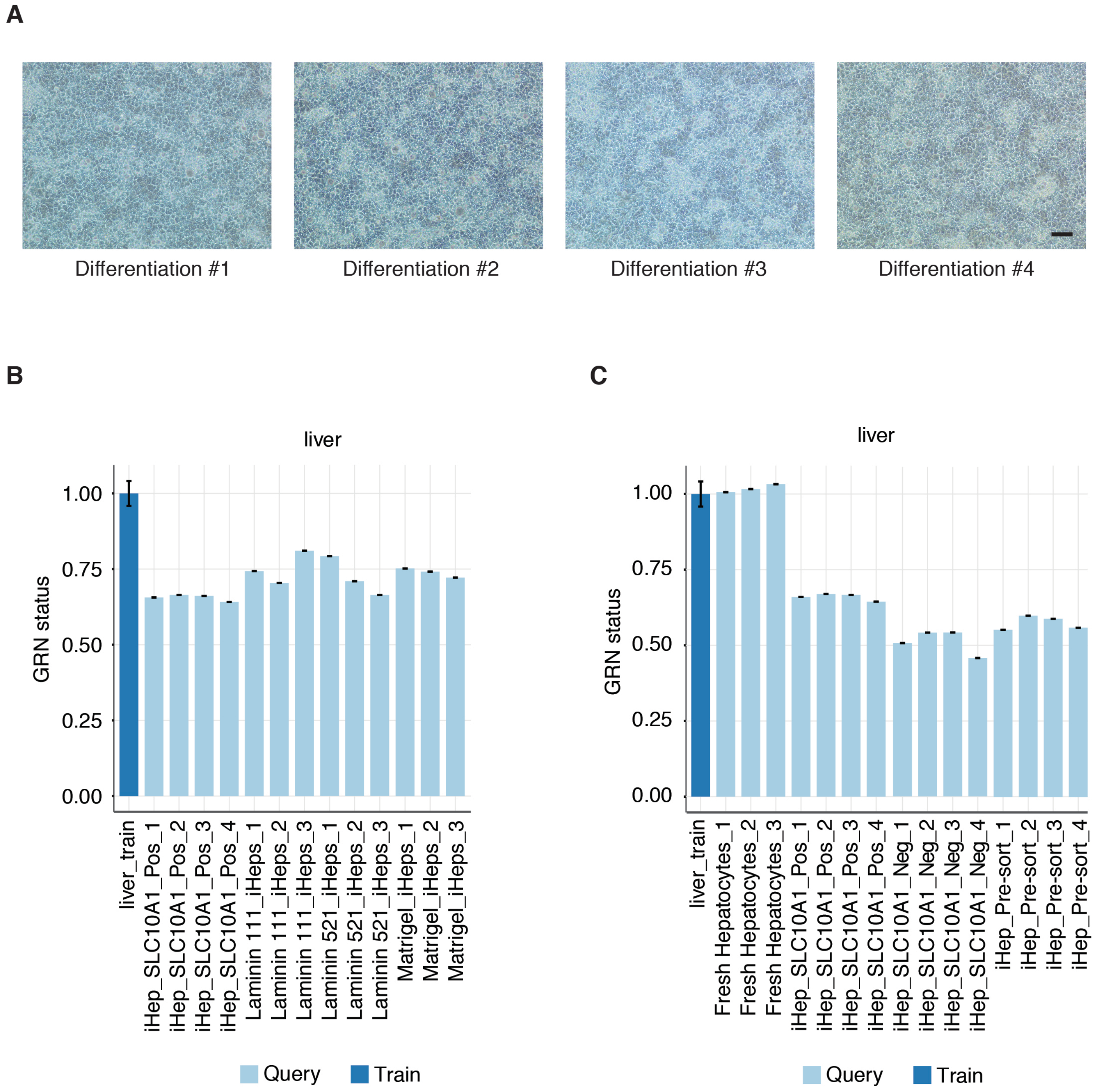

Table S4. List of PrimeTime assays used in qRT-PCR analysis

Gene	Forward	Probe	Reverse
SLC10A1	TGTACAGGAGGAGAGGCATC	AACCTCAGCATTGTGATGACCACCT	ACCTGTCCAATGTCTTCAGTC
CLRN3	GAGCTCTGGGTTTACCTTCTAC	AGCATCAGCAACCCTTACCAGACA	GTGTTCGCCACAAACAGTATC
AADAC	GCCATGGAGAATGATGTGGATA	ACATTTGTGGAGCTCCTGGGACTT	AAAGCTCCCGACAACCTTAAA
HNF4a	TGGACAAAGACAAGAGGAACC	CAAGAAATGCTTCCGGGCTGGC	ATAGCTTGACCTTCGAGTGC
Albumin	AAATCCCACTGCATTGCCGAAGTG	TGCCTGCTGACTTGCCTTCATTAGCT	AGCAGCAGCACGACAGAGTAATCA
ASGR1	TCCTTTCTGAGCCATTGCC	CGTGAAGCAGTTCGTGTCTGACCT	TGAAGTCGCTAGAGTCCCAG
ApoA1	CTTTGAGCACATCCACGTACA	CTGCCAGAAATGCCGAGCCTG	GCCGTGCTCTTCCTGAC
AFP	CTGCAATTGAGAAACCCACTG	TTGGAGAAGTACGGACATTCAGACTGC	TTCCCTCTTCACTTTGGCTG

Supplemental Experimental Procedures

Mass Spectrometry and Data Analysis. Peptides from the CSC-Technology were analyzed over three technical replicate injections on an LTQ Orbitrap Velos as described in PMID: 25068131. MS/MS spectra were processed using Proteome Discoverer 2.2 against the UniProt Human database. SequestHT and MSAmanada search algorithms were used and followed by Percolator (default settings) for post-search processing. Precursor mass tolerance was 10 ppm, fragment mass tolerance was 0.6 Da., and 2 missed cleavages were allowed. Allowed variable modifications were deamidation (N), N-terminal acetylation, and oxidation (M). Carbamidomethylation (C) was a fixed modification. To be considered in the final dataset, proteins had to be deemed "high confidence" based on either SequestHT or MSAmanada, and identified by at least 3 peptide spectrum matches.

Immunostaining. iPS cell-derived hepatocyte-like cells were washed once with 1x PBS, fixed with 4% PFA for 20 mins at room temperature followed by washing with 1x PBS for 3 x 5 mins. For staining cell surface proteins, fixed cells were blocked with 3% BSA in 1x PBS for 60 mins at room temperature followed by incubation with primary antibody against desired cell surface protein overnight at 4°C. For co-staining cell surface proteins with intracellular protein HNF4A, fixed cells were incubated with primary antibody against cell surface protein as described above, fixed again with 4% PFA for 20 mins at room temperature, washed with 1x PBS for 3 x 5 mins. Fixed cells were permeabilized with 0.4% Triton-X for 20 minutes at room temperature and washed with 1x PBS for 3 x 5 mins. Permeabilized cells were blocked with 3% BSA in 1x PBS for 60 mins at room temperature followed by incubation with primary antibody against HNF4A overnight at 4°C. Cells incubated with primary antibody were washed with 1x PBS for 3 x 5 mins, incubated with appropriate secondary antibody for 60 mins at room temperature, washed again with 1x PBS for 3 x 5 mins and imaged. Primary antibodies used were SLC10A1 (Aviva, ARP42097_P050, 1:200; mouse monoclonal, 1:50), CLRN3 (Thermo Fisher Scientific, PA5-26137, 1:200), AADAC (LSBio, LS-C155827, 1:200), ASGR1 (BD Biosciences, 563654, 1:100), and HNF4 (Santa Cruz, sc-6556, 1:250). Secondary antibodies used were A11055 Alexa fluor anti-goat 488 nm, A21207 Alexa fluor anti-rabbit 594 nm, A21206 Alexa fluor anti-rabbit 488 nm, A21203 Alexa fluor anti-mouse 594 nm. Secondary antibodies were used at 1:500 and nuclear stain DAPI (D1306, Thermo Fisher Scientific, 5 mg/ml) was used at 1:5000. Images were processed identically using Adobe Photoshop to optimize brightness and contrast.

FACS sorting iPSC-derived hepatocyte-like cells using SLC10A1 antibody. iPS cell-derived hepatocyte-like cells in 6-well plates were washed once with Versene and incubated in 1 ml of Versene per well at 37°C for 20-25 mins until cell to cell contact looks loose when looking through microscope. Add 1 ml of pre-warmed DMEM to each well, remove Versene-DMEM mixture, and add 1 ml of pre-warmed Collagenase mixture (2 mg/ml Collagenase XI, 1 mg/ml dispase, 100 U/ml DNAse, 0.2% DMSO in 50:50 mixture of HBM and DMEM mixture). Incubate cells in Collagenase mixture at 37°C for ~35-40 mins. During incubation pipette cells 2-3 times using 1 ml pipette to break up clumps to aid dissociation. Incubation with Collagenase mixture should result in cell clumps smaller than 5 cells. Add 500 ul of Versene to each well, collect Collagenase-Versene mixture in a 1.5 ml tube and centrifuge at 400g for 5 mins at RT. Resuspend cell pellet in each 1.5 ml tube in 500-750 ul of Versene, and transfer cell suspension to an suspension culture plate and incubate at 37°C for ~45 mins. During incubation, pipette cells 3 times using 5 ml serological pipette to aid in cell dissociation. Towards the end of Versene incubation, majority of cells should be dissociated to single cells. Filter dissociated cells using 3-4 100 um cell strainers to remove large cell clumps, if any. Collect residual cells struck to the strainer by adding 2 ml of cold 0.5% BSA per strainer. Pool all strained cells and centrifuge at 400g for 5 mins at 4°C. Remove Versene-BSA mixture and block by resuspending in 5-10 ml of 1% BSA, and incubate on ice for ~45 mins. While cells are incubating in blocking mixture, stain cells using trypan blue and determine viability. Aliquot required number of cells for sorting using antibody against SLC10A1 and necessary controls for setting up gating parameters and for collecting gated pre-sort cell fraction (Cell only control, DAPI only control, DAPI + secondary antibody control). Remove blocking buffer by centrifuging at 400g for 5 mins at 4°C. Resuspend cell pellet using 25 ul of 0.25% BSA in 1x PBS per one million cells. To the cells meant to be sorted with SLC10A1 antibody, add 1 ug of SLC10A1 antibody (Aviva, ARP42097_P050) per 1 million cells. Incubate cells at 4°C for 1 hour using an end-to-end rotator. After incubation, wash by resuspending in 1.3 ml of 0.25% BSA in 1x PBS followed by pelleting cells at 400g for 5 mins at 4°C. Resuspend the cell pellet using 25 ul of 0.25% BSA in 1x PBS per 1 million cells and 0.4 ul of donkey anti-rabbit PE (Santa Cruz, sc-3745) per 1 million cells to the sample meant to be sorted with SLC10A1 antibody and DAPI + secondary antibody control sample. Incubate cells at 4°C for 30 mins with flicking tubes every 10 mins to allow proper suspension of cells during incubation. After incubation with secondary antibody, wash cells twice by suspending in 0.25% BSA in 1x PBS followed by pelleting by centrifugation at 400g for 5 mins at 4°C. After washing resuspend the cells in 0.25% BSA in 1x PBS, filter cells using 100 um cell strainers to remove clumps formed by any dead

cells. Finally, add 2 ul of 1:50 diluted 5 mg/ml DAPI stock (D1306, Thermo Fisher Scientific) per 1 million cells to 'DAPI only control', 'DAPI + secondary antibody control', and 'SLC10A1 sort' samples to gate out dead cells. Mixed cells by pipetting gently and cells were sorted using BD FACS Aria IIIu sorter. Cell gating was set to collect live cells from an unstained sample as pre-sort. Dot plot from 'no antibody' and 'secondary antibody only' control samples were used to establish gating parameters for collecting SLC10A1 positive and negative cell fractions. Sorted cells were collected in 1.5 ml tubes containing 100 ul of FBS. Sorted cells were pelleted by centrifugation at 8000 g for 5 mins at 4°C, suspended in RLT plus buffer from RNeasy plus micro kit, flash froze in liquid nitrogen, and stored at -80°C for future use for preparing RNA.

FACS sorting iPSC-derived hepatocyte-like cells using CLRN3 or AADAC antibody. General protocol for FACS sorting iPSC-derived hepatocyte-like cells using CLRN3 or AADAC antibody is similar to sorting protocol described above using SLC10A1 antibody. For sorting CLRN3 positive hepatocyte-like cells, 2 ug of CLRN3 antibody (Thermo Scientific, PA5-26137) per 1 million cells was used. For sorting AADAC positive hepatocyte-like cells, 2 ug of AADAC antibody (LSBio, LS-C155827) per 1 million cells was used. For sorting using CLRN3 or AADAC antibodies, DAPI staining was not done and cell gating was set to collect cells from an unstained sample as pre-sort. Dot plot from 'no antibody' and 'secondary antibody only' control samples were used to establish gating parameters for collecting positive and negative cell fractions.

Statistical Analyses. Student's t-test (two-tailed, unpaired, equal variance) was used when comparing two different conditions/samples and ANOVA-1 way was used when comparing more than two conditions/samples.

Z-score analysis. For identifying transcripts that increased during maturation stage of the differentiation protocol presented in Figure 3A, previously published microarray data from our laboratory on H9 undifferentiated and day 5, 10, 15, and 20 hepatic differentiated cells were used (samples 1 and 2 from GEO database accession numbers GSE14897 and GSE25417). Fold change in transcript levels between days 10 and 15, and days 15 and 20 were calculated by ANOVA-1 way analysis. Z-score value was calculated using mean and standard deviation of fold change for the entire data set. Z-score value of 3 corresponding to 9.27 and 5.06 fold enrichment between days 10 and 15, and days 15 and 20, respectively, was used as cut off.
